# Development of benzofuran-derived sulfamates as dual aromatase-steroid sulfatase inhibitors (DASIs): design, synthesis and biological evaluation†

**DOI:** 10.1039/d4md00795f

**Published:** 2025-01-09

**Authors:** Ahmed G. Eissa, Francesca Gozzi, Oqab Aloqab, Charlotte E. Parrish, Nadira Mohamed, Irene Shiali, Harith Al-Baldawi, Paul A. Foster, Claire Simons

**Affiliations:** a School of Pharmacy and Pharmaceutical Sciences, Cardiff University King Edward VII Avenue Cardiff CF10 3NB UK simonsc@cardiff.ac.uk; b College of Pharmacy, Al Ain University Abu Dhabi United Arab Emirates; c Department of Medicinal Chemistry, Faculty of Pharmacy, Zagazig University Zagazig P.C. 44519 Egypt; d Department of Metabolism and Systems Science, School of Medical Sciences, College of Medicine and Health, University of Birmingham Birmingham B15 2TT UK

## Abstract

Resistance of oestrogen receptor-positive (ER+) breast cancer, the most prevalent type of breast cancer accounting for ∼70% of all cases, to current therapies necessitates the study of alternative strategies. One promising strategy is the multi-targeting approach using dual aromatase-steroid sulfatase inhibitors (DASIs). Herein, we describe the development of DASIs using a common benzofuran pharmacophore. Triazole benzofuran sulfamates were found to have low nM aromatase (Arom) inhibitory activity but no steroid sulfatase (STS) inhibitory activity (IC_50_ > 10 μM); by contrast, benzofuran ketone sulfamates demonstrated low nM STS inhibitory activity but no Arom inhibitory activity (IC_50_ > 1 μM). The addition of a methyl group at the 3rd position of the benzofuran ring in the benzofuran ketone sulfamate 19 (R^1^ = CH_3_) had a notable effect, resulting in dual aromatase and STS inhibitory activities with the 4-chloro derivative 19b (Arom IC_50_ = 137 nM, STS IC_50_ = 48 nM) and 4-methoxy derivative 19e (Arom IC_50_ = 35 nM, STS IC_50_ = 164 nM) optimal for dual inhibition. Arom/STS inhibition results combined with molecular dynamics studies provided a clear rationale for the activity observed.

## Abstract 2

Dual aromatase sulfatase inhibitors (DASIs) were developed from a common benzofuran pharmacophore resulting in potent low nanomolar inhibitors, which combined with molecular dynamics studies provided a clear rationale for the activity observed.

## Graphical abstract

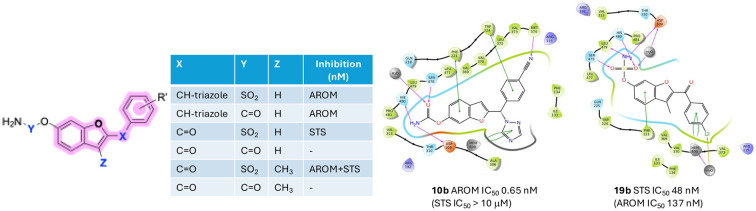

## Introduction

Considering the treatment of breast cancer, multi-targeting is a very popular research approach owing to the development of drug resistance. Thus, a wide variety of multi-targeted drugs have been developed. Oestrogen receptors (ERs), HER-2, EGFR, VEGFR, PI3K, mTOR, 17β-HSD1, aromatase (CYP19A1) and steroid sulfatase (STS) have all been targets for the development of dual acting inhibitors.^1–3^ Each of the dual inhibitor target combinations was inspired by the crucial role played by the target in the viability of tumours in different subclasses of breast cancer.

**Fig. 1:**
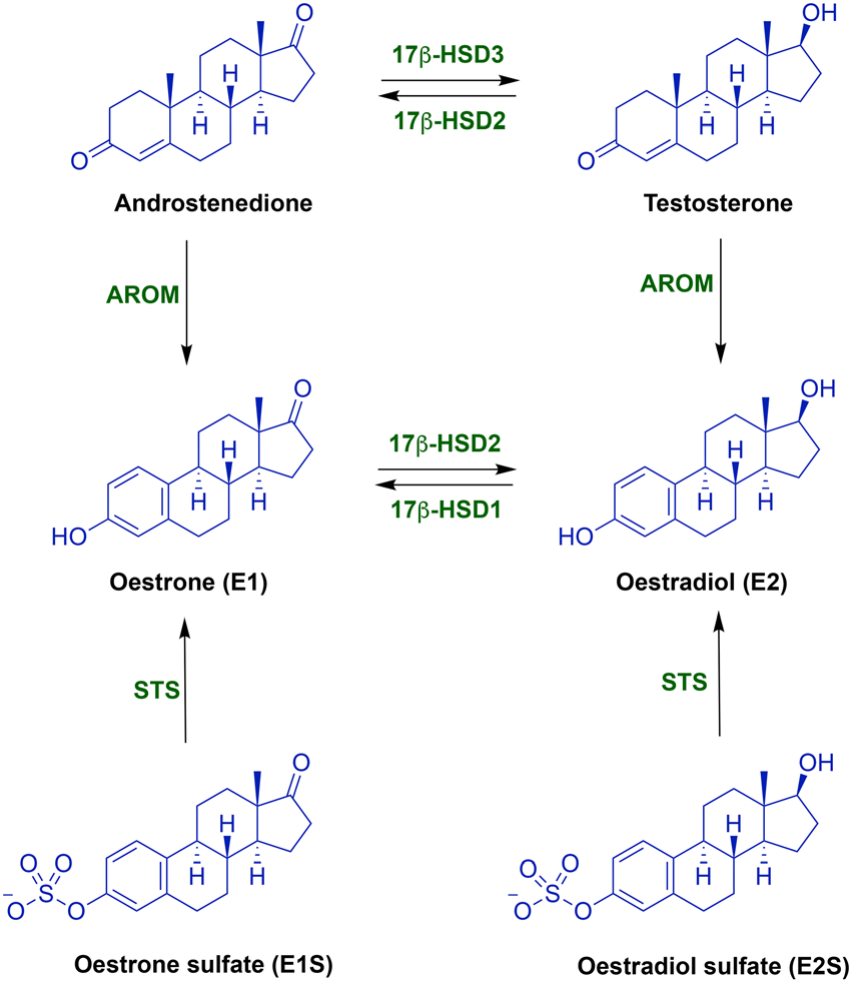
Biosynthesis of oestrogenic and androgenic steroids. AROM = aromatase (CYP19A1), STS = steroid sulfatase, and 17β-HSD = 17β-hydroxysteroid dehydrogenase (types 1, 2 and 3).

In ER-positive (ER+) breast cancer, which accounts for ∼70% of all breast cancers,^4^ oestrogens promote tumour growth.^5^ Approximately ten times more oestrone (E1) is derived from oestrone sulfate (E1S) *via* STS than that from androstenedione *via* aromatase.^6,7^ Therefore, the simultaneous dual inhibition of oestrogen synthesis from androgens through aromatase and oestrogen sulfates (E1S and E2S) through STS (Fig. 1) was an interesting approach, leading to the development of several dual aromatase/STS inhibitors (DASIs).^8,9^

**Fig. 2:**
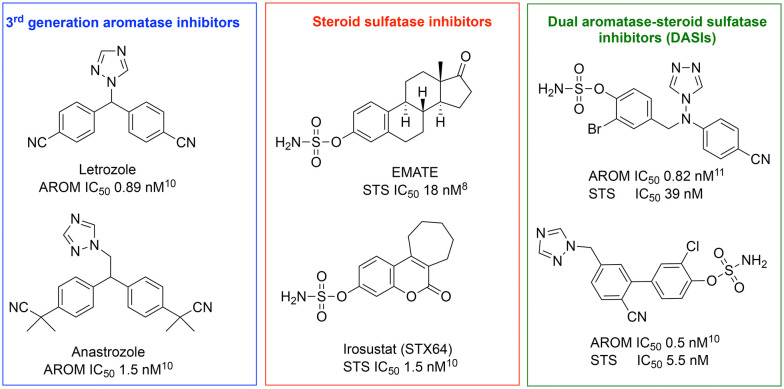
Potent dual aromatase-steroid sulfatase inhibitors (DASIs) based on third-generation aromatase inhibitors, letrozole and anastrozole, and steroid sulfatase inhibitors.^8,10,11^

Merging the active pharmacophores for inhibition of the two enzymes was achieved *via* incorporation of a phenol sulfamate moiety responsible for STS activity into an aromatase inhibitor scaffold depending upon X-ray structures of enzyme–ligand complexes, docking and extensive structure–activity relationship (SAR) studies. Various DASIs were developed over time with activity ranging from a reasonable nanomolar range to outstanding picomolar values for DASIs developed from letrozole and anastrozole aromatase inhibitors and STS inhibitors (Fig. 2).^8–14^

**Fig. 3:**
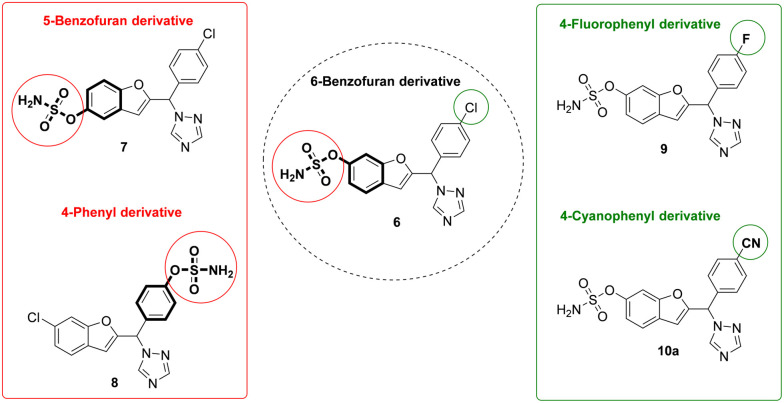
Two sets of comparisons with the structure variations represented in bold; varying position of sulfamate indicated in red and varying phenyl substituent indicated in green.

We have previously described potent aromatase inhibitors with a benzofuran/triazole scaffold,^15–17^ and the research described herein uses the design-in method to build DASIs through incorporation of the sulfamoyl group, required for STS activity, into the phenol scaffold of aromatase inhibitors (Fig. 3). The general idea was to compare three different positions for the sulfamate group: C6 of the benzofuran ring, C5 of the benzofuran ring and C4 of the phenyl ring. Moreover, varying the substituent on the C4 position of the phenyl ring in the 6-benzofuran derivative from chloro to fluoro or nitrile groups has been previously identified as optimal for aromatase inhibitory activity^16,17^ to help build a clearer SAR (Fig. 3).

## Results and discussion

### Triazole benzofuran sulfamates and carbamates

**Fig. 4:**
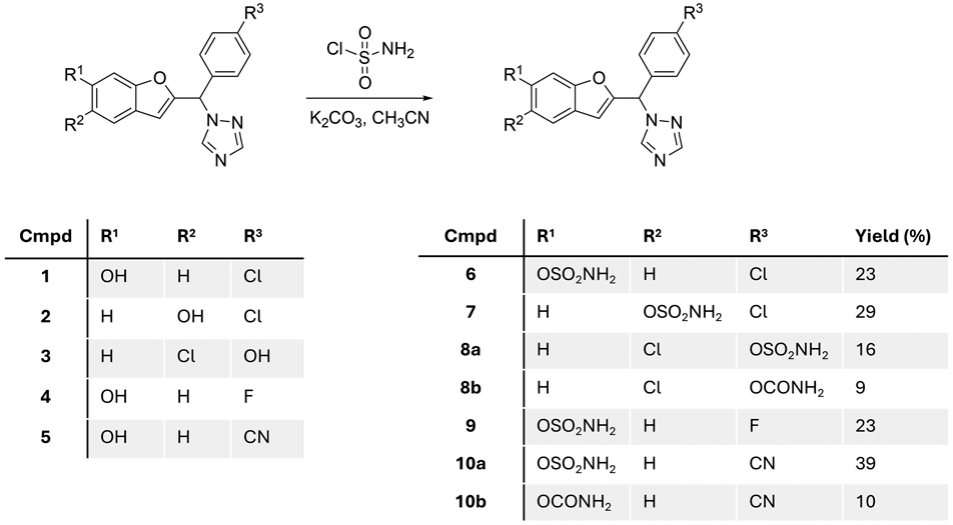
Prepared benzofuran triazole sulfamate and carbamate derivatives 6–10.

Starting with the phenol scaffold of the aromatase inhibitors,^15–17^ preparing the DASI involved two consecutive steps. First sulfamoyl chloride was prepared *in situ* from the reaction of chlorosulfonyl isocyanate and formic acid^13,18^ and used without further purification for reaction with phenolic compounds 1–5 (ref. 15–17) to produce the required sulfamate compounds 6–10 (Fig. 4).

Five different sulfamate derivatives were successfully prepared through this synthetic pathway; however, even with the addition of excess base and/or excess sulfamoyl chloride, the reaction did not reach completion with yields ranging from 16% to 39% (Fig. 4). On two occasions, a carbamate compound was also formed as a side product (8b and 10b), in addition to the expected sulfamate (now labelled as 8a and 10a),which may be attributed to the presence of moisture in the *in situ* preparation of sulfamoyl chloride, resulting in the hydrolysis of the isocyanate starting material. The identity of the sulfamate compounds was confirmed by ^1^H NMR, which showed the appearance of a broad singlet signal integrating for two protons of the amine group at around 8.0 ppm, with the disappearance of the broad singlet integrating for one proton of the phenolic OH group in the range 9–10 ppm (Fig. S1†). The carbamate side product was identified by ^1^H NMR, which showed the appearance of two separate broad singlet signals each integrating for one proton between 6.5 and 7.5 ppm (Fig. S1†), related to the tautomerism of the amidic group. ^13^C NMR supported this assumption by the presence of an extra quaternary carbon in the carbamate product when compared with the ^13^C NMR spectrum of the sulfamate compound. HRMS of the sulfamate compound and the carbamate side product showed a difference of 36 between the mass ion of the sulfamate and the carbamate. Further verification of the carbamate side product was performed by the intentional synthesis of the carbamate compound using chlorosulfonyl isocyanate/H_2_O ^19^ instead of sulfamoyl chloride and comparing the product from the two methods.

Seven compounds including the two carbamate side products were investigated for their aromatase inhibitory activity at 10 nM using the previously reported modified tritiated water assay.^20^ All compounds, except compound 8a, showed more than 50% aromatase inhibition and were further investigated for IC_50_ confirming retention of aromatase inhibitory activity in the low nanomolar range (Table 1). The results provided some preliminary SARs in terms of position and nature of substituents and showed that the carbamate side products 8b and 10b were more active against the aromatase enzyme. There was clear superiority for the 6-position of benzofuran for the sulfamate group over the 5-benzofuran or the 4-phenyl positions. Moreover, the chloro and cyano derivatives 6 and 10 displayed improved aromatase inhibitory activity compared with the fluoro derivative 9 (Table 1).

**Table 1:** Aromatase inhibitory activity of compounds 6–10

Cmpd	Arom IC_50_ (nM)	95% CI^a^ (nM)
6	1.4	1.060–1.848
7	5.49	—
8a	>10	—
8b	4.1	3.646–4.797
9	8.4	6.672–10.620
10a	1.52	1.427–1.628
10b	0.65	0.603–0.696

a Each data point measured in triplicate and the error in the IC_50_ calculation represented as 95% confidence interval (CI).

**Scheme 1:**
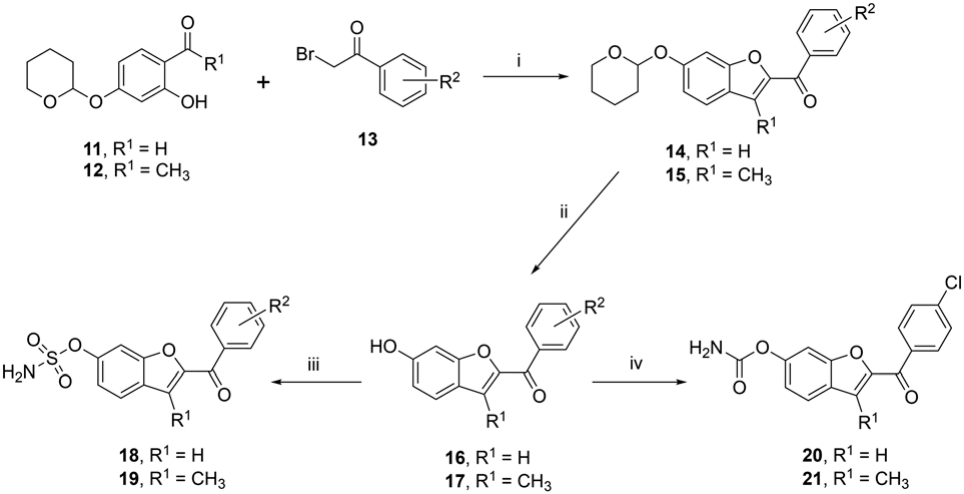
Reagents and conditions: (i) K_2_CO_3_, CH_3_CN, 70 °C, 3 h, (ii) HCl/dioxane, 1 h, (iii) method 1: sulfamoyl chloride, K_2_CO_3_, CH_3_CN or DMF, 16 h. Method 2: sulfamoyl chloride, DMA, 3 h, (iv) chlorosulfonyl isocyanate, CH_3_CN, 1.5–2 h, then H_2_O, 1 h [R^2^: a = 4-F, b = 4-Cl, c = 4-Br, d = 2,4-diCl, e = 4-OCH_3_, f = 4-CN].

Although low nanomolar aromatase activity was observed for the benzofuran triazole derivatives, no inhibitory activity was observed against STS (IC_50_ ≥ 10 μM) using the previously described JEG-3 lysate assay.^21^ A similar profile of nanomolar aromatase inhibition and >10 μM STS inhibitory activity has been reported for vorozole-derived sulfamates having a similar geometry to the benzofuran derivatives described herein,^13,14^ which was suggested to arise from the triazole ring causing steric hindrance within the STS active site. To investigate the relationship between the lack of STS inhibitory activity and the geometric orientation of the compounds 6–10, a series of truncated sulfamate and carbamate compounds 18–21 (Scheme 1), lacking the triazole group but retaining the benzofuran scaffold, were designed based on STX64 (irosustat) (Fig. 1) structural similarity. These truncated compounds had the triazole group replaced by a carbonyl group to offer some planarity and rigidity to the structure.

### Ketone benzofuran sulfamates and carbamates

The ketone sulfamates 18/19 and two carbamate compounds 20/21 were prepared *via* a three-step synthetic pathway. The first step employed a Rap–Stoermer condensation reaction^22,23^ between tetrahydropyran (THP)-protected 2-hydroxybenzaldehyde (11) or THP-protected 2-hydroxyacetophenone (12)^24,25^ and 2-bromo-acetophenone (13) to form the THP-protected benzofuran ketones 14/15. Anomalous behaviour was observed for the 3-methylbenzofuran 2,4-dichloro derivative 15d with the THP deprotected compound 17d in a yield of 45%. The THP protecting in all other derivatives (14/15) was then removed using HCl/dioxane^26^ to give the phenolic derivatives 16/17. Using the *in situ* formation of sulfamoyl chloride described for the preparation of the triazole compounds 6–10 resulted in very low yields (3–7%) of the benzofuran ketone sulfamates 18/19; however, a commercial source was available with improved yields obtained (Table 2) using K_2_CO_3_ as the base and DMF solvent,^18^ although it should be noted that the sulfamoylation reaction did not go to completion even using three molecular equivalents of sulfamoyl chloride.

**Table 2:** Yields and m.p. of ketone sulfamates 18 and 19 and carbamates 20 and 21

Cmpd	R^1^	R^2^	Yield (%)		m.p. (°C)
			Method 1	Method 2	
Sulfamates					
18a	H	4-F	NR	76	200–202
18b	H	4-Cl	18	—	159–161
18c	H	4-Br	46	—	166–168
18d	H	2,4-diCl	33	—	198–200
18e	H	4-OCH_3_	52	—	182–184
18f	H	4-CN	NR	74	200–204
19a	CH_3_	4-F	60	—	181–183
19b	CH_3_	4-Cl	32	—	181–182
19c	CH_3_	4-Br	25	—	178–181
19d	CH_3_	2,4-diCl	NR	22	138–140
19e	CH_3_	4-OCH_3_	20	56	172–176
19f	CH_3_	CN	NR	NR	—
Carbamates					
20	H	4-Cl	58		190–192
21	CH_3_	4-Cl	63		210–212

For derivatives 18a, 18f and 19d, no sulfamate product was obtained using this method (method 1, Table 2); however, using the method of Okada *et al.*,^27^ which employed two equivalents of sulfamoyl chloride, DMA as solvent and no base (method 2, Table 2), the sulfamates were successfully obtained, and in the case of 19e, an improved yield was obtained with method 2 compared with method 1 (56% *vs.* 20%). The preparation of the nitrile derivative 19f using either method 1 or 2 was unsuccessful with no product observed. The carbamates were obtained in good yields on reaction of the phenolic derivatives 16/17 with chlorosulfonyl isocyanate followed by hydrolysis of the (chlorosulfonyl)carbamate intermediate.^19^

The sulfamates 18/19 and carbamates 20/21 were evaluated for inhibitory activity against aromatase^20^ and STS^21^ (Table 3). The sulfamates 18a–f displayed low aromatase inhibitory activity (Arom IC_50_ > 1 μM); however, all displayed sub micromolar STS inhibitory activity (STS IC_50_ 0.115–0.74 μM), with the 4-methoxy derivative 18e (STS IC_50_ 0.115 μM) and 4-nitrile derivative 18f (STS IC_50_ 0.28 μM) optimal.

**Table 3:** Aromatase and STS inhibitory activity of ketone sulfamates 18 and 19 and carbamates 20 and 21

Cmpd	Arom IC_50_ (μM)	95% CI^a^ (μM)	STS IC_50_ (μM)	95% CI^a^ (μM)
18a	>1	—	0.74	0.657–0.828
18b	>1	—	0.41	0.372–0.447
18c	>1	—	0.51	0.385–0.673
18d	>1	—	0.31	0.240–0.399
18e	>1	—	0.115	0.083–0.159
18f	>1	—	0.28	0.198–0.627
19a	0.050	0.037–0.089	0.654	0.471–0.778
19b	0.137	0.114–0.151	0.048	0.023–0.058
19c	0.022	0.012–0.027	0.83	0.516–2.816
19d	0.020	0.013–0.030	1.503	0.937–2.412
19e	0.035	0.018–0.067	0.164	0.089–0.299
20	>1	—	1.22	—
21	>1	—	0.98	—
Letrozole	0.007	—	>1	—
STX64	>1	—	0.006	—

a Each data point measured in triplicate and the error in the IC_50_ calculation represented as 95% confidence interval (CI).

The addition of a methyl group at the 3-position of the benzofuran ring in sulfamates 19a–e had a notable effect, resulting in dual aromatase and STS inhibitory activity with the 4-chloro derivative 19b (Arom IC_50_ 137 nM, STS IC_50_ 48 nM) and 4-methoxy derivative 19e (Arom IC_50_ 35 nM, STS IC_50_ 164 nM) optimal for dual inhibition. The two carbamates 20/21 did not show promising inhibitory activity (Arom/STS IC_50_ ≥ 1 μM).

### Computational studies

#### Aromatase (CYP19A1)

**Fig. 5:**
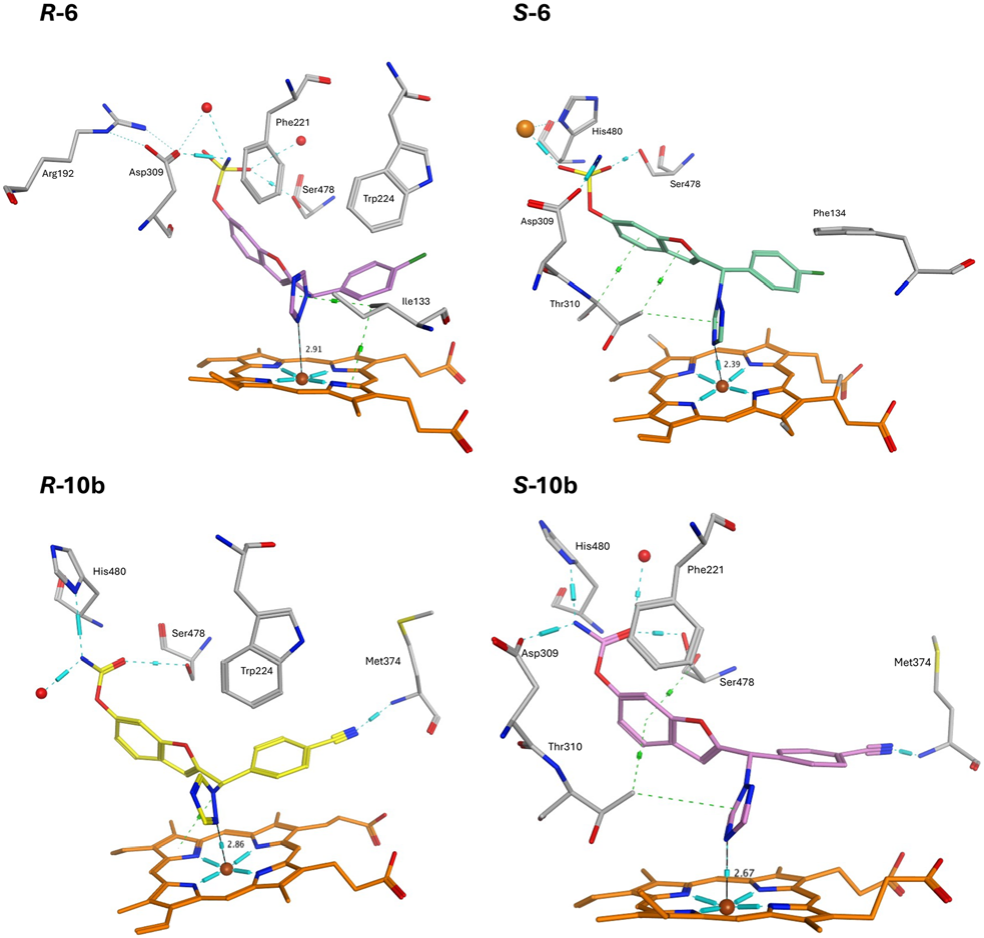
Exemplar triazoles 6 and 10b binding within the CYP19A1 active site after MD simulation (150 ns). Haem is shown in orange with the central iron as a brown sphere, H_2_O molecules shown as red spheres, H-bonding cyan lines/barrels, hydrophobic interactions (van der Waals, π–π stacking) shown as green lines/barrels, and key binding amino acids in light grey.

**Fig. 6:**
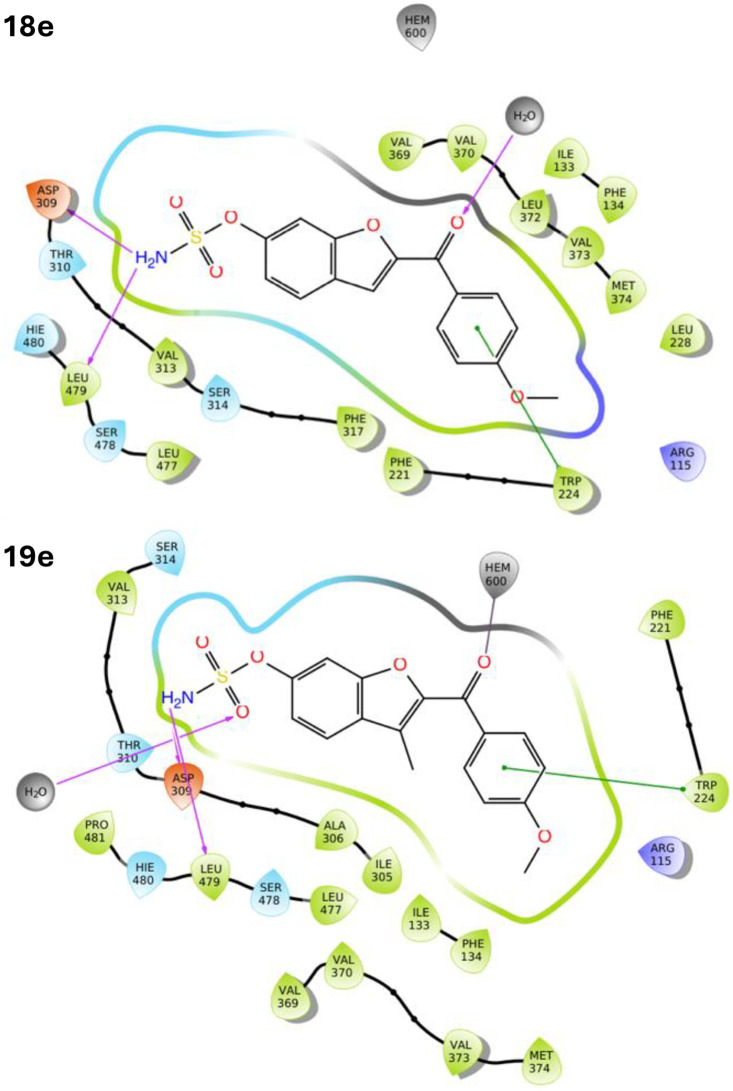
2D LigPlot figures of exemplar ketone sulfamates 18e and 19e within the CYP19A1 active site after MD simulation (150 ns), illustrating all amino acids within the CYP19A1 active site surrounding the sulfamate ligands.

The CYP19A1 protein–ligand complexes of the triazoles 6–10 and ketones 18–21 were generated by docking the triazoles and ketones with the X-ray crystal structure of CYP19A1 (pdb: 3S79)^28^ using the Molecular Operating Environment (MOE) programme^29^ as previously described^17^ and then subject to 150 ns molecular dynamics simulations using the Desmond programme of Schrödinger^30,31^ with exemplar compounds 6 and 10b used for the illustration of the triazole derivatives (Fig. 5) and exemplar compounds 18e and 19e used for the illustration of the ketone derivatives (Fig. 6).

The *R*-enantiomer of the triazole derivatives formed a Fe–N interaction *via* N2 of the triazole, while the *S*-enantiomers formed a preferable (perpendicular) Fe–N interaction *via* N4 of the triazole. The sulphonamide and carbamate moieties formed H-bonding interactions with two or three key amino acids, namely Asp309, Ser478 and His480 with van der Waals or π–π stacking interactions observed between the benzofuran ring/phenyl group and Trp224 (*R*-enantiomers) or Thr310 (*S*-enantiomers) and Phe221 (*R*-6 and *S*-10b). The nitrile group of the triazole derivative *R*/*S*-10b also formed a H-bonding interaction with Met374 (Fig. 5).

Generally, the ketone sulfamates 18 interacted indirectly (type I binding) with haem *via* a water molecule (*e.g.*18e, Fig. 6), while the 3-methyl derivatives 19 either formed a direct (type II) binding Fe–O interaction with the haem through the carbonyl group (*e.g.*19e, Fig. 6) or was positioned close to the haem. However, no haem interaction, direct or indirect, was observed for the carbamates 20 and 21. For all the ketone derivatives 18–21, the sulfamate and carbamate moieties formed the same H-bonding interactions observed with the triazole derivative 6–10, most commonly with Asp309, Ser478 and Leu479.

#### Steroid sulfatase (STS)

**Fig. 7:**
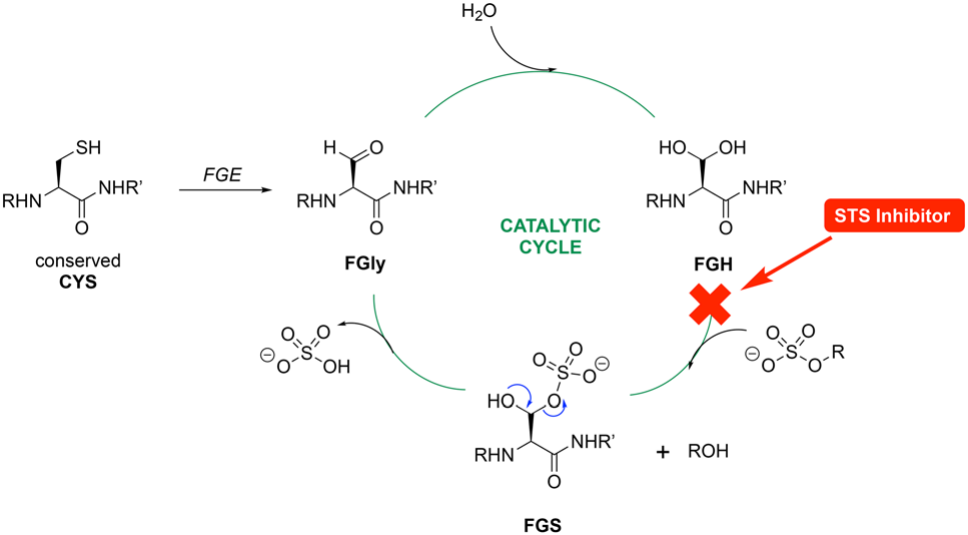
Steroid sulfatase (STS) catalytic cycle and point of STS inhibition.

In all eukaryote and prokaryote steroid sulfatase enzymes, a highly conserved cysteine active site residue within the steroid sulfatase motif is post-translationally oxidised into formylglycine (FGly) *via* a formylglycine generating enzyme (FGE).^32,33^ The hydroxylation of FGly to form the diol (FGH) is a required step for steroid sulfatase activity,^32–35^ with one of the hydroxy groups of the diol acting as a nucleophile during sulfate ester cleavage (Fig. 7).^36^ The STS sulfamate inhibitors interrupt the catalytic cycle at the FGH to the FGS stage by binding within the active site and specifically blocking further sulfation of FGH.

**Fig. 8:**
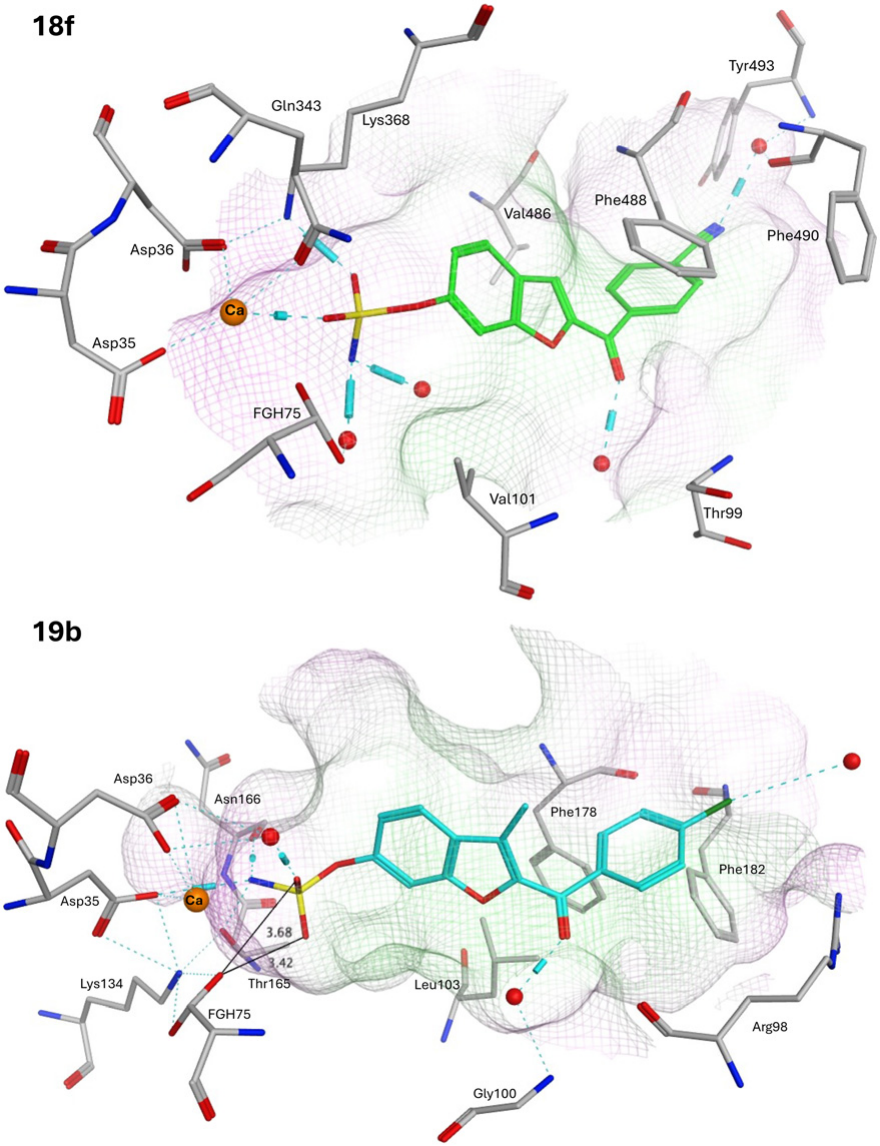
Exemplar ketone sulfamates 18f and 19b binding in the STS active site after MD simulation (150 ns). The Ca^2+^ cation is shown as an orange sphere, H_2_O molecules shown as red spheres, H-bonding cyan lines/barrels, and key binding amino acids in light grey. Pocket grid colours: green for lipophilic and pink for hydrophilic.

Computational studies for STS first involved modification of the FGS75 amino acid in the crystal structure of human placental oestrone sulfatase (pdb: 8EG3)^37^ to the diol (FGH) using the Builder tool in MOE. Waters and additional ligands were removed leaving the protein and Ca^2+^ and the resulting FGH-sulfatase protein prepared for docking to generate protein–ligand complexes. The protein–ligand complexes were then subject to 150 ns molecular dynamics simulations. The exemplar sulfamates 18f and 19b are used for the illustration of binding interactions (Fig. 8).

The benzofuran sulfamates 18 and 19 sit within the binding pocket, with the benzofuran moiety in the lipophilic domain, while the sulfamate moiety is positioned in the catalytic hydrophilic domain. In the catalytic domain, there are ten important residues: Asp35, Asp36, FGS75, Arg79, Lys134, His136, His290, Asp342, Lys368 and Gln343, with Asp35, Asp36, FGS75, Asp342 and Gln343 side chains forming a H-bonding network with the Ca^2+^ cation.^38^ In the protein–ligand complexes generated here, FGH75 has replaced FGS75 and using the exemplars 18f and 19b, the sulfamate moiety forms H-bonding interactions either directly or indirectly through H_2_O molecules with this network (Fig. 8), placing the sulfamate group in close proximity to the FGH diol. The ketone group is positioned to form a H_2_O-mediated interaction with the backbone NH of either Gly100 or Val101, and for 18f, the nitrile group is positioned to form a H_2_O-mediated interaction with the backbone NH of Thr493.

## Conclusions

The study with the triazole sulfamates accidently led to the preparation of additional carbamates, which showed potent Arom inhibitory activity (8b, IC_50_ 4.1 nM; 10b, IC_50_ 0.65 nM); however, neither the triazole sulfamates (6, 7, 8a, 9, 10a) nor the triazole carbamates (8b, 10b) displayed STS inhibitory activity; this can be explained by the reduced size of the STS active site compared with the Arom active site, which cannot accommodate the triazole ring. The superiority for the 6-position of the benzofuran for the sulfamate group over the 5-benzofuran or the 4-phenyl positions was also consistent with our previous findings for the alkylated 4th-generation Arom inhibitors.^17^ Replacing the CH-triazole with the ketone C=O resulted in the benzofuran ketone sulfamates 18 with good STS inhibitory activity but loss of the potent Arom inhibitory activity. A computational molecular dynamics study showed that generally, the ketone sulfamates 18 formed a type I (indirect) binding with haem (*e.g.*18e, Fig. 6), which would explain the reduction in Arom inhibitory activity. Introducing a methyl group in the 3-position of the furan in the benzofuran ring to give the methyl benzofuran ketone sulfamate derivatives 19 had a significant impact with dual Arom/STS inhibitory activity observed. Computational analysis of these DASIs would suggest that the introduction of the methyl group was sterically sufficient to move the compounds close enough to the haem to form a type II direct binding interaction (*e.g.*19e, Fig. 6), which would strengthen the binding translating to Arom inhibition, while the small steric increase was still accommodated within the STS active site retaining STS inhibitory activity. Further research is required to investigate optimal steric requirements with respect to DASI activity and to obtain a better understanding of structure–activity relationships.

## Experimental

### General

All commercially available starting materials and solvents were of general purpose or analytical grade and used without further purification. Solvents were appropriately dried over activated molecular sieves (4 Å). Melting points were determined using a Gallenkamp melting point apparatus and are uncorrected. ^1^H, ^19^F and ^13^C NMR (APT) spectra were recorded using a Bruker Advance DP500 spectrometer operating at 500, 400 and 125 MHz, respectively. Chemical shifts are given in parts per million (ppm) relative to the internal standard tetramethylsilane (Me_4_Si). Analytical thin-layer chromatography (TLC) was carried out on pre-coated silica plates (ALUGRAM® SIL G/UV254) with visualisation *via* UV light (254 nm). HPLC was performed either by the Department of Pharmacy & Pharmacology, University of Bath, Bath, UK using a Zorbax Eclipse plus C18 Rapid at a resolution of 2.1 × 50 mm a particle size of 1.8 μm using gradient (methanol : H_2_O) with 0.1% formic acid (method A) or in house using a Shimadzu LC-2030C Plus C18 Rapid at a resolution of 250 × 4.6 mm and a particle size of 5 μm using isocratic 80 : 20 (methanol : H_2_O) (method B). All biologically evaluated compounds are ≥95% pure by HPLC analysis or within 0.5% by elemental analysis. Phenolic compounds (1–5)^15–17^ and THP protected and phenolic derivatives (14 and 16)^16,17,22,23^ were prepared as described previously.

### Chemistry

#### General procedure for the preparation of triazole sulfamates 6–10

To a solution of phenolic compound (1–5) (1 equiv.) in dry CH_3_CN (10 mL mmol^−1^), K_2_CO_3_ (5.5 equiv.) was added and the mixture was stirred at 40 °C for 1 h, and then a freshly prepared solution of sulfamoyl chloride (5 equiv.) was added and the reaction mixture stirred at room temperature for 16 h. The reaction mixture was concentrated under reduced pressure and the residue dissolved in EtOAc (100 mL). The organic layer was washed with H_2_O (3 × 50 mL), dried (MgSO_4_) and concentrated under reduced pressure. Purification by gradient column chromatography afforded the required sulfamate at 80% EtOAc in petroleum ether (v/v).

#### 2-((4-Chlorophenyl)(1*H*-1,2,4-triazol-1-yl)methyl)benzofuran-6-yl sulfamate 6

(2-((4-Chlorophenyl)(1*H*-1,2,4-triazol-1-yl)methyl)benzofuran-6-ol (1) (0.14 g, 0.43 mmol) was used to prepare the product 6 as a colourless oil. Yield: 40 mg (23%), TLC: petroleum ether–EtOAc 1 : 1 v/v, *R*_f_ = 0.13. ^1^H NMR (DMSO-*d*_6_) *δ*: 8.77 (s, 1H, CH-triazole), 8.11 (s, 1H, CH-triazole), 7.99 (bs, 2H, NH_2_), 7.69 (d, *J* = 8.5 Hz, 1H, Ar), 7.54 (d, *J* = 8.6 Hz, 2H, Ar), 7.51 (d, *J* = 1.6 Hz, 1H, Ar), 7.49 (d, *J* = 8.6 Hz, 2H, Ar), 7.42 (s, 1H, CH), 7.21 (dd, *J* = 2.1, 8.5 Hz, 1H, Ar), 6.74 (s, 1H, Ar). ^13^C NMR (DMSO-*d*_6_) *δ*: 155.25 (C), 154.64 (C), 152.63 (CH), 148.17 (C), 144.94 (CH), 135.59 (C), 134.09 (C), 130.29 (2 × CH), 129.40 (2 × CH), 126.33 (C), 122.45 (CH), 118.79 (CH), 107.50 (CH), 106.35 (CH), 59.62(CH). HPLC (method B): 97.7% at R.T. = 3.99 min.

#### 2-((4-Chlorophenyl)(1*H*-1,2,4-triazol-1-yl)methyl)benzofuran-5-yl sulfamate 7

(2-((4-Chlorophenyl)(1*H*-1,2,4-triazol-1-yl)methyl)benzofuran-5-ol (2) (0.138 g, 0.43 mmol) was used to prepare the product 7 as a colourless oil. Yield: 50 mg (29%), TLC: petroleum ether–EtOAc 1 : 1 v/v, *R*_f_ = 0.13. ^1^H NMR (DMSO-*d*_6_) *δ*: 8.76 (s, 1H, CH-triazole), 8.11 (s, 1H, CH-triazole), 7.92 (bs, 2H, NH_2_), 7.65 (d, *J* = 8.9 Hz, 1H, Ar), 7.56 (d, *J* = 2.5 Hz, 1H, Ar), 7.54 (d, *J* = 8.6 Hz, 2H, Ar), 7.49 (d, *J* = 8.6 Hz, 2H, Ar), 7.43 (s, 1H, CH), 7.23 (dd, *J* = 2.5, 8.9 Hz, 1H, Ar), 6.76 (s, 1H, Ar). ^13^C NMR (DMSO-*d*_6_) *δ*: 155.62 (C), 153.04 (C), 152.65 (CH), 146.52 (C), 144.93 (CH), 135.55 (C), 134.09 (C), 130.31 (2 × CH), 129.40 (2 × CH), 128.58 (C), 120.25 (CH), 115.69 (CH), 112.53 (CH), 107.90 (CH), 59.65(CH). HPLC (method B): 97.5% at R.T. = 3.92 min.

#### 4-((5-Chlorobenzofuran-2-yl)(1*H*-1,2,4-triazol-1-yl)methyl)phenyl sulfamate 8a and carbamate 8b

4-((5-Chlorobenzofuran-2-yl)(1*H*-1,2,4-triazol-1-yl)methyl)phenol (3) (0.1 g, 0.3 mmol) was used to prepare the products 8a and 8b as white solids. Yield: sulfamate 8a 20 mg (16%) and carbamate 8b 10 mg (9%), m.p. sulfamate 8a 184–186 °C (16%) and carbamate 8b 68–70 °C. TLC: petroleum ether–EtOAc 1 : 3 v/v, *R*_f_ = 0.52 (carbamate) 0.35 (sulfamate). NMR 8a^1^H NMR (DMSO-*d*_6_) *δ*: 8.76 (s, 1H, CH-triazole), 8.10 (s, 1H, CH-triazole), 8.03 (bs, 2H, NH2), 7.76 (d, *J* = 2.3 Hz, 1H, Ar), 7.61 (d, *J* = 8.9 Hz, 1H, Ar), 7.57 (d, *J* = 8.6 Hz, 2H, Ar), 7.45 (s, 1H, CH), 7.34 (m, 3H, Ar), 6.71 (t, *J* = 0.9 Hz, 1H, Ar). ^13^C NMR (DMSO-*d*_6_) *δ*: 155.85 (C), 153.56 (C), 152.65 (CH), 150.81 (C), 144.90 (CH), 134.76 (C), 130.08 (2 × CH), 129.55 (C), 128.09 (C), 125.45 (CH), 123.17 (2 × CH), 121.56 (CH), 113.41 (CH), 107.22 (CH), 59.75(CH). NMR 8b^1^H NMR (DMSO-*d*_6_) *δ*: 8.70 (s, 1H, CH-triazole), 8.13 (s, 1H, CH-triazole), 7.95 (d, *J* = 2.1 Hz, 1H, Ar), 7.78 (d, *J* = 8.8 Hz, 1H, Ar), 7.65 (d, *J* = 8.7 Hz, 2H, Ar), 7.49 (bs, 1H, NH2), 7.01 (dd, *J* = 2.1, 8.8 Hz, 1H, CH), 6.97 (bs, 1H, NH_2_), 6.70 (d, *J* = 8.7 Hz, 2H, Ar), 6.45 (s, 1H), 6.67 (s, 1H, Ar). ^13^C NMR (DMSO-*d*_6_) *δ*: 154.32 (C), 153.94 (C), 153.68 (C), 152.54 (CH), 151.43 (C), 144.82 (CH), 132.38 (C), 129.05 (C), 128.92 (2 × CH), 128.77 (C), 125.62 (CH), 122.42 (2 × CH), 121.14 (CH), 112.59 (CH), 107.37 (CH), 61.55 (CH). HPLC 8a (method A) 100% at R.T. = 4.35 min. HPLC 8b (method A) 100% at R.T. = 4.33 min. HRMS 8a (EIC): calculated 404.0346 [M]^+^, found 404.0352 [M]^+^. HRMS 8b (EIC): calculated 368.0676 [M]^+^, found 368.0680 [M]^+^.

#### 2-((4-Fluorophenyl)(1*H*-1,2,4-triazol-1-yl)methyl)benzofuran-6-yl sulfamate 9

(2-((4-Fluorophenyl)(1*H*-1,2,4-triazol-1-yl)methyl)benzofuran-6-ol (4) (0.143 g, 0.46 mmol) was used to prepare the product 9 as a colourless oil. Yield: 70 mg (39%), TLC: petroleum ether–EtOAc 1 : 1 v/v, *R*_f_ = 0.15. ^1^H NMR (DMSO-*d*_6_) *δ*: 8.81 (s, 1H, CH-triazole), 8.16 (s, 1H, CH-triazole), 8.03 (bs, 2H, NH_2_), 7.75 (d, *J* = 8.5 Hz, 1H, Ar), 7.61 (dd, *J* = 5.4, 8.8 Hz, 2H, Ar), 7.56 (d, *J* = 1.8 Hz, 1H, Ar), 7.46 (s, 1H), 7.37 (t, *J* = 8.9 Hz, 2H, Ar), 7.26 (dd, *J* = 2.1, 8.5 Hz, 1H, Ar), 6.77 (t, *J* = 0.9 Hz, 1H, Ar). ^13^C NMR (DMSO-*d*_6_) *δ*: 163.64 (d, ^1^*J*_C,F_ = 243.75 Hz, C), 155.57 (C), 154.62 (C), 152.59 (CH), 148.13 (C), 144.85 (CH), 132.88 (d, ^4^*J*_C,F_ = 2.5 Hz, C), 130.74 (d, ^3^*J*_C,F_ = 7.5 Hz, 2 × CH), 126.35 (C), 122.42 (CH), 118.78 (CH), 116.35 (d, ^2^*J*_C,F_ = 22.5 Hz, 2 × CH), 107.33(CH), 106.34 (CH), 59.63 (CH). HPLC (method B): 100% at R.T. = 3.69 min.

#### 2-((4-Cyanophenyl)(1*H*-1,2,4-triazol-1-yl)methyl)benzofuran-6-yl sulfamate 10a and carbamate 10b

4-((6-Hydroxybenzofuran-2-yl)(1*H*-1,2,4-triazol-1-yl)methyl)benzonitrile (5) (0.209 g, 0.66 mmol) was used to prepare the product 10a as a colourless oil and the carbamate 10b as a white solid (m.p. 80–82 °C). Yield: sulfamate 10a 60 mg (23%) and carbamate 10b 24 mg (10%), TLC: petroleum ether–EtOAc 1 : 1 v/v, *R*_f_ = 0.22 (carbamate) 0.20 (sulfamate). NMR 10a^1^H NMR (DMSO-*d*_6_) *δ*: 8.79 (s, 1H, CH-triazole), 8.14 (s, 1H, CH-triazole), 7.99 (bs, 2H, NH2), 7.95 (d, *J* = 8.5 Hz, 2H, Ar), 7.70 (d, *J* = 8.5 Hz, 1H, Ar), 7.63 (d, *J* = 8.2 Hz, 2H, Ar), 7.56 (s, 1H, CH), 7.52 (d, *J* = 1.7 Hz, 1H, Ar), 7.22 (dd, *J* = 2.1, 8.5 Hz, 1H, Ar), 6.78 (t, *J* = 0.9 Hz, 1H, Ar). ^13^C NMR (DMSO-*d*_6_) *δ*: 154.68 (C), 154.52 (C), 152.79 (CH), 148.25 (C), 145.13 (CH), 141.73 (C), 133.38 (2 × CH), 129.29 (2 × CH), 126.25 (C), 122.54 (CH), 118.87 (CH), 112.18 (C), 107.89 (CH), 106.38 (CH), 60.22 (C), 59.75(CH). NMR 10b^1^H NMR (DMSO-*d*_6_) *δ*: 8.79 (s, 1H, CH-triazole), 8.13 (s, 1H, CH-triazole), 7.95 (d, *J* = 8.5 Hz, 2H, Ar), 7.62 (d, *J* = 8.2 Hz, 2H, Ar), 7.60 (d, *J* = 8.7 Hz, 1H, Ar), 7.52 (s, 1H, CH), 7.38 (d, *J* = 1.6 1H, Ar), 7.23 (bs, 1H, NH_2_), 7.03 (dd, *J* = 2.1, 8.5 Hz, 1H, Ar), 6.93 (bs, 1H, NH_2_), 6.71 (t, *J* = 1.0, 1H, Ar). ^13^C NMR (DMSO-*d*_6_) *δ*: 155.32 (C), 155.01 (C), 153.67 (C), 152.75 (CH), 149.45 (C), 145.13 (CH), 141.88 (C), 133.35 (2 × CH), 129.28 (2 × CH), 124.78 (C), 121.89 (CH), 118.88 (C), 118.70 (CH), 112.11 (C), 107.91 (CH), 106.09 (CH), 59.85 (CH). HPLC 10a (method A) 100% at R.T. = 3.94 min. HPLC 10b (method A) 100% at R.T. = 3.85 min. HRMS 10a (EI): calculated 396.0766 [M + H]^+^, found 396.0761 [M + H]^+^. HRMS 10b (EI): calculated 382.0916 [M + Na]^+^, found 382.0908 [M + Na]^+^.

#### 2-((4-Cyanophenyl)(1*H*-1,2,4-triazol-1-yl)methyl)benzofuran-6-yl carbamate 10b

To a solution of 4-((6-hydroxybenzofuran-2-yl)(1*H*-1,2,4-triazol-1-yl)methyl)benzonitrile (5) (0.1 g, 0.32 mmol) in dry CH_3_CN (5 mL), chlorosulfonyl isocyanate (0.14 mL, 1.58 mmol) was added and the reaction was stirred at room temperature for 3 h. The reaction mixture was then concentrated under reduced pressure and the residue dissolved in ice-water (10 mL) and stirred for 1 h. The mixture was extracted with EtOAc (100 mL), washed with H_2_O (2 × 100 mL), dried (MgSO_4_) and concentrated under reduced pressure to afford the product as a waxy white solid. Yield: 90 mg (81%). Analytical data are the same as described above.

#### General method for the synthesis of (3-methyl-6-((tetrahydro-2*H*-pyran-2-yl)oxy)benzofuran-2-yl)(phenyl)methanone derivatives 15 and 17d

To a solution of THP-protected acetophenone (12) (1 m eq.) in dry CH_3_CN (3 mL mmol^−1^ of 12), K_2_CO_3_ (2.2 m eq.) and 2-bromoacetophenone derivative (13) (1 m eq.) were added. The reaction mixture was stirred at 70 °C for 3 h. The solvent was then evaporated under reduced pressure and the residue dissolved in EtOAc (100 mL) and washed with H_2_O (3 × 50 mL). The organic layer was dried (MgSO_4_) and concentrated under reduced pressure to afford THP-protected benzofuran ketone derivatives (15).

#### (4-Fluorophenyl)(3-methyl-6-((tetrahydro-2*H*-pyran-2-yl)oxy)benzofuran-2-yl)methanone 15a

This compound was prepared using 1-(2-hydroxy-4-((tetrahydro-2*H*-pyran-2-yl)oxy)phenyl)ethan-1-one (12) (0.6 g, 2.54 mmol) and 2-bromo-4′-fluoroacetophenone (13a) (0.55 g, 2.54 mmol) and purified by recrystallisation from EtOH to afford the product as a pale orange crystalline solid. Yield: 0.52 g (58%), m.p.: 118–120 °C, TLC: petroleum ether–EtOAc 3 : 1 v/v, *R*_f_ = 0.65. ^1^H NMR (DMSO-*d*_6_) *δ*: 8.11 (m, 2H, Ar), 7.77 (d, *J* = 8.5 Hz, 1H, Ar), 7.43 (m, 2H, Ar), 7.32 (d, *J* = 2.0 Hz, 1H, Ar), 7.10 (dd, *J* = 2.0, 8.5 Hz, 1H, Ar), 5.62 (t, *J* = 3.5 Hz, 1H, CH-pyran), 3.79 (m, 1H, CH_2_-pyran), 3.61 (m, 1H, CH_2_-pyran), 2.56 (s, 3H, CH_3_), 1.93 (m, 3H, CH_2_-pyran), 1.68 (m, 3H, CH_2_-pyran). ^13^C NMR (DMSO-*d*_6_) *δ*: 183.43 (C), 166.05 (^1^*J*_C,F_ = 250 Hz, CF), 158.46 (C), 155.30 (C), 147.82 (C), 134.61 (^4^*J*_C,F_ = 3.13 Hz, CF), 132.70 (^2^*J*_C,F_ = 9.625 Hz, 2 × CH), 127.83 (C), 123.19 (C), 122.79 (CH), 116.11 (^3^*J*_C,F_ = 21.75 Hz 2 × CH), 115.52 (CH), 99.30 (CH), 96.57 (CH), 62.10 (CH_2_), 30.15 (CH_2_), 25.08 (CH_2_), 18.96 (CH_2_), 10.34 (CH_3_). ^19^F NMR (DMSO-*d*_6_) *δ*: −106.52.

#### (4-Chlorophenyl)(3-methyl-6-((tetrahydro-2*H*-pyran-2-yl)oxy)benzofuran-2-yl)methanone 15b

This compound was prepared using 1-(2-hydroxy-4-((tetrahydro-2*H*-pyran-2-yl)oxy)phenyl)ethan-1-one (12) (0.6 g, 2.54 mmol) and 2-bromo-1-(4-chlorophenyl)ethan-1-one (13b) (0.60 g, 2.54 mmol) and purified by two recrystallisation steps from EtOH to afford the product as a beige solid. Yield: 0.43 g (46%), m.p.: 108–110 °C, TLC: petroleum ether–EtOAc 3 : 1 v/v, *R*_f_ = 0.63. ^1^H NMR (DMSO-*d*_6_) *δ*: 8.03 (d, *J* = 9.0 Hz, 2H, Ar), 7.78 (d, *J* = 9.0 Hz, 1H, Ar), 7.67 (d, *J* = 8.4 Hz, 2H, Ar), 7.32 (d, *J* = 2.4 Hz, 1H, Ar), 7.11 (dd, *J* = 1.8, 8.4 Hz, 1H, Ar), 5.62 (t, *J* = 3.0 Hz, 1H, CH-pyran), 3.79 (m, 1H, CH_2_-pyran), 3.62 (m, 1H, CH_2_-pyran), 2.56 (s, 3H, CH_3_), 1.92 (m, 3H, CH_2_-pyran), 1.67 (m, 3H, CH_2_-pyran).^13^C NMR (DMSO-*d*_6_) *δ*: 183.68 (C), 158.55 (C), 155.35 (C), 147.74 (C), 137.92 (C), 136.70 (C), 131.57 (2 × CH), 129.08 (2 × CH), 128.12 (C), 123.17 (C), 122.86 (CH), 115.57 (CH), 99.27 (CH), 96.56 (CH), 62.10 (CH_2_), 30.14 (CH_2_), 25.07 (CH_2_), 18.95 (CH_2_), 10.36 (CH_3_).

#### (4-Bromophenyl)(3-methyl-6-((tetrahydro-2*H*-pyran-2-yl)oxy)benzofuran-2-yl)methanone 15c

This compound was prepared using 1-(2-hydroxy-4-((tetrahydro-2*H*-pyran-2-yl)oxy)phenyl)ethan-1-one (12) (0.6 g, 2.53 mmol) and 2-bromo-1-(4-bromophenyl)ethan-1-one (13c) (0.7 g, 2.53 mmol) and purified by gradient column chromatography eluting with petroleum ether–EtOAc 8 : 2 v/v to give the product as a yellow solid. Yield: 0.54 g (51%), m.p.: 96–98 °C, TLC: petroleum ether–EtOAc 3 : 1 v/v, *R*_f_ = 0.68. ^1^H NMR (DMSO-*d*_6_) *δ*: 7.94 (d, *J* = 9.0 Hz, 2H, Ar), 7.81 (d, *J* = 9.0 Hz, 2H, Ar), 7.78 (d, *J* = 8.4 Hz, 1H, Ar), 7.32 (d, *J* = 1.8 Hz, 1H, Ar), 7.11 (dd, *J* = 2.4, 9.0 Hz, 1H, Ar), 5.62 (t, *J* = 3.6 Hz, 1H, CH-pyran), 3.78 (m, 1H, CH_2_-pyran), 3.61 (m, 1H, CH_2_-pyran), 2.56 (s, 3H, CH_3_), 1.90 (m, 3H, CH_2_-pyran), 1.66 (m, 3H, CH_2_-pyran). ^13^C NMR (DMSO-*d*_6_) *δ*: 183.88 (C), 158.55 (C), 155.36 (C), 147.73 (C), 137.05 (C), 132.02 (2 × CH), 131.67 (2 × CH), 128.14 (C), 127.02 (C), 123.17 (C), 122.87 (CH), 115.58 (CH), 99.26 (CH), 96.57 (CH), 62.104 (CH_2_), 30.14 (CH_2_), 25.06 (CH_2_), 18.95 (CH_2_), 10.37 (CH_3_).

#### (4-Methoxyphenyl)(3-methyl-6-((tetrahydro-2*H*-pyran-2-yl)oxy)benzofuran-2-yl)methanone 15e

This compound was prepared using 1-(2-hydroxy-4-((tetrahydro-2*H*-pyran-2-yl)oxy)phenyl)ethan-1-one (12) (0.805 g, 3.407 mmol) and 2-bromo-4′-methoxyacetophenone (13e) (0.78 g, 3.407 mmol) and purified by gradient column chromatography eluting with petroleum ether–EtOAc 8 : 2 v/v and then recrystallisation from CH_3_CN to obtain the product as a pale yellow solid. Yield: 0.56 g (45%), m.p.: 88–90 °C, TLC: petroleum ether–EtOAc 3 : 1 v/v, *R*_f_ = 0.69. ^1^H NMR (DMSO-*d*_6_) *δ*: 8.05 (d, *J* = 9.0 Hz, 2H, Ar), 7.75 (d, *J* = 9.0 Hz, 1H, Ar), 7.33 (d, *J* = 2.4 Hz, 1H, Ar), 7.13 (d, *J* = 9.0 Hz, 2H, Ar), 7.10 (dd, *J* = 2.4, 9.0 Hz, 1H, Ar), 5.61 (t, *J* = 3.0 Hz, 1H, CH-pyran), 3.88 (s, 3H, O CH_3_), 3.80 (m, 1H, CH_2_-pyran), 3.62 (m, 1H, CH_2_-pyran), 2.54 (s, 3H, CH_3_), 1.92 (m, 2H, CH_2_-pyran), 1.79 (m, 1H, CH_2_-pyran), 1.66 (m, 3H, CH_2_-pyran). ^13^C NMR (DMSO-*d*_6_) *δ*: 183.39 (C), 163.33 (C), 158.16 (C), 155.07 (C), 148.26 (C), 132.22 (2 × CH), 130.50 (C), 126.70 (C), 122.60 (CH), 155.32 (CH), 114.30 (2 × CH), 99.32 (CH), 96.57 (CH), 62.10 (CH_2_), 56.01 (CH_2_), 30.17 (CH_2_), 25.08 (CH_2_), 19.0 (CH_2_), 10.30 (CH_3_).

#### 4-(3-Methyl-6-((tetrahydro-2*H*-pyran-2-yl)oxy)benzofuran-2-carbonyl)benzonitrile 15f

This compound was prepared as described using 1-(2-hydroxy-4-((tetrahydro-2*H*-pyran-2-yl)oxy)phenyl)ethan-1-one (12) (1 g, 4.23 mmol) and 2-bromo-4′-cyanoacetophenone (13f) (0.94 g, 4.23 mmol) and purified by gradient column chromatography eluting with petroleum ether–EtOAc 8 : 2 v/v and two hot washes in CH_3_CN to give the product as a yellow solid. Yield: 0.36 g (23%), m.p.: 198–200 °C, TLC: petroleum ether–EtOAc 3 : 1 v/v, *R*_f_ = 0.70. ^1^H NMR (CDCl_3_) *δ*: 8.20 (d, *J* = 8.4 Hz, 2H, Ar), 7.84 (d, *J* = 9.0 Hz, 2H, Ar), 7.62 (d, *J* = 7.6, 1H, Ar), 7.26 (d, *J* = 1.8 Hz, 1H, Ar), 7.11 (dd, *J* = 1.8, 8.4 Hz, 1H, Ar), 5.52 (t, *J* = 3.0 Hz, 1H, CH-pyran), 3.49 (m, 1H, CH_2_-pyran), 3.69 (m, 1H, CH_2_-pyran), 2.69 (s, 3H, CH_3_), 2.07 (m, 1H, Ar), 1.95 (m, 2H, CH_2_-pyran), 1.78 (m, 1H, CH_2_-pyran), 1.67 (m, 2H, CH_2_-pyran). ^13^C NMR (CDCl_3_) *δ*: 183.39 (C), 158.94 (C), 155.78 (C), 147.61 (C), 141.50 (2 × C), 132.06 (2 × CH), 130.06 (2 × CH), 129.55 (C), 123.20 (C), 121.98 (CH), 118.28 (C), 115.37 (CH), 98.82 (CH), 96.82 (CH), 62.14 (CH_2_), 30.22 (CH_2_), 25.08 (CH_2_), 18.61 (CH_2_), 10.28 (CH_3_).

#### (2,4-Dichlorophenyl)(6-hydroxy-3-methylbenzofuran-2-yl)methanone 17d

This compound was prepared using 1-(2-hydroxy-4-((tetrahydro-2*H*-pyran-2-yl)oxy)phenyl)ethan-1-one (12) (1.14 g, 4.82 mmol) and 2-bromo-2′,4′-dichloroacetophenone (13d) (1.29 g, 4.82 mmol) and purified by gradient column chromatography eluting with petroleum ether–EtOAc 8 : 2 v/v and two washes with CH_2_Cl_2_ to obtain (2,4-dichlorophenyl)(6-hydroxy-3-methylbenzofuran-2-yl)methanone 17d as a white solid. Yield: 0.70 g (45%), TLC: petroleum ether–EtOAc 2 : 1 v/v, *R*_f_ = 0.57. ^1^H NMR (DMSO-*d*_6_) *δ*: 10.32 (s, 1H, OH), 7.82 (d, *J* = 1.5 Hz, 1H, Ar), 7.67 (dd, *J* = 1.5, 7.5 Hz, 1H, Ar), 7.62 (s, 1H, Ar), 7.62 (d, *J* = 1.5, 1H, Ar), 6.9 (m, 2H, Ar), 2.37 (s, 3H, CH_3_).

#### General procedure for the synthesis of (6-hydroxy-3-methylbenzofuran-2-yl)(phenyl)methanones 17

To a solution of THP protected ketone (15) in dioxane (4.5 mL mmol^−1^), conc. HCl (1.15 mL mmol^−1^) was added and the reaction stirred at room temperature for 1 h. The reaction mixture was concentrated under reduced pressure and the residue washed with CH_2_Cl_2_ and then collected by vacuum filtration to afford 6-hydroxybenzofuran derivatives (17).

#### (4-Fluorophenyl)(6-hydroxy-3-methylbenzofuran-2-yl)methanone 17a

This compound was prepared using (4-fluorophenyl)(3-methyl-6-((tetrahydro-2*H*-pyran-2-yl)oxy)benzofuran-2yl)methanone (15a) (0.52 g,1.47 mmol) as a beige solid. Yield: 0.34 g (87%), m.p.: 214–216 °C, TLC: petroleum ether–EtOAc 3 : 1 v/v, *R*_f_ = 0.31. ^1^H NMR (DMSO-*d*_6_) *δ*: 10.19 (s, 1H, OH), 8.08 (m, 2H, Ar), 7.66 (d, *J* = 8.5 Hz, 1H, Ar), 7.42 (m, 2H, Ar), 6.95 (d, *J* = 2.0 Hz, 1H, Ar), 6.91 (dd, *J* = 2.0, 8.5 Hz, 1H, Ar), 2.53 (s, 3H, CH_3_). ^13^C NMR (DMSO-*d*_6_) *δ*: 183.19 (C), 165.94 (^1^*J*_C,F_ = 249.3 Hz, CF), 160.0 (C), 155.92 (C), 147.19 (C), 134.78 (^4^*J*_C,F_ = 2.63 Hz, CF), 132.59 (^3^*J*_C,F_ = 9.12 Hz, 2 × CH), 128.30 (C), 122.92 (CH), 121.40 (C), 116.06 (^2^*J*_C,F_ = 21.6 Hz, 2 × CH), 114.55 (CH), 97.86 (CH), 10.42 (CH_3_). ^19^F NMR (DMSO-*d*_6_) *δ*: −106.80.

#### (4-Chlorophenyl)(6-hydroxy-3-methylbenzofuran-2-yl)methanone 17b

This compound was prepared using (4-chlorophenyl)(3-methyl-6-((tetrahydro-2*H*-pyran-2-yl)oxy)benzofuran-2-yl)methanone (15b) (0.50 g, 1.33 mmol) as a yellow solid. Yield: 0.31 g (82%), m.p.: 190–192 °C, TLC: petroleum ether–EtOAc 4 : 1 v/v, *R*_f_ = 0.32. ^1^H NMR (DMSO-*d*_6_) *δ*: 10.21 (s, 1H, OH), 7.99 (d, *J* = 8.8 Hz, 2H, Ar), 7.64 (d, *J* = 8.8 Hz, 2H, Ar), overlapping 7.66 (d, *J* = 8.6 Hz, 1H, Ar), 6.94 (d, *J* = 2.0, Hz, 1H, Ar), 6.90 (dd, *J* = 2.0, 8.6 Hz, 1H, Ar), 2.53 (s, 3H, CH_3_). ^13^C NMR (DMSO-*d*_6_) *δ*: 183.42 (C), 160.11 (C), 155.98 (C), 147.13 (C), 137.73 (C), 136.91 (C), 131.50 (2 × CH), 129. 05 (2 × CH), 128.59 (C), 123.00 (CH), 121.40 (C), 114.62 (CH), 97.85 (CH), 10.43 (CH_3_).

#### (4-Bromophenyl)(6-hydroxy-3-methylbenzofuran-2-yl)methanone 17c

This compound was prepared using (4-bromophenyl)(3-methyl-6-((tetrahydro-2*H*-pyran-2-yl)oxy)benzofuran-2-yl)methanone (15b) (0.54 g, 1.3 mmol) as a green solid. Yield: 0.36 g (86%), m.p.: 218–220 °C, TLC: petroleum ether–EtOAc 3 : 1 v/v, *R*_f_ = 0.29. ^1^H NMR (DMSO-*d*_6_) *δ*: 10.21 (s, 1H, OH), 7.99 (d, *J* = 8.5 Hz, 2H, Ar), 7.67 (m, 3H, Ar), 6.95 (d, *J* = 2.0 Hz, 1H, Ar), 6.91 (dd, *J* = 2.0, 8.5 Hz, 1H, Ar), 2.53 (s, 3H, CH_3_). ^13^C NMR (CDCl_3_) *δ*: 183.62 (C), 160.14 (C), 156.00 (C), 147.12 (C), 137.28 (C), 132.00 (2 × CH), 131.62 (2 × CH), 128.62 (C), 126.83 (C), 123.02 (CH), 121.41 (C), 114.64 (CH), 97.86 (CH), 10.44 (CH_3_).

#### (6-Hydroxy-3-methylbenzofuran-2-yl)(4-methoxyphenyl)methanone 17e

This compound was prepared using (4-methoxyphenyl)(3-methyl-6-((tetrahydro-2*H*-pyran-2-yl)oxy)benzofuran-2-yl)methanone (15e) (0.94 g, 2.56 mmol) as a yellow solid. Yield: 0.26 g (37%), m.p.: 176–178 °C (lit m.p. = 177–178^39^), TLC: petroleum ether–EtOAc 2 : 1 v/v, *R*_f_ = 0.32. ^1^H NMR (DMSO-*d*_6_) *δ*: 10.15 (s, 1H, OH), 8.03 (d, *J* = 9.0 Hz, 2H, Ar), 7.64 (d, *J* = 8.4 Hz, 1H, Ar), 7.12 (d, *J* = 9.0 Hz, 2H, Ar), 6.96 (d, *J* = 2.4 Hz, 1H, Ar), 6.90 (dd, *J* = 1.8, 8.4 Hz, 1H, Ar), 3.84 (s, 3H, OCH_3_), 2.52 (s, 3H, CH_3_). ^13^C NMR (DMSO-*d*_6_) *δ*: 183.22 (C), 163.20 (C), 159.64 (C), 155.67 (C), 147.61 (C), 132.11 (2 × CH), 130.69 (C), 127.16 (C), 122.71 (CH), 121.44 (C), 114.33 (CH), 114.26 (2 × CH), 97.86 (CH), 55.98 (O CH_3_), 10.36 (CH_3_).

#### 4-(6-Hydroxy-3-methylbenzofuran-2-carbonyl)benzonitrile 17f

This compound was prepared using 4-(3-methyl-6-((tetrahydro-2*H*-pyran-2-yl)oxy)benzofuran-2-carbonyl) benzonitrile (15f) (0.49 g, 1.35 mmol) as a yellow solid. Yield: 0.34 g (92%), m.p.: 242 °C (sharp), TLC: petroleum ether–EtOAc 3 : 1 v/v, *R*_f_ = 0.45. ^1^H NMR (DMSO-*d*_6_) *δ*: 10.29 (s, 1H, OH), 8.09 (m, 2H, Ar), 8.05 (m, 2H, Ar), 7.69 (d, *J* = 8.5 Hz, 1H, Ar), 6.93 (d, *J* = 2.0 Hz, 1H, Ar), 6.91 (dd, *J* = 2.0, 8.5 Hz, 1H, Ar), 2.53 (s, 3H, CH_3_). ^13^C NMR (DMSO-*d*_6_) *δ*: 183.50 (C), 160.40 (C), 156.21 (C), 146.88 (C), 142.00 (C), 132.91 (2 × CH), 130.09 (2 × CH), 129.47 (C), 133.20 (CH), 121.38 (C), 118.75 (C), 114.81 (CH), 114.72 (C), 97.83 (CH), 10.46 (CH_3_).

#### General procedure for the preparation of ketone sulfamates 18–19

Method 1: as described for triazole derivatives with the following modifications: to a solution of ketone phenolic derivatives (16/17) in dry DMF, three equivalents of K_2_CO_3_ and three equivalents of commercially available sulfamoyl chloride were used. Method 2: to an ice-cold solution of ketone phenolic derivatives (16/17) in dry DMA, two equivalents of commercially available sulfamoyl chloride were added, and the reaction was stirred at 0 °C for 15 min and then at room temperature for 3 h. Cold brine (20 mL mmol^−1^ of 16/17) was added to the reaction mixture and then the product extracted with EtOAc (2 × 20 mL), dried (MgSO_4_) and concentrated under reduced pressure. The crude product was purified by gradient column chromatography.

#### 2-(4-Fluorobenzoyl)benzofuran-6-yl sulfamate 18a

This compound was prepared from (4-fluorophenyl)(6-hydroxybenzofuran-2-yl)methanone (16a) (0.22 g, 0.85 mmol) using method 1 and obtained as a white solid after purification by gradient column chromatography (petroleum ether–EtOAc 6 : 4 v/v). Yield: 0.22 g (76%), m.p.: 200–202 °C, TLC: petroleum ether–EtOAc 2 : 1 v/v, *R*_f_ = 0.37. ^1^H NMR (DMSO-*d*_6_) *δ*: 8 16 (s, 2H, NH2), 8.13 (m, 2H, Ar), 7.95 (dd, *J* = 8.5 Hz, 1H, Ar), 7.88 (dd, *J* = 1.0 Hz, 1H, Ar), 7.73 (s, 1H, CH-furan), 7.48 (m, 2H, Ar), 7.352 (dd, *J* = 2.0, 8.5 Hz, 1H, Ar). ^13^C NMR (DMSO-*d*_6_) *δ*: 182.23 (C), 166.51 (d, ^1^*J*_C,F_ = 251.25 Hz, C), 155.59 (C), 152.90 (C), 150.80 (C), 133.63 (d, ^4^*J*_C_ = 3.75 Hz, C), 132.75 (d, ^3^*J*_C_ = 8.75 Hz, 2 × CH), 125.67 (C), 125.02 (CH), 119.90 (CH), 117.30 (CH), 116.48 (d, ^2^*J*_C_ = 21.25 Hz, 2 × CH), 106.93 (CH). ^19^F NMR (DMSO-*d*_6_) *δ*: −105.60. Anal. calculated for C_15_H_10_FNO_5_S (335.3054): C, 53.73%; H, 3.01%; N, 4.18. Found: C, 53.63%; H, 3.07%; N, 3.96.

#### 2-(4-Chlorobenzoyl)benzofuran-6-yl sulfamate 18b

This compound was prepared from (4-chlorophenyl)(6-hydroxybenzofuran-2-yl)methanone (16b) (0.26 g, 0.96 mmol) using method 1 and obtained as a pale pink solid after purification by gradient column chromatography (petroleum ether–EtOAc 7 : 3 v/v). Yield: 61 mg (18%), m.p.: 159–161 °C, TLC: petroleum ether–EtOAc 2 : 1 v/v, *R*_f_ = 0.23. ^1^H NMR (CDCl_3_) *δ*: 8.15 (s, 2H, NH2), 8.04 (d, *J* = 8.5 Hz, 2H, H-2′ and H-6′), 7.94 (d, *J* = 8.6 Hz, 1H, H-4),7.89 (s, 1H, H-7), 7.73 (s, 1H, H-3), 7.70 (d, *J* = 8.5 Hz, 2H, H-3′ and H-5′), 7.34 (dd, *J* = 2.0, 8.6 Hz, 1H, H-5). ^13^C NMR (CDCl_3_) *δ*: 182.48 (C=O), 155.60 (C), 152.82 (C), 150.87 (C), 138.60 (C), 135.68 (C), 131.59 (CH-2′ and CH-6′), 129.38 (CH-3′ and CH-5′), 125.64 (C), 125.06 (CH), 119.92 (CH), 117.52 (CH), 106.93 (CH). Anal. calculated for C_15_H_10_ClNO_5_S (351.7570) C, 51.22%; H, 2.87%; N, 3.98%. Found: C, 51.70%; H, 3.01%; N, 3.72%.

#### 2-(4-Bromobenzoyl)benzofuran-6-yl sulfamate 18c

This compound was prepared from (4-bromophenyl)(6-hydroxybenzofuran-2-yl)methanone (16c) (0.22 g, 0.69 mmol) using method 1 and obtained as a white solid after purification by gradient column chromatography (petroleum ether–EtOAc 7 : 3 v/v). Yield: 0.125 g (46%), m.p.: 166–168 °C, TLC: petroleum ether–EtOAc 2 : 1 v/v, *R*_f_ = 0.33. ^1^H NMR (DMSO-*d*_6_) *δ*: 8. 15 (s, 2H, NH2), 7.97 (d, *J* = 8.4 Hz, 2H, Ar), 7.95 (d, *J* = 8.4 Hz, 1H, Ar), 7.90 (d, *J* = 1.2 Hz, 1H, Ar), 7.85 (d, *J* = 9.0 Hz, 2H, Ar), 7.73 (s, 1H, CH-furan), 7.36 (dd, *J* = 2.4, 9.0 Hz, 1H, Ar). ^13^C NMR (DMSO-*d*_6_) *δ*: 182.176 (C), 160.01 (C), 157.77 (C), 150.68 (C), 136.65 (C), 136.65 (2 × CH), 131.42 (2 × CH), 127.07 (C), 124.98 (CH), 119.49 (C), 119.04 (CH), 115.24 (CH), 97.93 (CH). Anal. calculated for C_15_H_10_BrNO_5_S·0.3 H_2_O (401.6156): C, 44.86%; H, 2.66%; N, 3.49%. Found: C, 44.92%; H, 2.59%; N, 3.09%. HPLC (method A) 100% at R.T. = 4.20 min. HRMS (EIC): calculated 397.9522 [M + H]^+^, found 397.9515 [M + H]^+^.

#### 2-(2,4-Dichlorobenzoyl)benzofuran-6-yl sulfamate 18d

This compound was prepared from (2,4-dichlorophenyl)(6-hydroxybenzofuran-2-yl)methanone (16d) (0.22 g, 0.85 mmol) using method 1 and obtained as a white solid after purification by gradient column chromatography (petroleum ether–EtOAc 7 : 3 v/v). Yield: 0.10 g (33%), m.p.: 198–200 °C, TLC: petroleum ether–EtOAc 2 : 1 v/v, *R*_f_ = 0.64. ^1^H NMR (DMSO-*d*_6_) *δ*: 8.16 (s, 2H, NH2), 7.91 (d, *J* = 8.5 Hz, 1H, Ar), 7.88 (s, 1H, Ar), 7.79 (d, *J* = 8.5 Hz, 1H, Ar), 7.73 (s, 1H, Ar), 7.71 (s, 1H, CH-furan), 7.67 (dd, *J* = 8.5 Hz, 1H, Ar), 7.34 (d, *J* = 9.0 Hz, 1H, Ar). ^13^C NMR (DMSO-*d*_6_) *δ*: 182.35 (C), 156.07 (C), 152.71 (C), 151.31 (C), 136.82 (C), 136.04 (C), 131.99 (C), 131.48 (CH), 130.23 (CH), 128.16 (CH), 125.59 (C), 125.35 (CH), 120.14 (CH), 119.07 (CH), 107.00 (CH). Anal. calculated for C_15_H_9_Cl_2_NO_5_S (386.199): C, 46.65%; H, 2.35%; N, 3.63. Found: C, 46.80%; H, 2.41%; N, 3.37.

#### 2-(4-Methoxybenzoyl)benzofuran-6-yl sulfamate 18e

This compound was prepared from (6-hydroxybenzofuran-2-yl)(4-methoxyphenyl)methanone (16e) (0.25 g, 0.93 mmol) using method 1 and obtained as a white solid after purification by gradient column chromatography (petroleum ether–EtOAc 7 : 3 v/v). Yield: 0.17 g (52%), m.p.: 182–184 °C, TLC: petroleum ether–EtOAc 2 : 1 v/v, *R*_f_ = 0.36. ^1^H NMR (DMSO-*d*_6_) *δ*: 8.14 (s, 2H, NH2), 8.08 (d, *J* = 9.0 Hz, 2H, Ar), 7.94 (d, *J* = 8.4 Hz, 1H, Ar), 7.82 (d, *J* = 1.2 Hz, 1H, Ar), 7.72 (s, 1H, CH-furan), 7.35 (dd, *J* = 2.4, 9.0 Hz, 1H, Ar), 7.17 (d, *J* = 9.0 Hz, 2H, Ar), 3.90 (s, 3H, OCH3). ^13^C NMR (DMSO-*d*_6_) *δ*: 181.74 (C), 163.42 (C), 159.54 (C), 157.41 (C), 151.26 (C), 131.84 (2 x CH), 130.04 (C), 124.67 (CH), 119.49 (C), 117.53 (CH), 114.94 (CH), 114.47 (2 x CH), 97.94 (CH), 56.04 (CH3). Anal. calculated for C_16_H_13_NO_6_S·0.2 H_2_O (350.9441): C, 54.76%; H, 3.84%; N, 3.99%. Found: C, 54.93%; H, 3.79%; N, 3.59%. HPLC (method A) 100% at R.T. = 4.39 min. HRMS (EIC): calculated 348.0543 [M + H]^+^, found 348.0534 [M + H]^+^.

#### 2-(4-Cyanobenzoyl)benzofuran-6-yl sulfamate 18f

This compound was prepared from 4-(6-hydroxybenzofuran-2-carbonyl)benzonitrile (16f) (0.28 g, 1.06 mmol) using method 2 and obtained as a white solid after purification by gradient column chromatography (petroleum ether–EtOAc 4 : 6 v/v). Yield: 0.27 g (74%), m.p.: 200–204 °C, TLC: petroleum ether–EtOAc 2 : 1 v/v, *R*_f_ = 0.38. ^1^H NMR (DMSO-*d*_6_): *δ* 8.18 (s, 2H, NH_2_), 8.14 (d, *J* = 8.6 Hz, 2H, Ar), 8.10 (d, *J* = 8.6 Hz, 2H, Ar), 7.95 (d, *J* = 8.6 Hz, 1H, Ar), 7.91 (d, *J* = 1.0 Hz, 1H, C*H*-furan), 7.73 (d, *J* = 1.6 Hz, 1H, Ar), 7.35 (dd, *J* = 2.0, 8.6 Hz, 1H, Ar). ^13^C NMR (DMSO-*d*_6_) *δ*: 182.69 (C), 155.77 (C), 152.53 (C), 151.07 (C), 140.67 (C), 133.20 (2 × CH), 130.26 (2 × CH), 125.62 (C), 125.25 (CH), 120.03 (CH), 118.61 (C), 118.36 (CH), 115.50 (C), 106.95 (CH). Anal. calculated for C_16_H_10_N_2_O_5_S·0.5 H_2_O (351.3326): C, 54.69%; H, 3.16%; N, 7.97%. Found: C, 54.73%; H, 3.21%; N, 7.53. HPLC (method A) 100% at R.T. = 4.06 min.

#### 2-(4-Fluorobenzoyl)-3-methylbenzofuran-6-yl sulfamate 19a

This compound was prepared from (4-fluorophenyl)(6-hydroxy-3-methylbenzofuran-2-yl)methanone (17a) (0.26 g, 0.96 mmol) using method 1 and obtained as a white solid after purification by gradient column chromatography (petroleum ether–EtOAc 7 : 3 v/v). Yield: 0.202 g (60%), m.p.: 181–183 °C, TLC: petroleum ether–EtOAc 2 : 1 v/v, *R*_f_ = 0.33. ^1^H NMR (DMSO-*d*_6_): *δ* 8.12 (m, 4H, H-2′, H-4′ and NH_2_), 7.97 (d, *J* = 8.6 Hz, 1H, H-4), 7.64 (d, *J* = 2.0 Hz, 1H, H-7), 7.44 (t, *J* = 8.9 Hz, 2H, H-3′ and H-5′), 7.34 (dd, *J* = 2.0, 8.6 Hz, 1H, H-5), 2.60 (s, 3H, CH_3_). ^13^C NMR (DMSO-*d*_6_): *δ* 183.80 (C=O), 166.29 (C, ^1^*J*_CF_ = 252 Hz), 153.86 (C), 150.99 (C), 148.95 (C), 134.23 (C, ^4^*J*_CF_ = 3.7 Hz), 132.87 (2 × CH, ^3^*J*_CF_ = 9.8 Hz), 127.40 (C), 127.00 (C), 123.15 (CH), 119.34 (CH-5), 116.19 (2 × CH, ^2^*J*_CF_ = 22.0 Hz), 106.77 (CH), 10.20 (CH_3_). ^19^F NMR (DMSO-*d*_6_): *δ* −105.9. Anal. calculated for C_16_H_12_FNO_5_S (349.3324) C, 55.01%; H, 3.46%; N, 4.01%. Found: C, 55.12%; H, 3.66%; N, 3.94%.

#### 2-(4-Chlorobenzoyl)-3-methylbenzofuran-6-yl sulfamate 19b

This compound was prepared from (4-chlorophenyl)(6-hydroxy-3-methylbenzofuran-2-yl)methanone (17b) (0.26 g, 0.906 mmol) using method 1 and obtained as a white solid after purification by gradient column chromatography (petroleum ether–EtOAc 7 : 3 v/v). Yield: 0.106 g (32%); m.p. 181–182 °C; TLC (petroleum ether–EtOAc 2 : 1 v/v) *R*_f_ 0.40. ^1^H NMR (DMSO-*d*_6_): *δ* 8.13 (s, 2H, NH_2_), 8.04 (d, *J* = 8.8 Hz, 2H, H-2′, and H-4′), 7.97 (dd, *J* = 0.5, 8.6 Hz, 1H, H-4), 7.68 (d, *J* = 8.8 Hz, 2H, H-3′ and H-5′), 7.64 (dd, *J* = 0.5, 2.0 Hz, 1H, H-5), 7.34 (dd, *J* = 2.0, 8.6 Hz, 1H, H-7), 2.60 (s, 3H, CH_3_). ^13^C NMR (DMSO-*d*_6_): *δ* 184.07 (C=O), 153.90 (C), 151.07 (C), 148.87 (C), 138.35 (C), 136.32 (C), 131.72 (2 × CH), 129.22 (2 × CH), 127.38 (C), 127.27 (C), 123.21 (CH), 119.38 (CH), 106.77 (CH), 10.22 (CH_3_). Anal. calculated for C_16_H_12_ClO_5_NS (365.7840) C, 52.54%; H, 3.31%; N, 3.83%. Found: C, 52.75%; H, 3.32%; N, 3.64%.

#### 2-(4-Bromobenzoyl)-3-methylbenzofuran-6-yl sulfamate 19c

This compound was prepared from (4-bromophenyl)(6-hydroxy-3-methylbenzofuran-2-yl)methanone (17c) (0.30 g, 0.907 mmol) using method 1 and obtained as a pale yellow solid after purification by gradient column chromatography (petroleum ether–EtOAc 7 : 3 v/v). Yield: 0.093 g (25%); m.p. 178–181 °C; TLC (petroleum ether–EtOAc 2 : 1 v/v) *R*_f_ 0.30. ^1^H NMR (CDCl_3_) *δ*: 8.13 (s, 2H, NH_2_), 7.96 (m, 3H, H-2′, H-6′ and H-4), 7.83 (d, *J* = 8.6 Hz, 2H, H-3′ and H-5′), 7.64 (d, *J* = 2.0 Hz, 1H, H-7), 7.34 (dd, *J* = 2.1, 8.6 Hz, 1H, H-5), 2.51 (s, 3H, CH_3_). ^13^C NMR (CDCl_3_) *δ*: 184.27 (C=O), 153.90 (C), 151.07 (C), 148.86 (C), 136.66 (C), 132.16 (2 × CH), 131.80 (2 × CH), 127.48 (C), 127.38 (C), 127.28 (C), 123.22 (CH), 119.38 (CH), 106.77 (CH), 10.23 (CH_3_). Anal. calculated for C_16_H_12_BrO_5_NS (410.2380) C, 46.84%; H, 2.95%; N, 3.41%. Found: C, 47.13%; H, 3.25%; N, 3.31%.

#### 2-(2,4-Dichlorobenzoyl)-3-methylbenzofuran-6-yl sulfamate 19d

This compound was prepared from (2,4-dichlorophenyl)(6-hydroxy-3-methylbenzofuran-2-yl)methanone (17d) (0.4 g, 1.24 mmol) using method 2 and obtained as a white solid after purification by gradient column chromatography (petroleum ether–EtOAc 6 : 4 v/v). Yield: 0.107 g (22%); m.p. 138–140 °C; TLC (petroleum ether–EtOAc 2 : 1 v/v) *R*_f_ 0.42. ^1^H NMR (DMSO-*d*_6_): *δ* 8.13 (s, 2H, NH_2_), 7.98 (d, *J* = 8.6 Hz, 1H, Ar), 7.86 (d, *J* = 1.9 Hz, 1H, Ar), 7.69 (d, *J* = 8.3 Hz, 1H, Ar), 7.65 (dd, *J* = 2.0, 8.3 Hz, 1H, Ar), 7.58 (d, *J* = 2.0 Hz, 1H, Ar), 7.33 (dd, *J* = 2.0, 8.6 Hz, 1H, Ar), 2.49 (s, 3H, CH_3_). ^13^C NMR (DMSO-*d*_6_) *δ*: 184.21 (C=O), 154.34 (C), 151.51 (C), 148.35 (C), 137.20 (C), 136.58 (C), 131.62 (C), 131.05 (CH), 130.00 (CH), 128.41 (CH), 127.84 (C), 127.45 (C), 123.69 (CH), 119.54 (CH), 106.74 (CH), 9.85 (CH_3_). Anal. calculated for C_16_H_11_Cl_2_O_5_NS (400.2260) C, 48.02%; H, 2.77%; N, 3.50%. Found: C, 47.91%; H, 2.82%; N, 3.31%.

#### 2-(4-Methoxybenzoyl)-3-methylbenzofuran-6-yl sulfamate 19e

This compound was prepared from (6-hydroxy-3-methylbenzofuran-2-yl)(4-methoxyphenyl)methanone (17e) (0.19 g, 0.67 mmol) using method 2 and obtained as a white solid after purification by gradient column chromatography (petroleum ether–EtOAc 6 : 4 v/v). Yield: 0.138 g (56%); m.p. 172–176 °C; TLC (petroleum ether–EtOAc 2 : 1 v/v) *R*_f_ 0.22. ^1^H NMR (DMSO-*d*_6_): *δ* 8.13 (s, 2H, NH_2_), 8.06 (d, *J* = 8.9 Hz, 2H, Ar), 7.94 (d, *J* = 8.6 Hz, 1H, Ar), 7.64 (d, *J* = 2.0 Hz, 1H, Ar), 7.33 (dd, *J* = 2.0, 8.6 Hz, 1H, Ar), 7.14 (d, *J* = 8.9 Hz, 2H, Ar), 3.89 (s, 3H, OCH_3_), 2.49 (s, 3H, CH_3_). ^13^C NMR (DMSO-*d*_6_): *δ* 183.64 (C=O), 163.65 (C), 153.66 (C), 150.68 (C), 149.42 (C), 132.40 (2 × CH), 130.12 (C), 127.49 (C), 125.87 (C), 122.93 (CH), 119.24 (CH), 114.45 (2 × CH), 106.76 (CH), 56.08 (OCH_3_), 10.14 (CH_3_). Anal. calculated for C_17_H_15_O_6_NS·0.1 H_2_O (363.1695) C, 56.22%; H, 4.22%; N, 3.86%. Found: C, 56.07%; H, 4.20%; N, 3.75%. HPLC (method A) 100% at R.T. = 4.35 min. HRMS (EIC): calculated 362.0699 [M + H]^+^, found 362.0688 [M + H]^+^.

#### 2-(4-Chlorobenzoyl)benzofuran-6-yl carbamate 20

To a suspension of (4-chlorophenyl)(6-hydroxybenzofuran-2-yl)methanone (16b) (0.19 g, 0.7 mmol) in dry CH_3_CN (11 mL), chlorosulfonyl isocyanate (0.3 mL, 3.5 mmol) was added, and the resulting orange-brown solution was stirred at room temperature for 90 min. The solvent was then removed *in vacuo* and ice–H_2_O (30 mL) was added. The initial residue was dissolved to form a pale yellow solution, which was stirred at room temperature for 1 h. The reaction mixture was extracted with EtOAc (100 mL) and the organic layer was washed with H_2_O (2 × 100 mL), dried (MgSO_4_) and evaporated under reduced pressure. The resulting residue was recrystallised from CH_3_CN to obtain a pale pink solid. Yield: 0.128 g (58%), m.p.: 190–192 °C, TLC (petroleum ether–EtOAc 3 : 1 v/v) *R*_f_ 0.26. ^1^H NMR (DMSO-*d*_6_) *δ*: 8.02 (d, *J* = 8.3 Hz, 2H, H-2′ and H-6′), 7.84 (m, 2H, H-4 and H-7), 7.68 (d, *J* = 8.6 Hz, 2H, H-3′ and H-5′), 7.60 (s, 1H, H-3), 7.36 (br s, 1H of NH_2_), 7.17 (dd, *J* = 2.0, 8.6 Hz, 1H, H-5), 7.05 (br s, 1H of NH_2_). ^13^C NMR (DMSO-*d*_6_) *δ*: 182.51 (C=O, C-1), 155.99 (C), 154.95 (C), 152.28 (C), 152.26 (C), 138.43 (C), 135.87 (C), 131.53 (2 × CH, Ar), 129.34 (2 × CH, Ar), 127.37 (CH), 124.26 (C), 120.01 (CH), 117.88 (CH), 106.53 (CH). Anal. calculated for C_16_H_10_ClNO_4_ (315.7090): C, 60.87%; H, 3.19%; N, 4.43. Found: C, 61.03%; H, 3.27%; N, 4.01.

#### 2-(4-Chlorobenzoyl)-3-methylbenzofuran-6-yl carbamate 21

This compound was prepared following the same procedure as described for 20 using (4-chlorophenyl)(6-hydroxy-3-methylbenzofuran-2-yl)methanone (17b) (0.13 g, 0.45 mmol) and obtained as a light yellow solid after recrystallisation from CH_3_CN. Yield: 93 mg (63%), m.p.: 210–212 °C, TLC (petroleum ether–EtOAc 4 : 1 v/v) *R*_f_ 0.12. ^1^H NMR (DMSO-*d*_6_) *δ*: 8.02 (d, *J* = 8.7 Hz, 2H, H-2′ and H-6′), 7.86 (d, *J* = 8.6 Hz, 1H, H-4), 7.66 (d, *J* = 8.7 Hz, 2H, H-3′ and H-5′), 7.51 (d, *J* = 1.9 Hz, 1H, H-7), 7.35 (br s, 1H of NH_2_), 7.17 (dd, *J* = 2.0, 8.6 Hz, 1H, H-5), 7.05 (br s, 1H of NH_2_), 2.58 (s, 3H, CH_3_). ^13^C NMR (DMSO-*d*_6_) *δ*: 184.10 (C=O, C-1), 154.96 (C), 154.24 (C), 152.43 (C), 148.41 (C), 138.17 (C), 136.49 (C), 131.68 (2 × CH, Ar), 129.15 (2 × CH, Ar), 127.47 (C), 126.04 (C), 122.49 (CH), 119.41 (CH), 106.44 (CH), 10.28 (CH_3_). Anal. calculated for C_17_H_12_ClNO_4_ (329.7360): C, 61.92%; H, 3.67%; N, 4.25%. Found: C, 61.57%; H, 3.79%; N, 3.96%.

### Cell culture

JEG-3 cells (ATCC) were cultured in Eagle's minimal essential medium (EMEM) supplemented with 10% fetal calf serum (FCS). The cells were maintained at 37 °C in a humidified incubator with 5% CO_2_. Then, the cells were cultured to approximately 80% confluence before any treatments were applied.

### Aromatase activity assay

The aromatase activity was measured using a modified tritiated water assay. The JEG-3 cells were cultured in six-well plates in EMEM until reaching 80% confluence. The substrate androst-4-ene-3,17-dione [1β-^3^H] was added to each well in a serum-free medium. In the presence and absence of dual inhibitors (at concentrations ranging from 0.001 to 10 nM), the cells were incubated at 37 °C for 1 hour. The reaction was stopped by placing the plates on ice for 5 minutes. The medium (500 μL) was obtained, vortexed with 2% dextran-treated charcoal in PBS, and centrifuged at 4000 rpm. [^3^H]H_2_O in the supernatant was quantified using scintillation counting. The aromatase activity was normalized to protein concentration, determined by the Pierce BCA assay kit (Thermo Fisher Scientific), and expressed as pmol of product per mg of protein per hour.

### Steroid sulfatase activity assay

STS activity was measured using an *in vitro*, cell-free assay. Briefly, lysates from JEG-3 cells, known to have high endogenous STS activity, were prepared by lysing the cells with a buffer (50 mM Tris-HCl, pH 7.4, and 1 mM EDTA). After centrifugation, the supernatant was collected and protein concentrations were determined using a BCA assay kit.

For the steroid sulfatase assay, 125 μg of JEG-3 lysate was incubated with the substrate [^3^H]oestrone sulfate (E1S, 4 × 10^5^ dpm) diluted to a final concentration of 20 μM using non-radioactive E1S. The mixture was incubated in the presence of different concentrations of inhibitors (ranging from 10^−11^ M to 10^−5^ M) at 37 °C for 1 hour. After incubation, the reaction was stopped by placing the tubes on ice, and the estrone product formed was separated from the substrate by extraction with toluene. [^14^C]oestrone was added to the organic phase as an internal standard to monitor recovery and procedural losses.

The organic phase was subjected to liquid scintillation counting to measure both ^3^H and ^14^C. The amount of E1S hydrolysed was calculated from the ^3^H counts, corrected for recovery using the ^14^C standard, and converted into molar concentrations of hydrolysed product. The inhibitory activity of test compounds was expressed as the IC_50_ value, representing the concentration required to inhibit 50% of the STS activity.

## Data availability

The data supporting this article have been included as part of the ESI.†

## Author contributions

AGE, FG, OA, IS and HA-B performed the chemical synthesis supervised by CS. CEP and ND performed the enzyme assays supervised by PAF. CS performed the computational studies. The manuscript was prepared by CS and PAF and reviewed by all authors.

## Conflicts of interest

There are no conflicts to declare.

## Supplementary Material

MD-OLF-D4MD00795F-s001
