# Supplementary material for: Development of benzofuran-derived sulfamates as dual aromatase-steroid sulfatase inhibitors (DASIs): design, synthesis and biological evaluation
Source: RSC Med Chem. 2025 Jan 9;16(4):1606–18. doi: 10.1039/d4md00795f (PMC11792066; doi:10.1039/d4md00795f)

Supplementary Information (SI) for RSC Medicinal Chemistry.
This journal is © The Royal Society of Chemistry 2025

SUPPORTING INFORMATION

Development of benzofuran derived sulfamates as dual aromatase-steroid sulfatase inhibitors (DASIs): Design, synthesis and biological evaluation

Ahmed G. Eissa, Francesca Gozzi, Oqab Aloqab, Charlotte E. Parrish, Nadira Mohamed, Irene Shiali, Harith Al-Baldawi, Paul A. Foster, Claire Simons

**Figure S1**. Comparison between the ^{1}H NMR spectra of the sulfamate (**10a**) and carbamate (**10b**) products indicating the difference in the amino group signals.	S2
^{1}H, ^{13}C, ^{19}F NMR and HPLC of final compounds	S3-S29

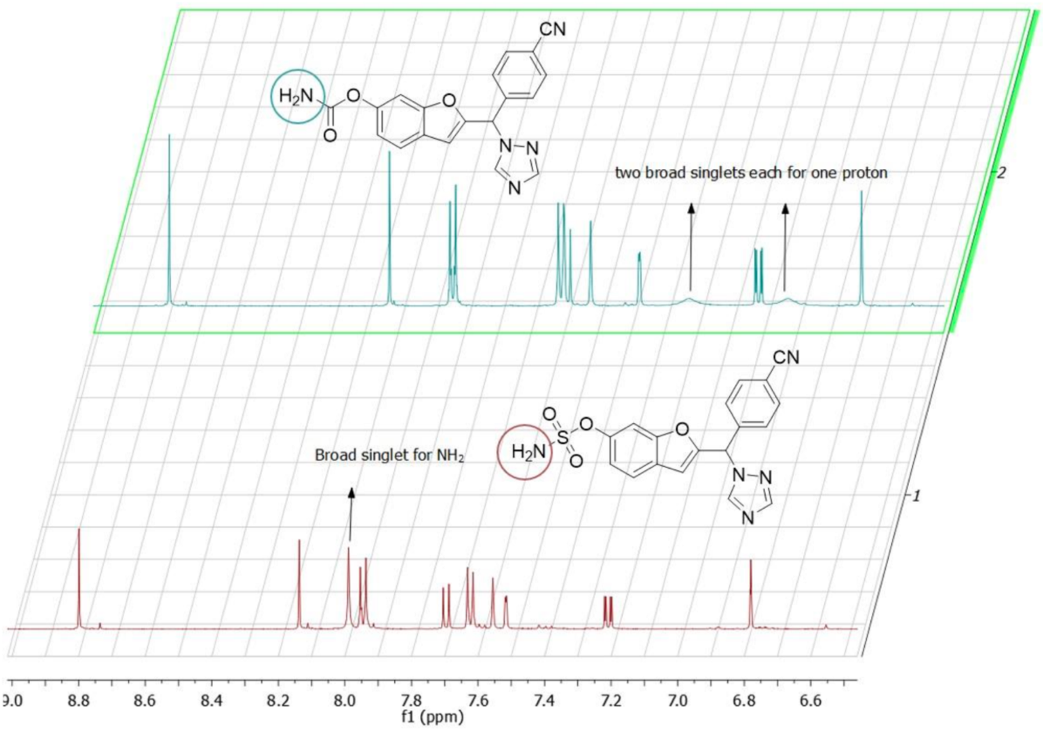

Figure S1. Comparison between the ^{1}H NMR spectra of the sulfamate (**10a**) and carbamate (**10b**) products indicating the difference in the amino group signals.

Compound **6**

^{1}H NMR (DMSO-d_6)

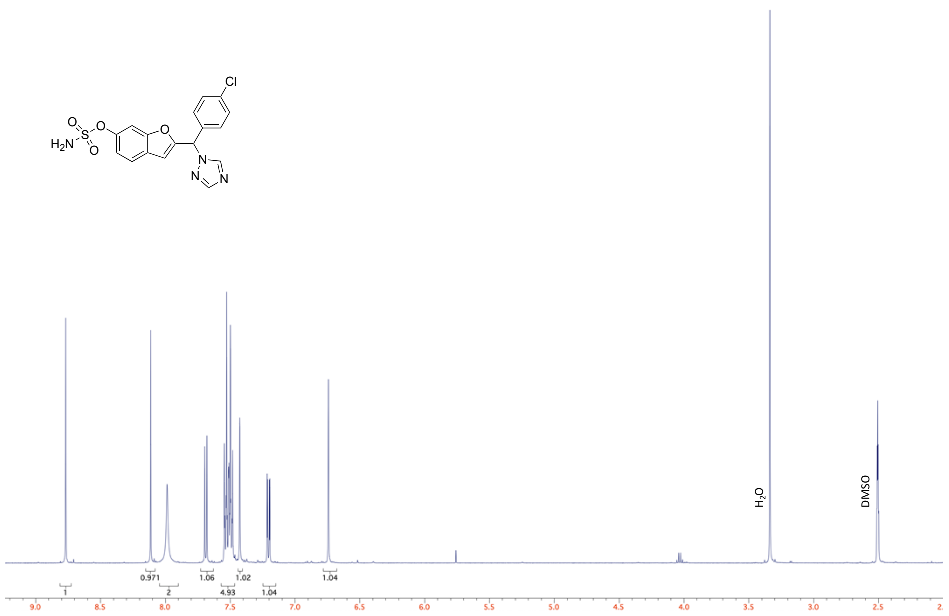

^{13}C NMR (DMSO-d_6)

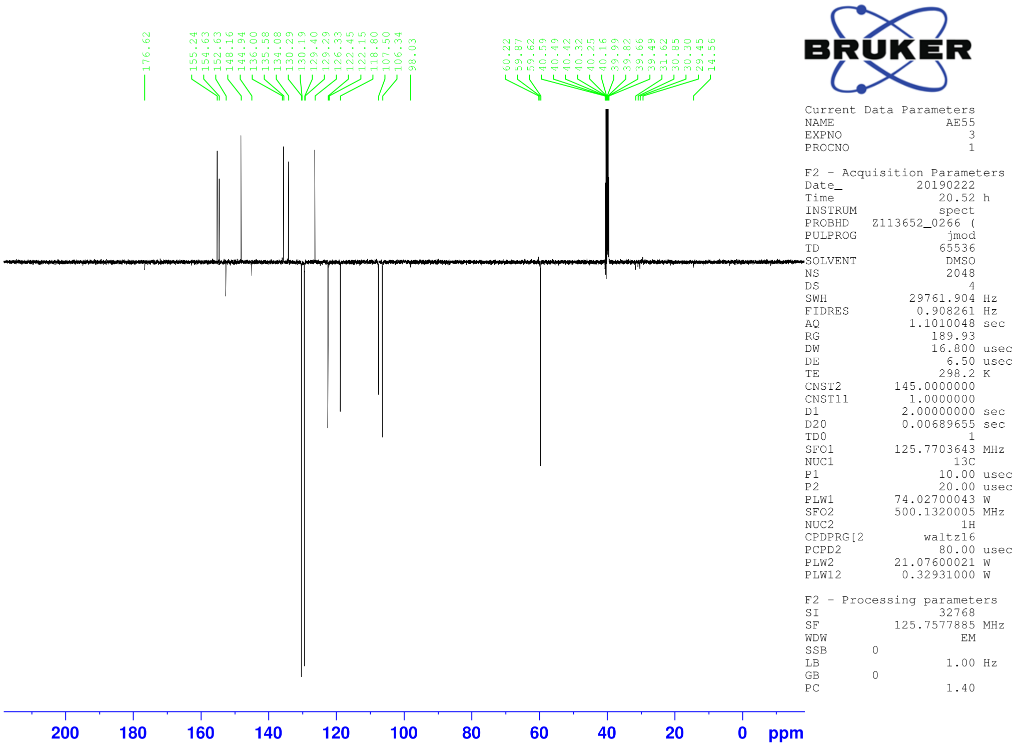

HPLC

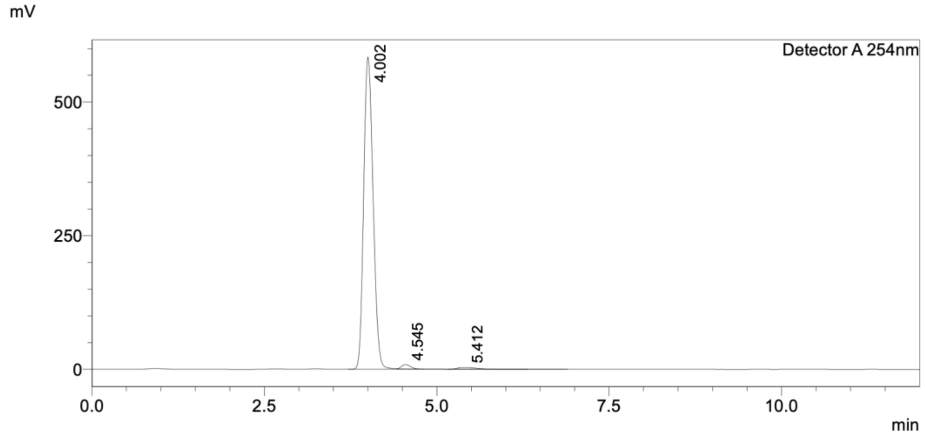

<Peak Table>

Detector A 254nm

Peak#	Ret. Time	Area	Height	Conc.	Area%
1	4.002	5616899	583925	97.697	97.697
2	4.545	68644	7868	1.194	1.194
3	5.412	63752	2997	1.109	1.109
Total		5749295	594791		100.000

Compound **7**

^{1}H NMR (DMSO-d_6)

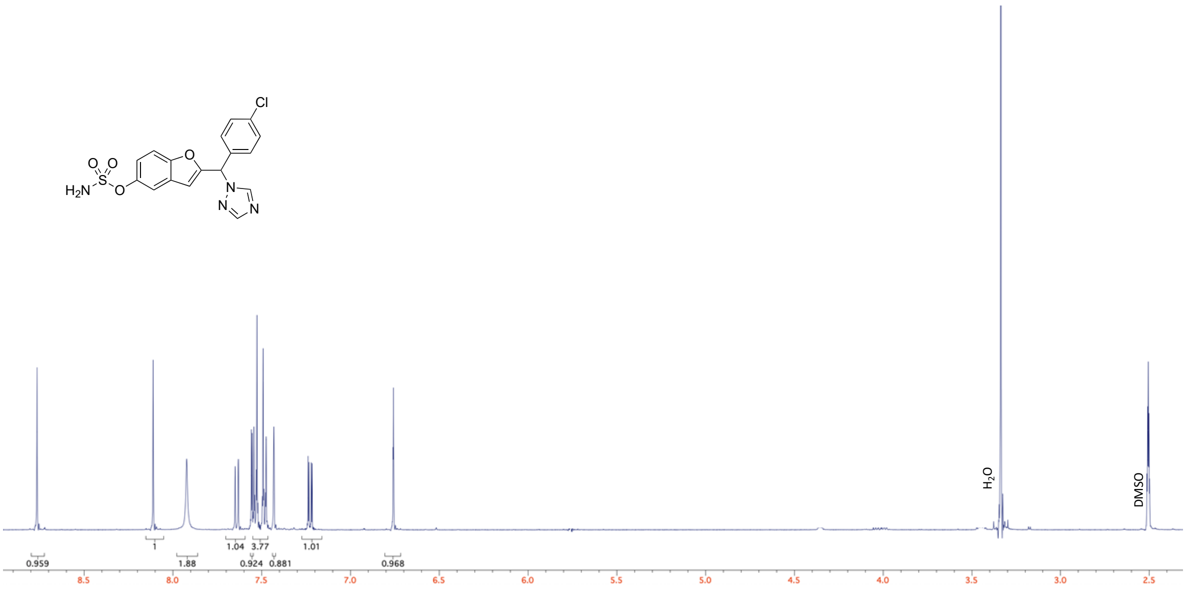

^{13}C NMR (DMSO-d_6)

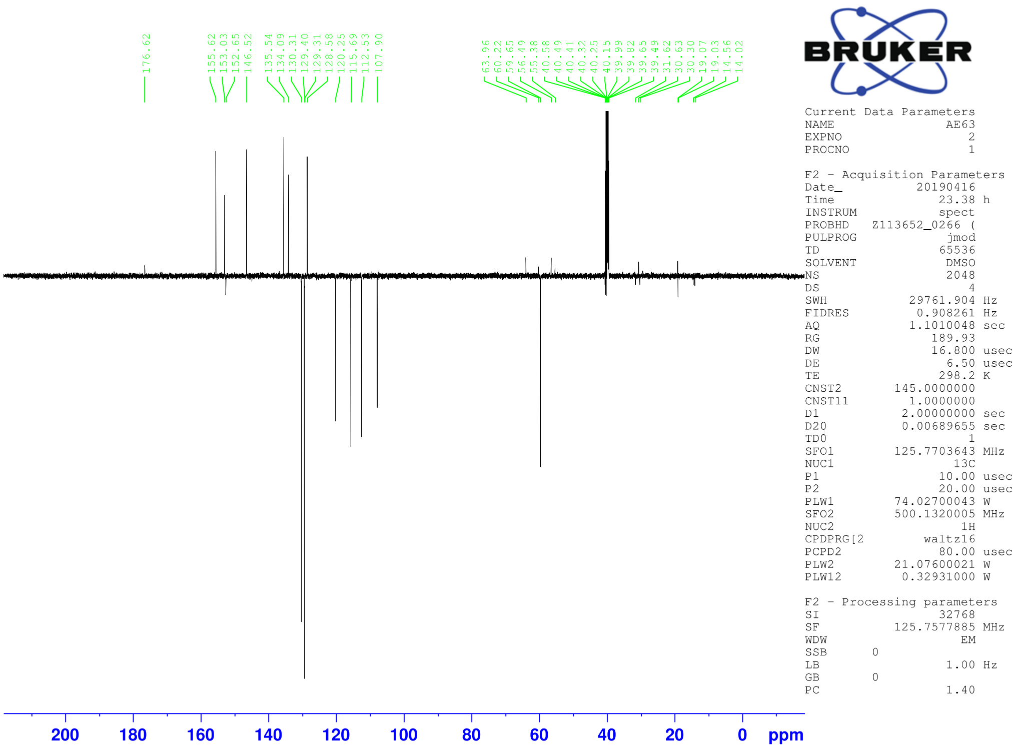

HPLC data

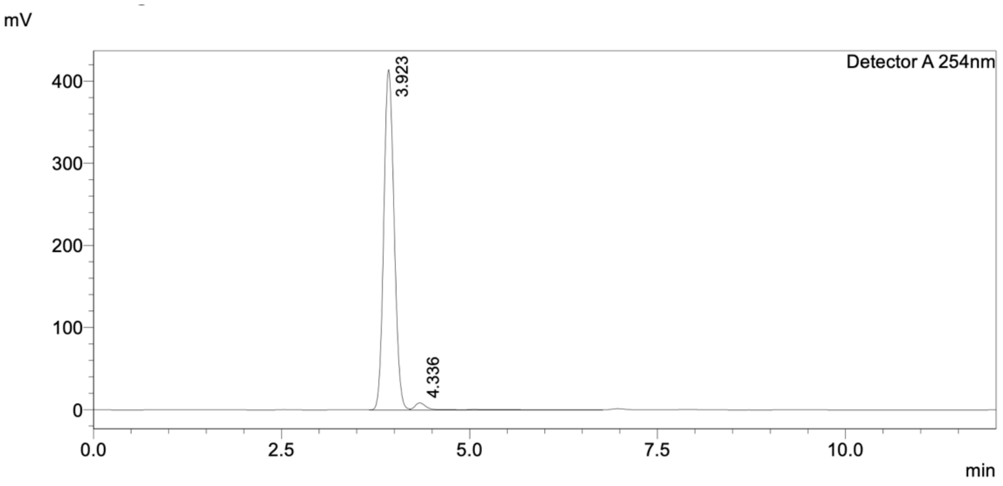

<Peak Table>

Detector A 254nm

Peak#	Ret. Time	Area	Height	Conc.	Area%
1	3.923	3974368	414070	97.481	97.481
2	4.336	102705	8574	2.519	2.519
Total		4077072	422644		100.000

Compound **8a**

^{1}H NMR (DMSO-d_6)

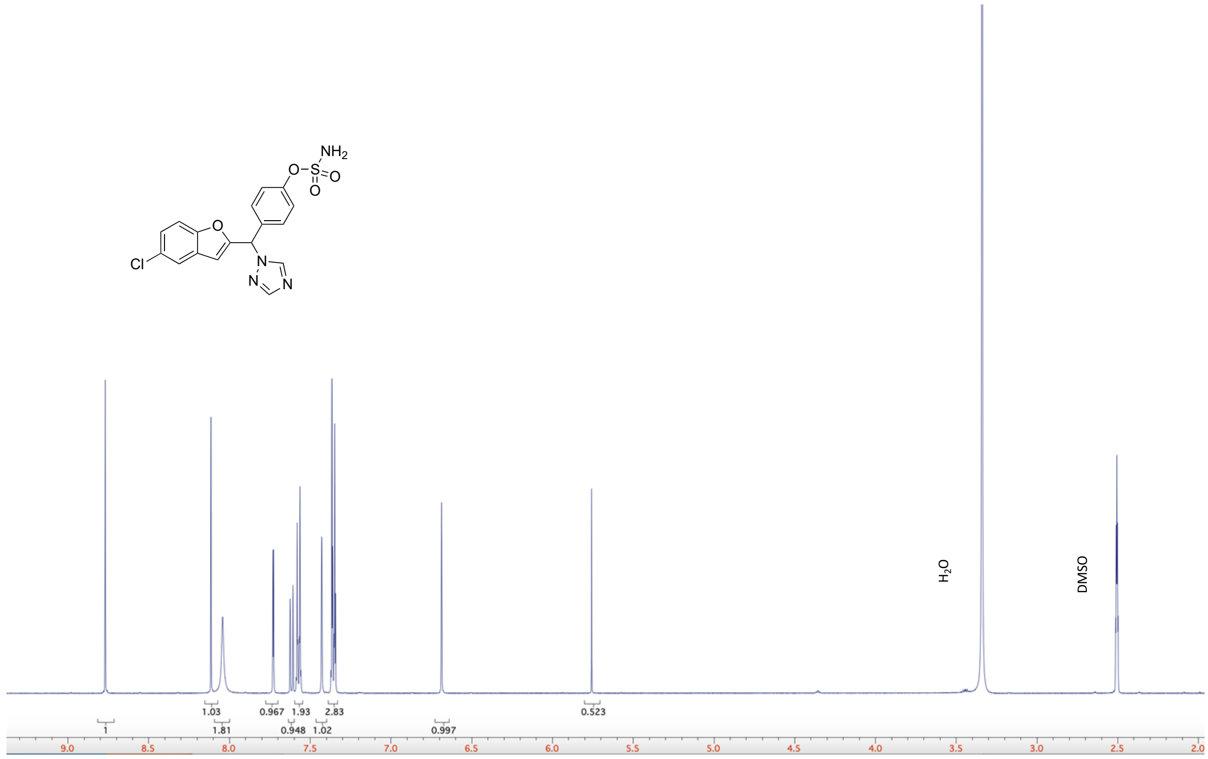

^{13}C NMR (DMSO-d_6)

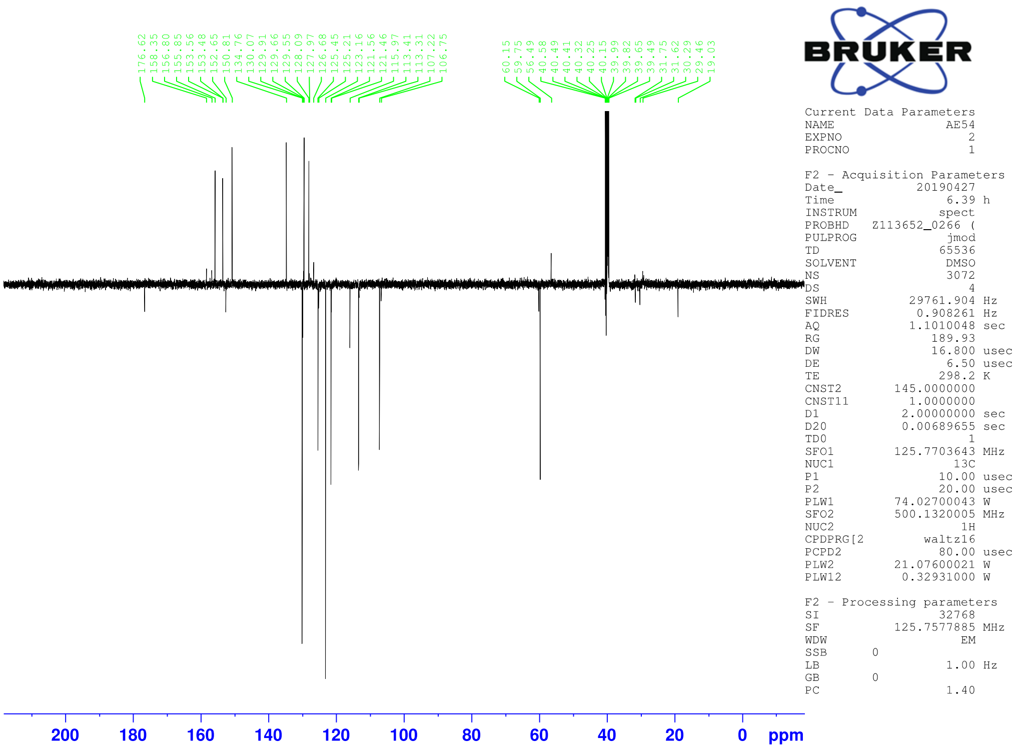

HPLC

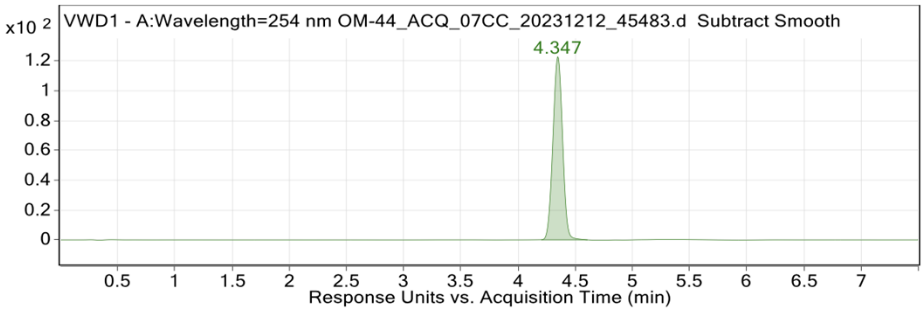

Figure: Base peak or HPLC chromatogram (indicated in left hand corner)

User Chromatogram Peak List

RT (min)	Area	Area %	Area Sum (%)	Symmetry	Width (min)
4.35	722.42	100.00	100.00	1.03	0.402

Compound **8b**

^{1}H NMR (DMSO-d_6)

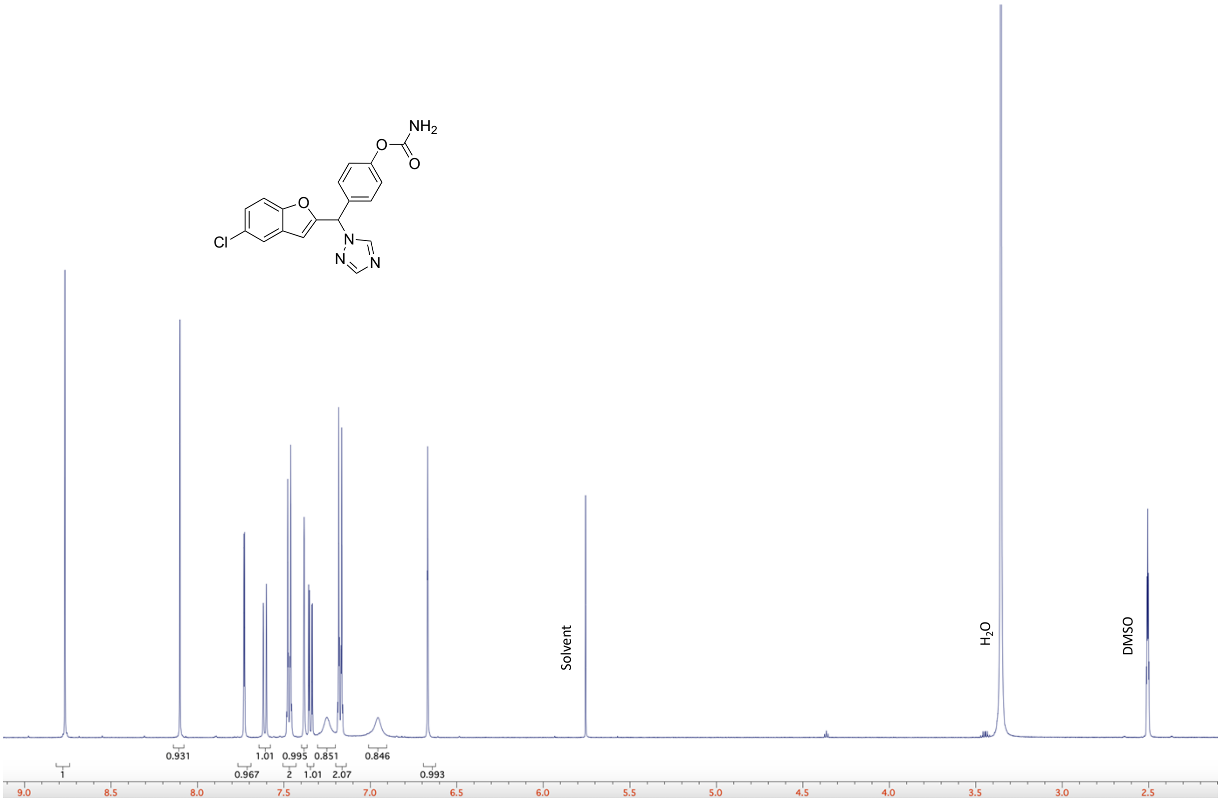

^{13}C NMR ($CDCl_3$)

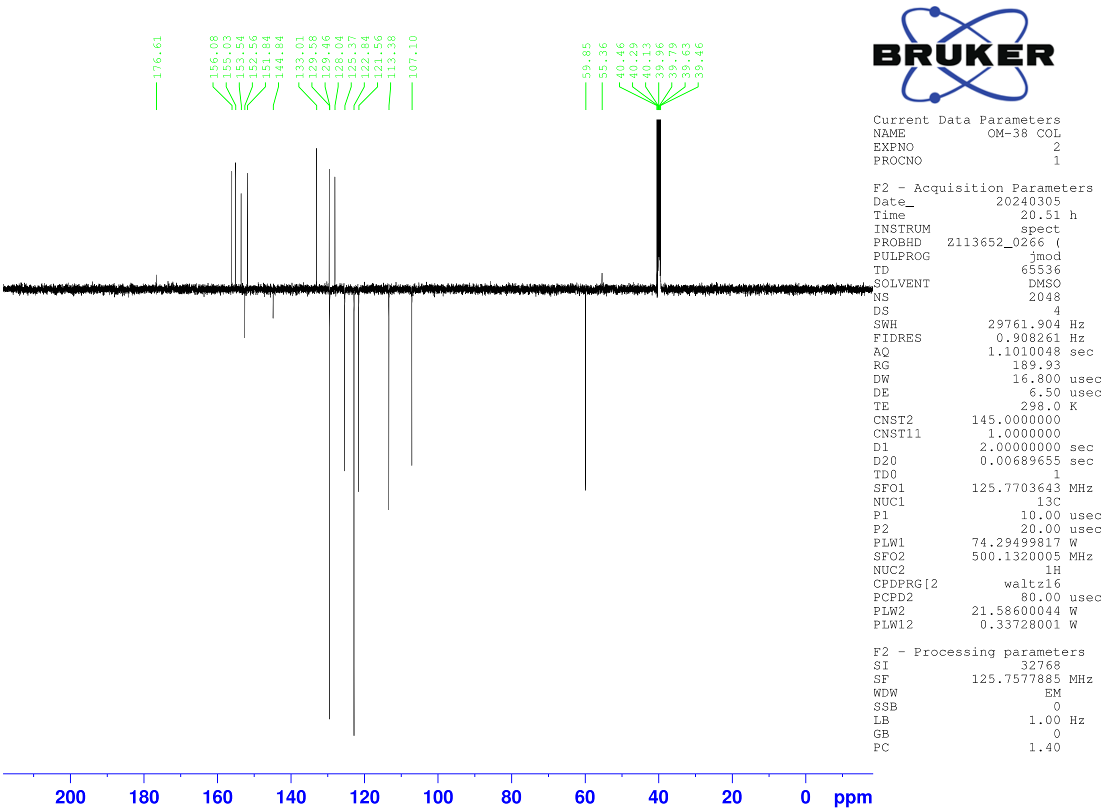

HPLC

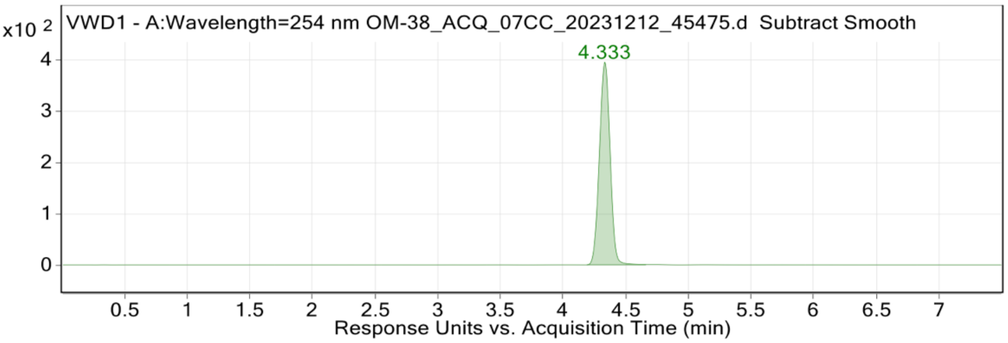

Figure: Base peak or HPLC chromatogram (indicated in left hand corner)

User Chromatogram Peak List

RT (min)	Area	Area %	Area Sum (%)	Symmetry	Width (min)
4.33	2354.83	100.00	100.00	1.02	0.467

Compound **9**

^{1}H NMR (DMSO-d_6)

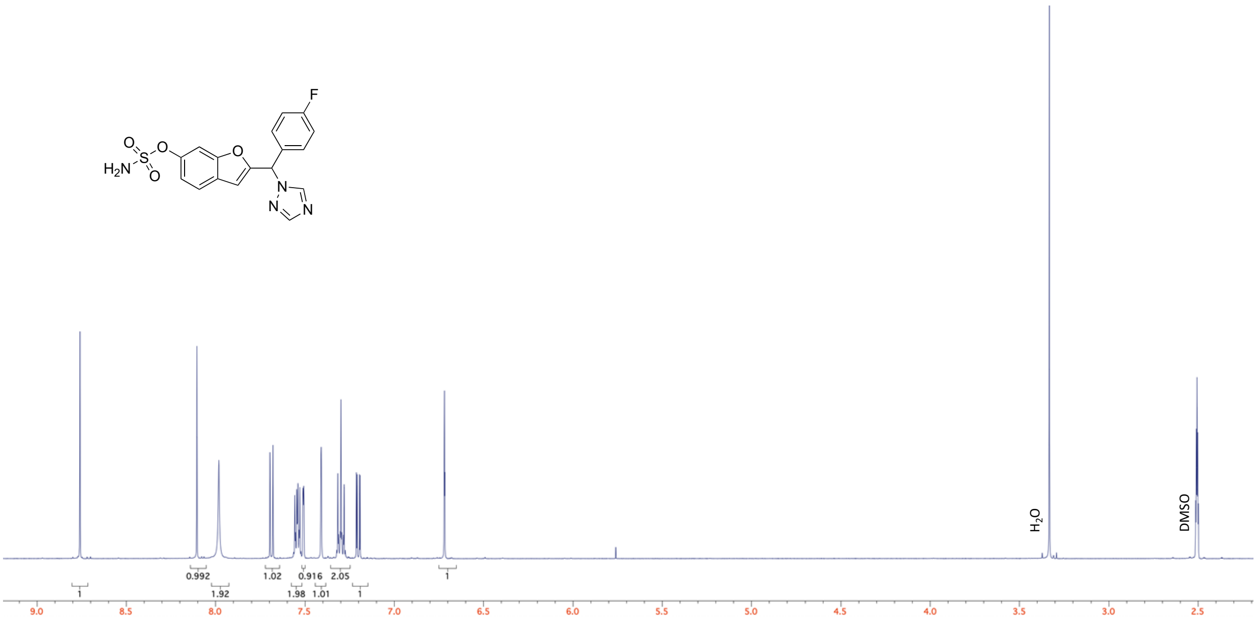

^{13}C NMR (DMSO-d_6)

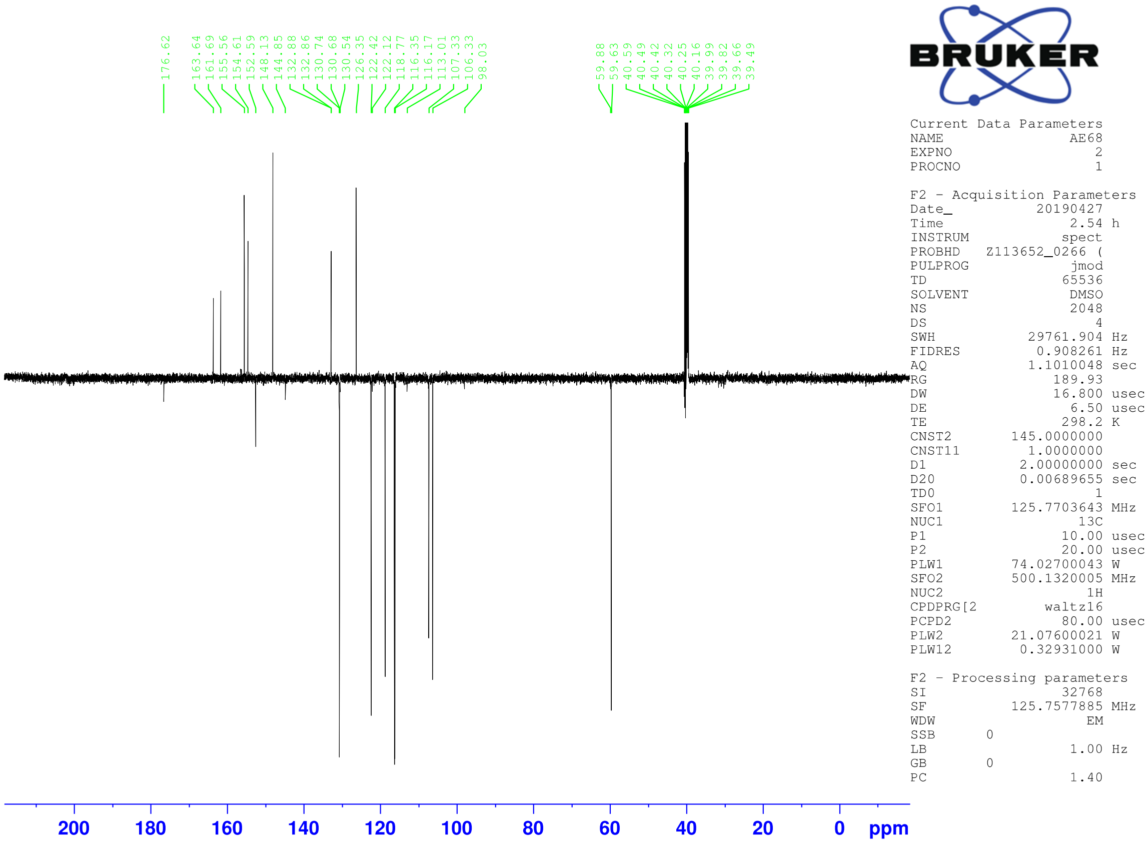


```
Current Data Parameters
NAME                 AE68
EXPNO                   2
PROCNO                  1

F2 - Acquisition Parameters
Date_           20190427
Time                2.54 h
INSTRUM            spect
PROBHD   Z113652_0266 (
PULPROG             jmod
TD                 65536
SOLVENT             DMSO
NS                  2048
DS                     4
SWH           29761.904 Hz
FIDRES         0.908261 Hz
AQ            1.1010048 sec
RG                189.93
DW                16.800 usec
DE                  6.50 usec
TE                 298.2 K
CNST2        145.0000000
CNST11         1.0000000
D1            2.00000000 sec
D20           0.00689655 sec
TD0                    1
SFO1         125.7703643 MHz
NUC1                 13C
P1                 10.00 usec
P2                 20.00 usec
PLW1         74.02700043 W
SFO2         500.1320005 MHz
NUC2                  1H
CPDPRG[2         waltz16
PCPD2              80.00 usec
PLW2         21.07600021 W
PLW12         0.32931000 W

F2 - Processing parameters
SI                 32768
SF           125.7577885 MHz
WDW                   EM
SSB       0
LB                  1.00 Hz
GB        0
PC                  1.40
```

HPLC

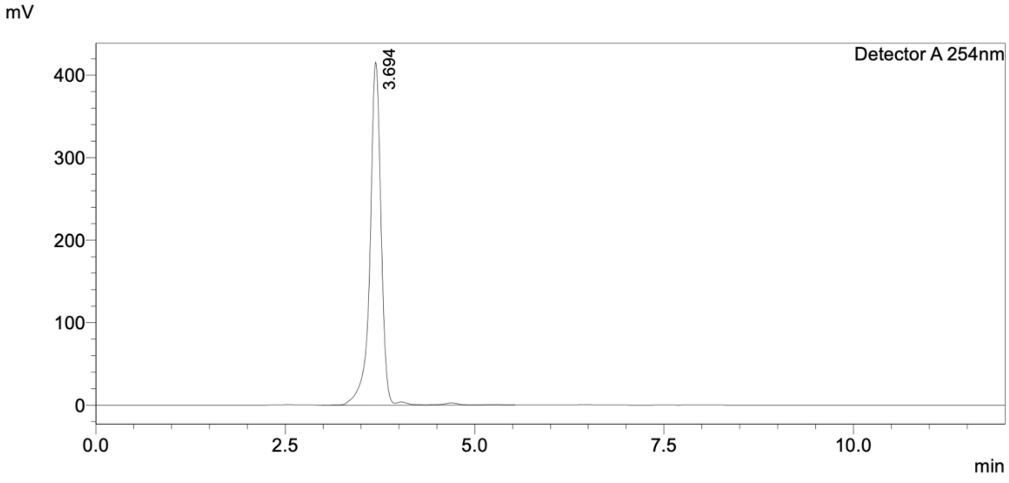

<Peak Table>

Detector A 254nm

Peak#	Ret. Time	Area	Height	Conc.	Area%
1	3.694	4250048	415523	100.000	100.000
Total		4250048	415523		100.000

Compound **10a**

^{1}H NMR (DMSO-d_6)

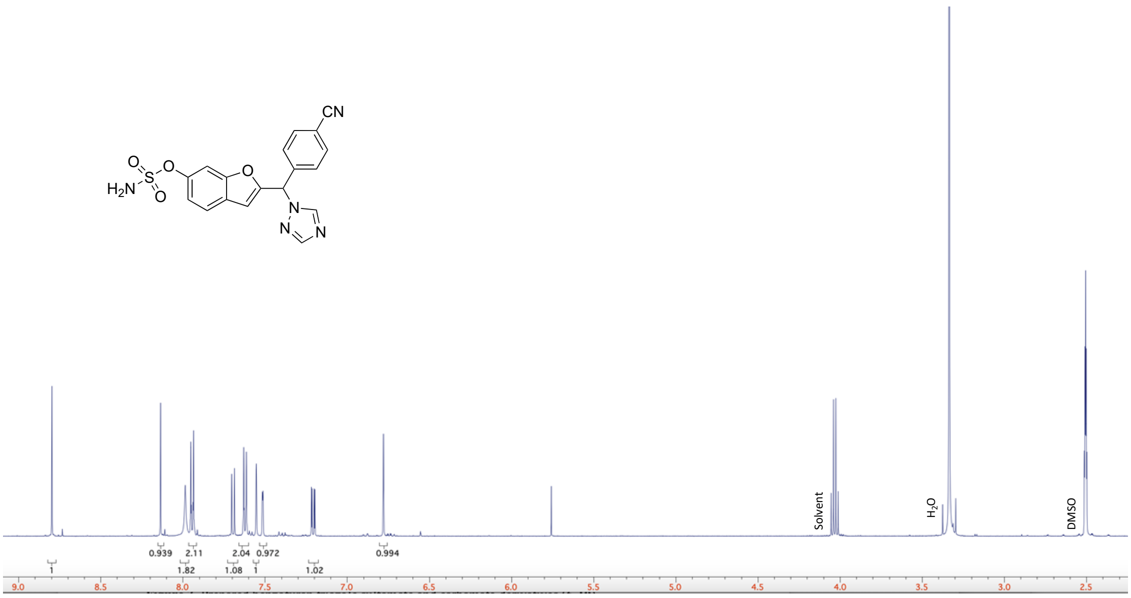

^{13}C NMR (DMSO-d_6)

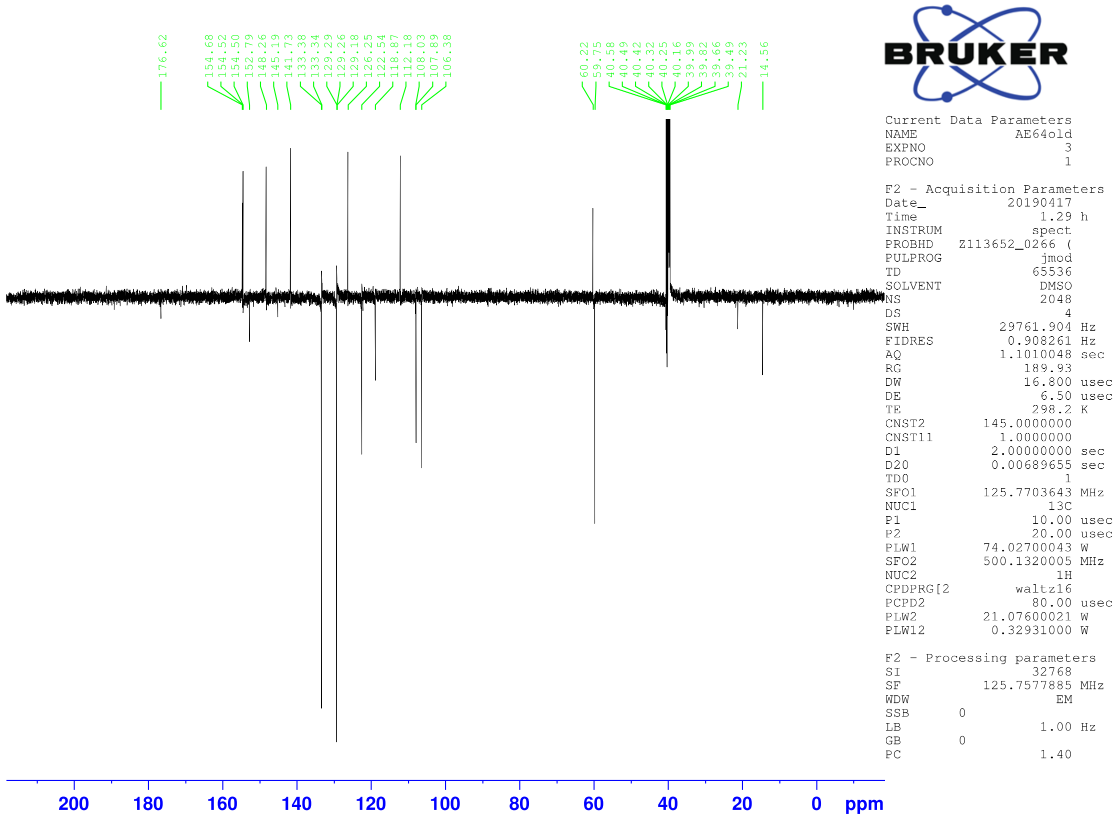

HPLC

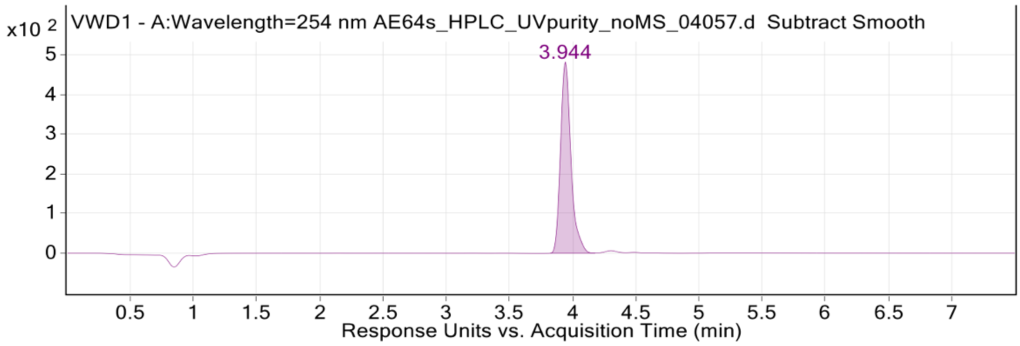

Figure: Base peak or HPLC chromatogram (indicated in left hand corner)

User Chromatogram Peak List

RT (min)	Area	Area %	Area Sum (%)	Symmetry	Width (min)
3.94	2777.07	100.00	100.00	1.43	0.352

Compound **10b**

^{1}H NMR (DMSO-d_6)

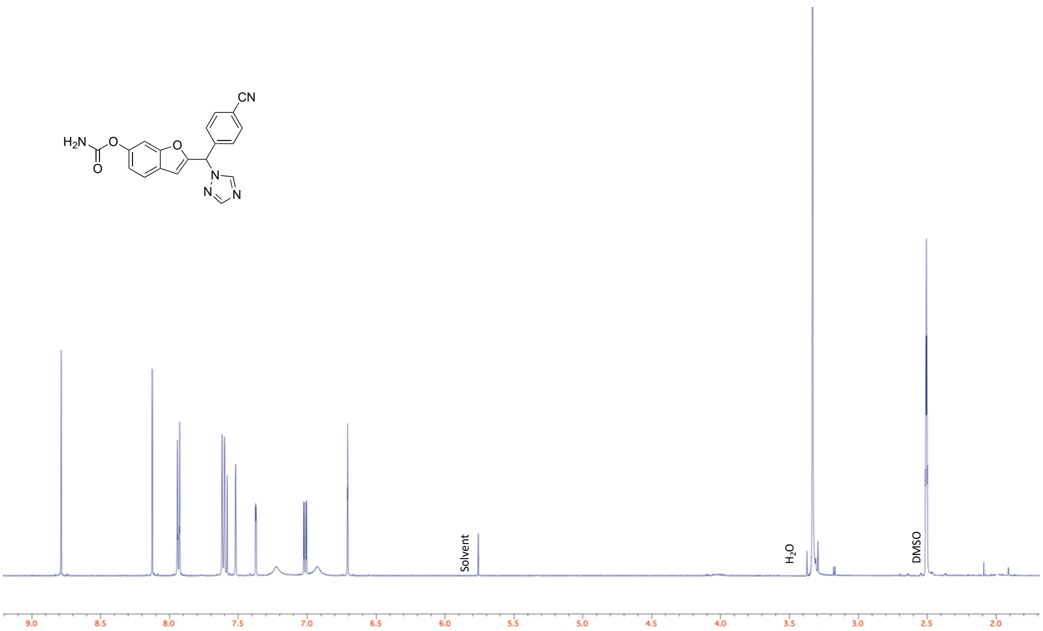

^{13}C NMR (DMSO-d_6)

HPLC

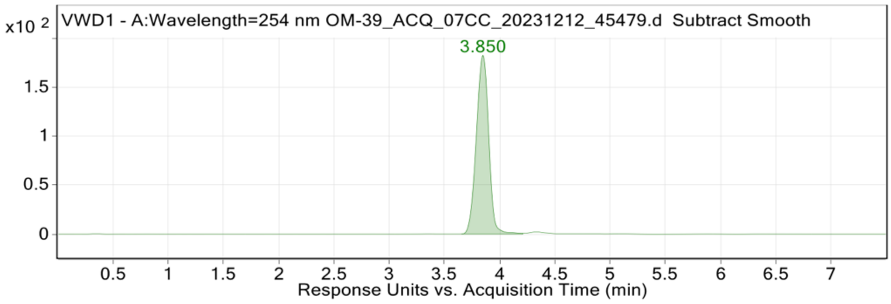

Figure: Base peak or HPLC chromatogram (indicated in left hand corner)

User Chromatogram Peak List

RT (min)	Area	Area %	Area Sum (%)	Symmetry	Width (min)
3.85	1427.25	100.00	100.00	0.85	0.553

Compound **18a**

^{1}H NMR (DMSO-d_6)

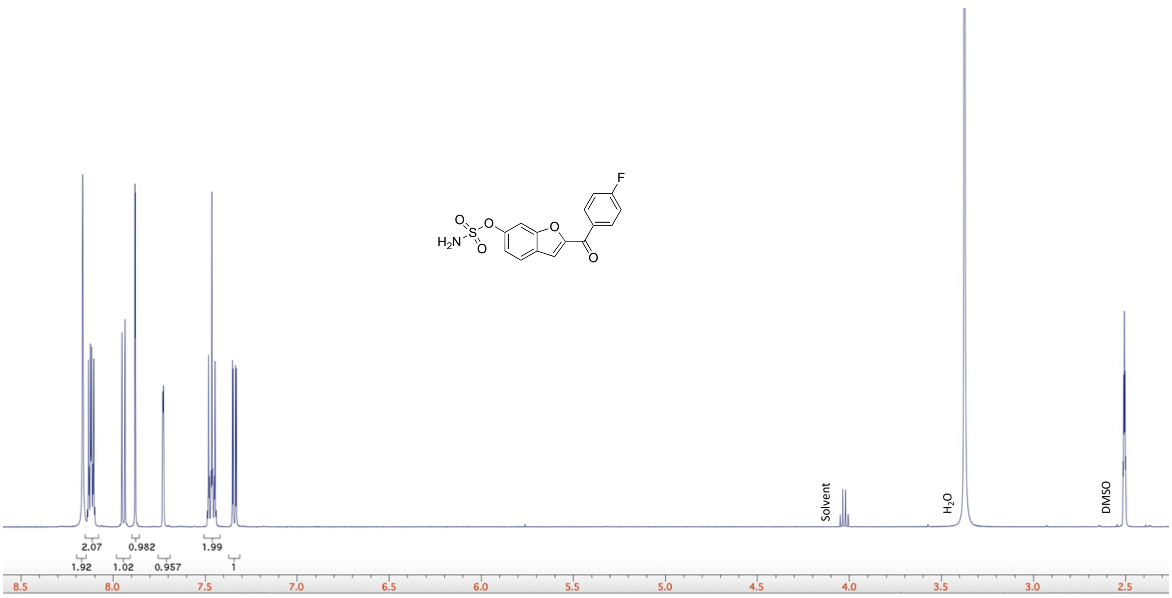

^{13}C NMR (DMSO-d_6)

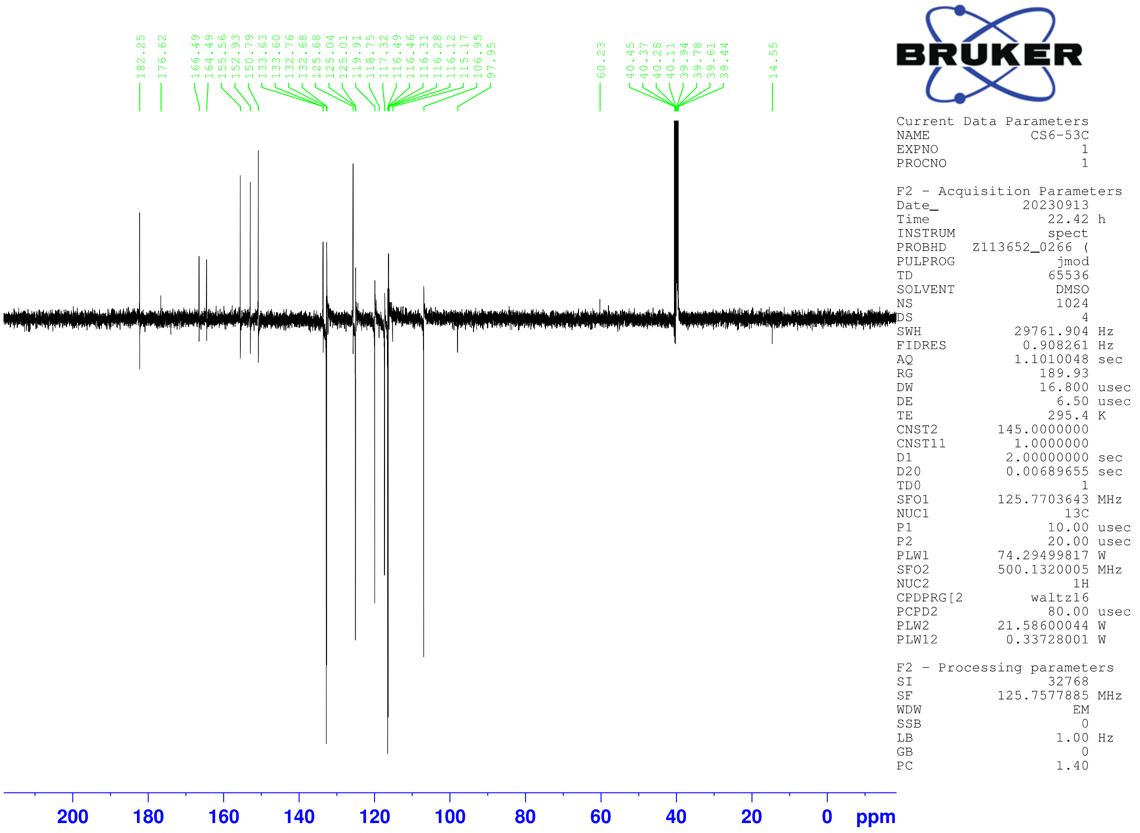

^{19}F NMR

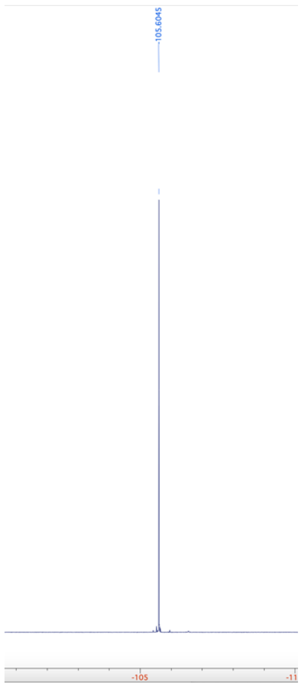

Compound **18b**

^{1}H NMR (DMSO-d_6)

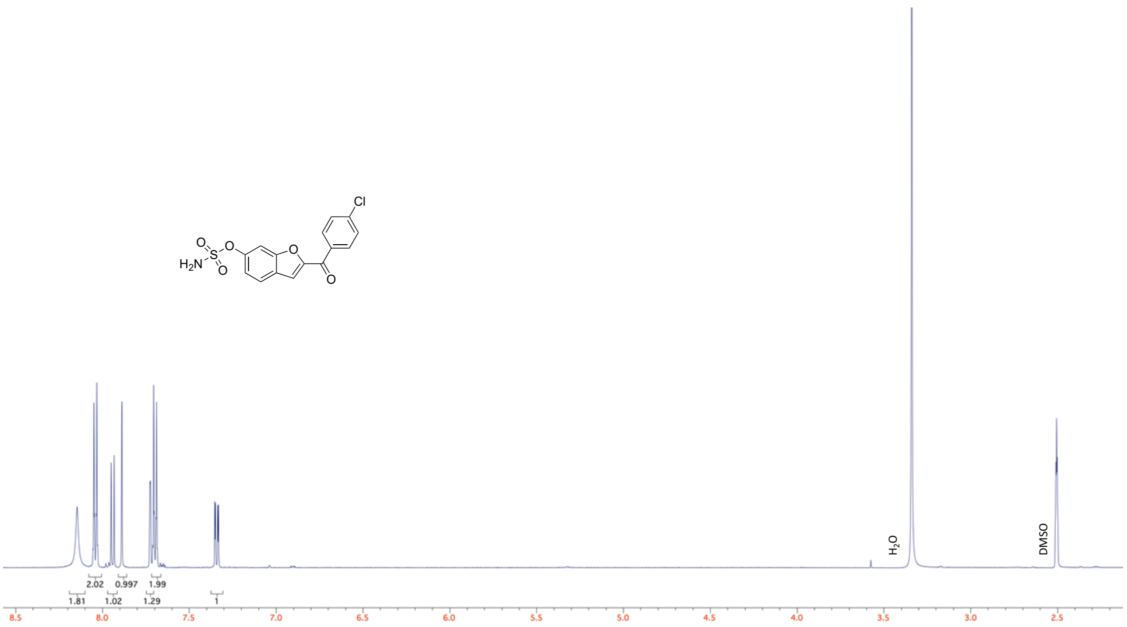

^{13}C NMR (DMSO-d_6)

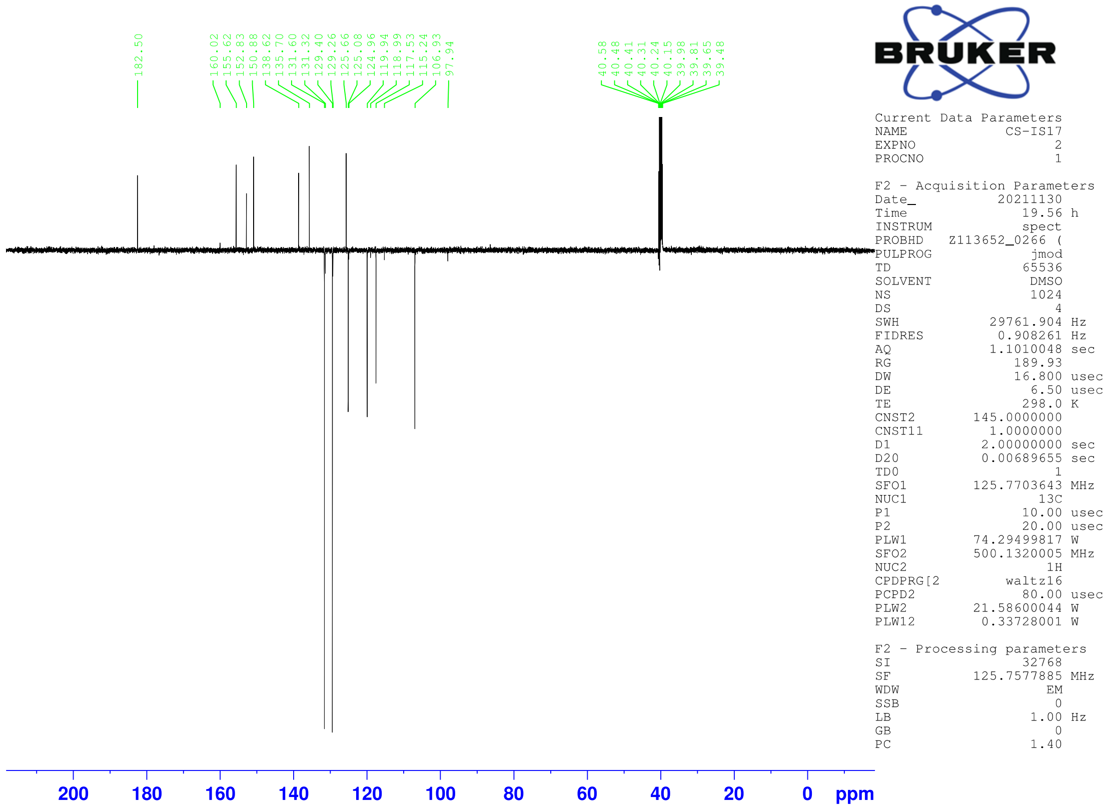

Compound **18c**

^{1}H NMR (DMSO-d_6)

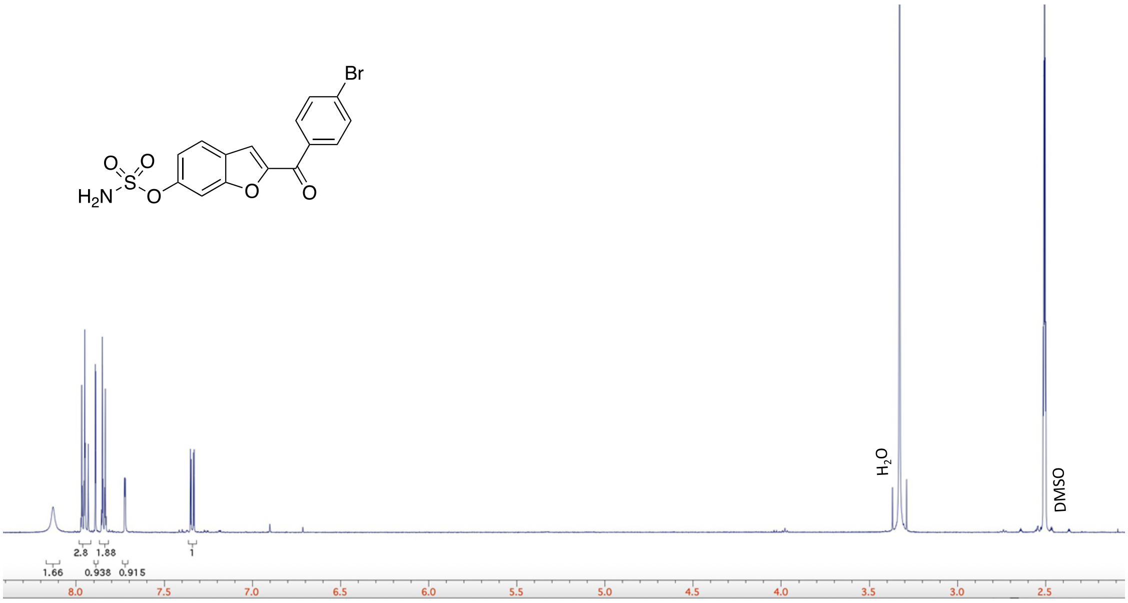

^{13}C NMR (DMSO-d_6)

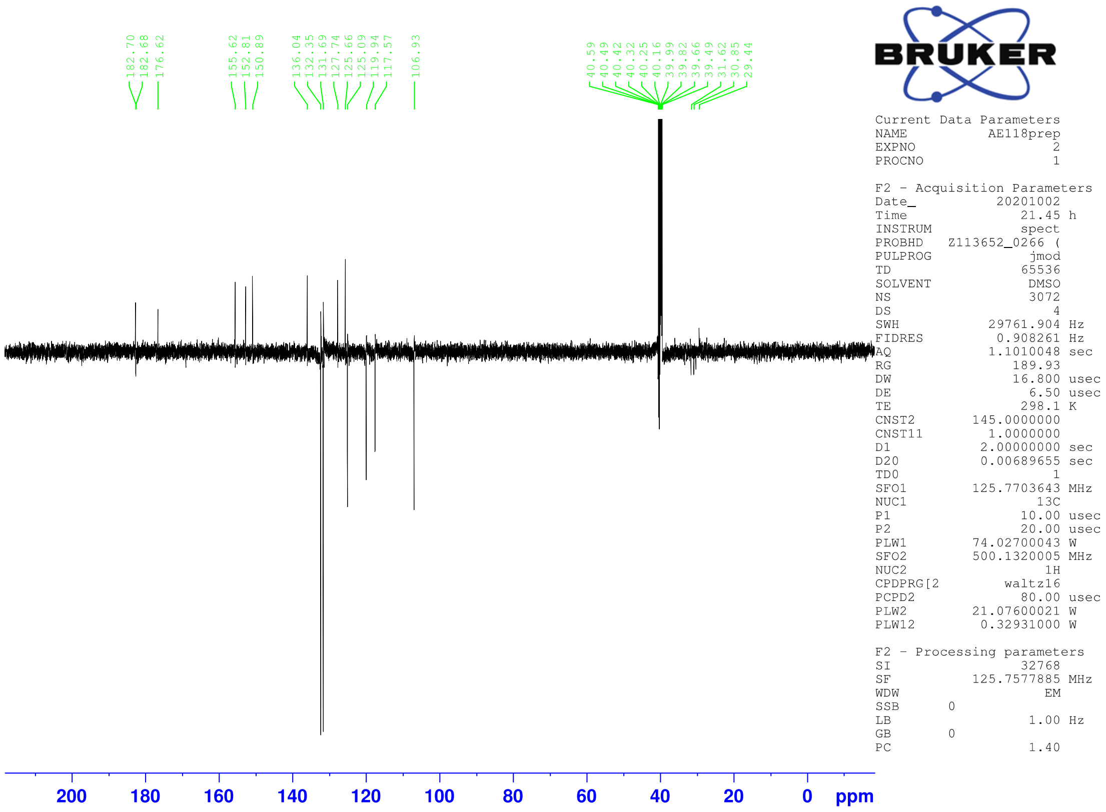

HPLC

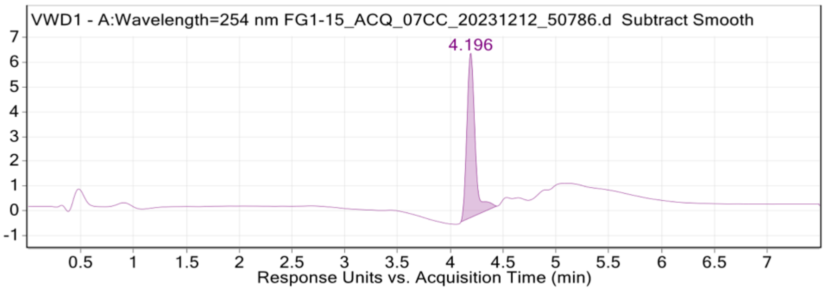

Figure: Base peak or HPLC chromatogram (indicated in left hand corner)

User Chromatogram Peak List

RT (min)	Area	Area %	Area Sum (%)	Symmetry	Width (min)
4.20	36.13	100.00	100.00	1.35	0.338

Compound **18d**

^{1}H NMR (DMSO-d_6)

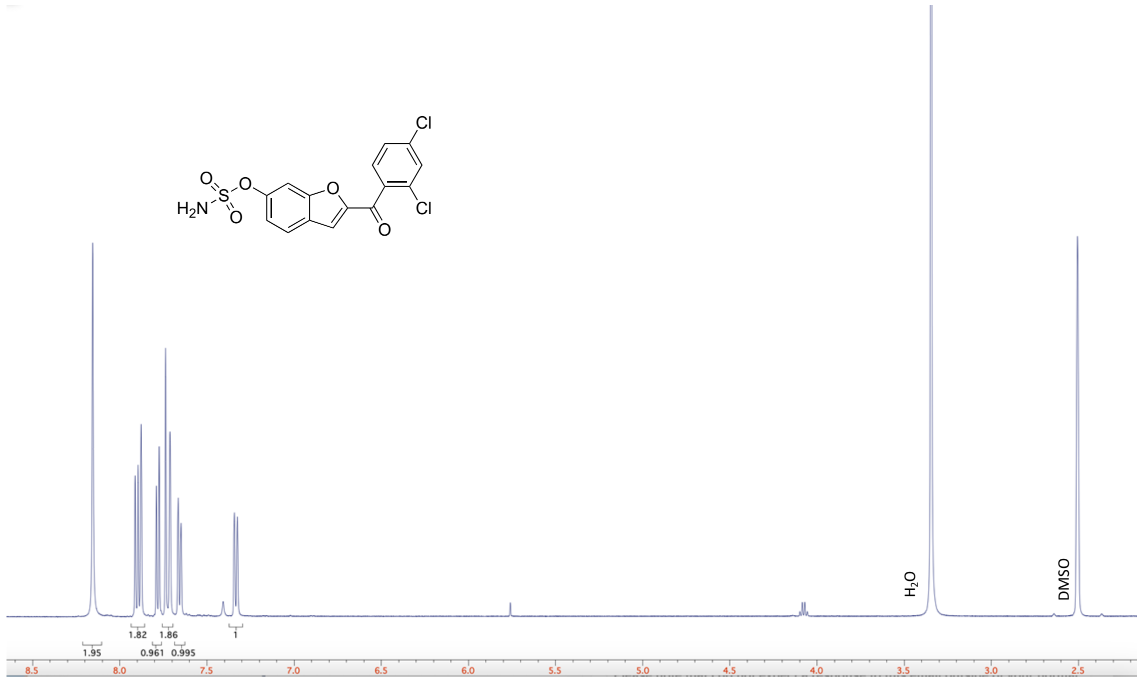

^{13}C NMR (DMSO-d_6)

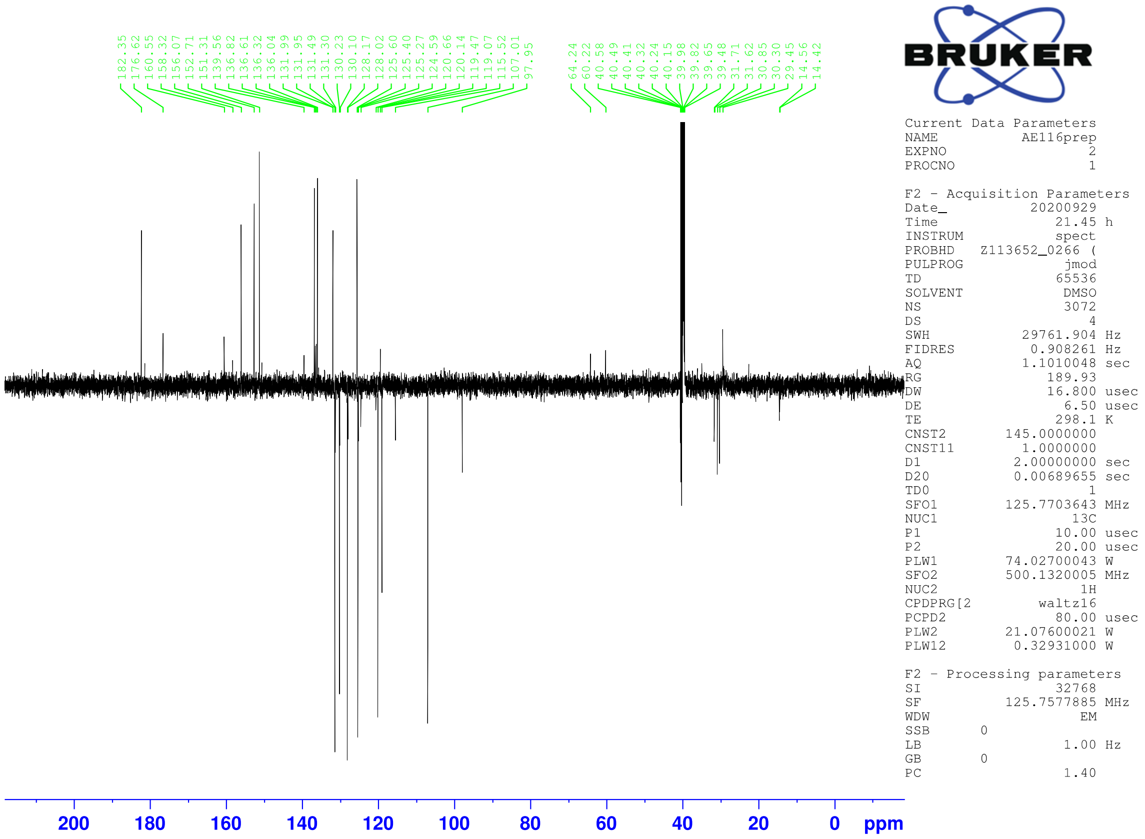

Compound **18e**

^{1}H NMR (DMSO-d_6)

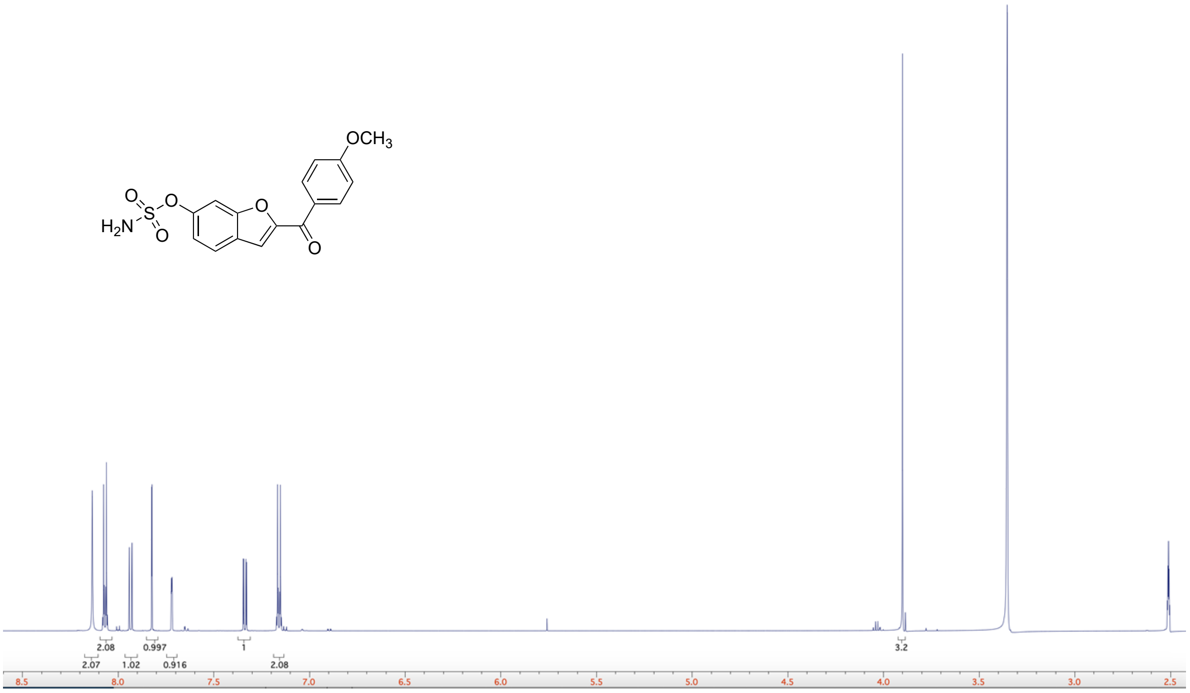

^{13}C NMR (DMSO-d_6)

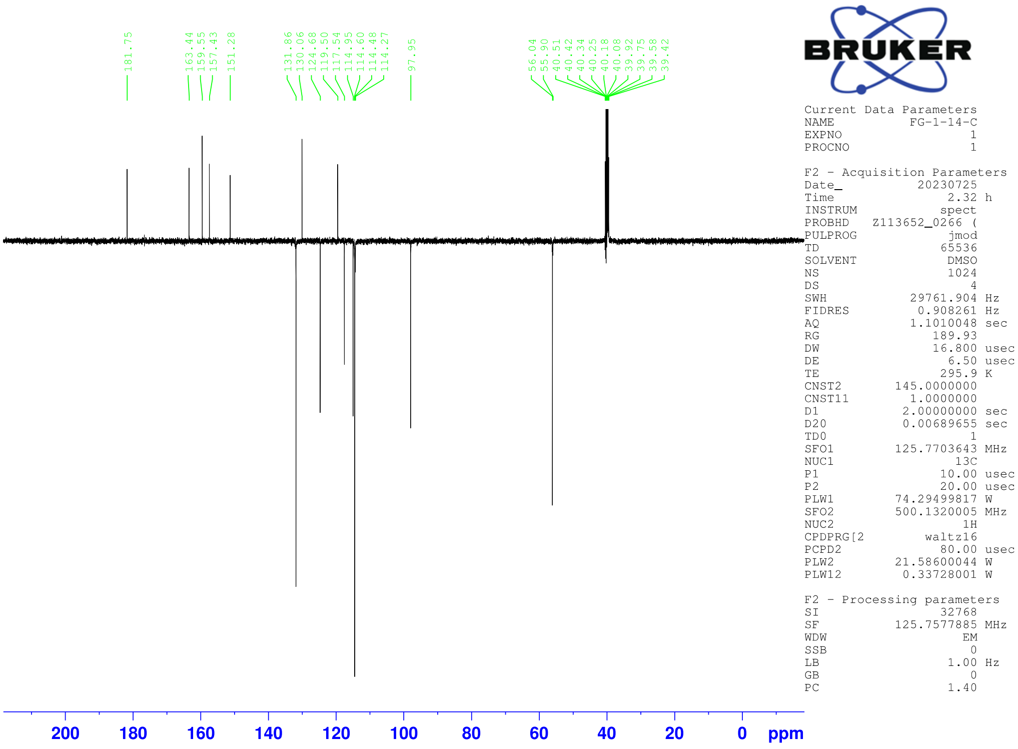

HPLC

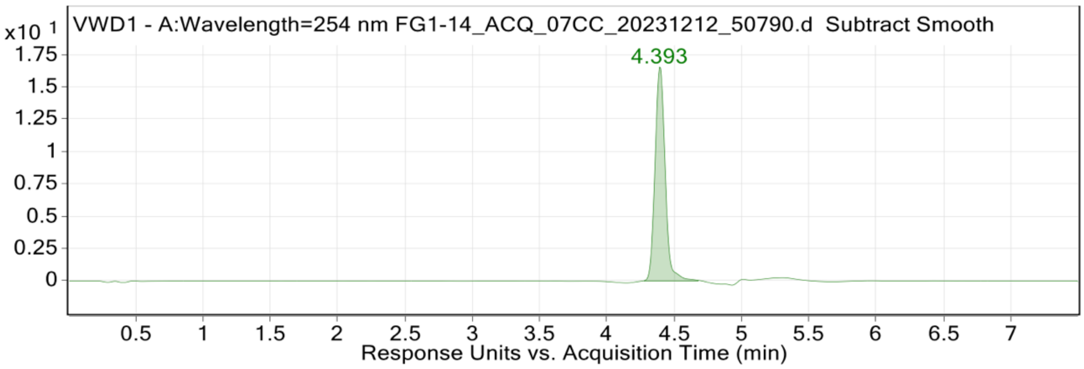

Figure: Base peak or HPLC chromatogram (indicated in left hand corner)

User Chromatogram Peak List

RT (min)	Area	Area %	Area Sum (%)	Symmetry	Width (min)
4.39	87.25	100.00	100.00	1.18	0.400

Compound **18f**

^{1}H NMR (DMSO-d_6)

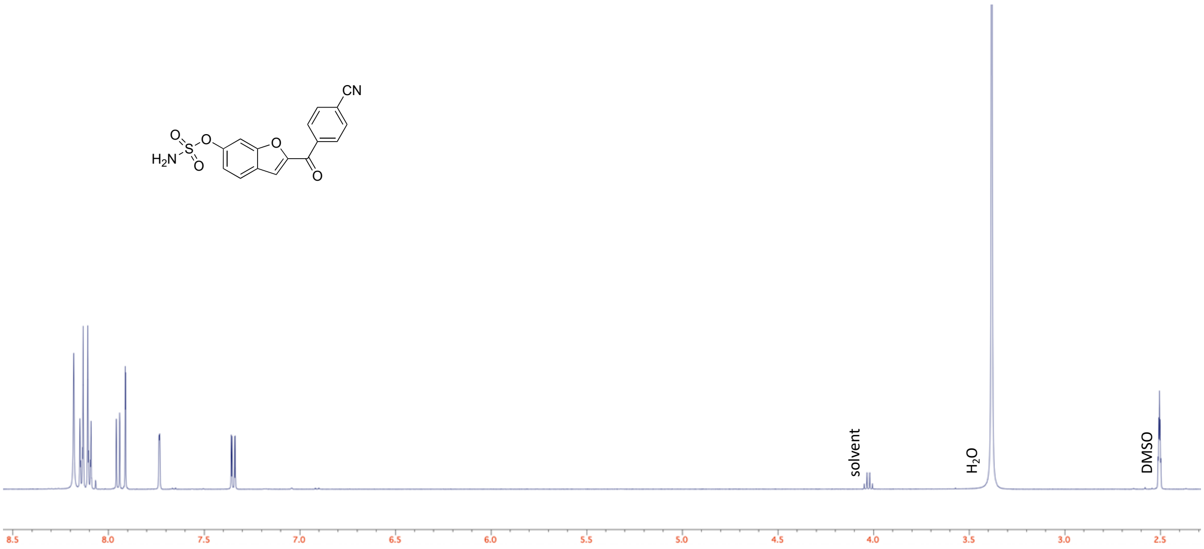

^{13}C NMR (DMSO-d_6)

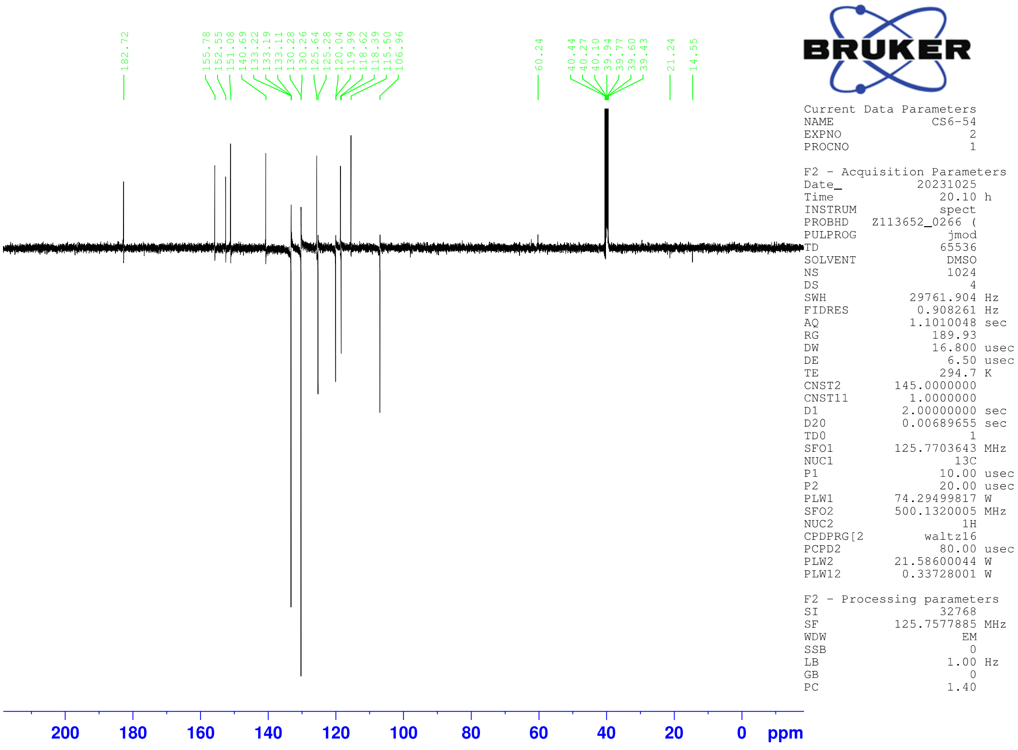

HPLC

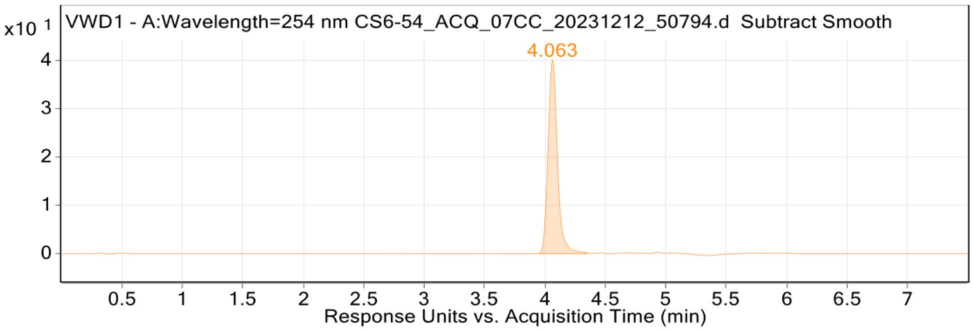

Figure: Base peak or HPLC chromatogram (indicated in left hand corner)

User Chromatogram Peak List

RT (min)	Area	Area %	Area Sum (%)	Symmetry	Width (min)
4.06	217.89	100.00	100.00	1.23	0.407

Compound **19a**

^{1}H NMR (DMSO-d_6)

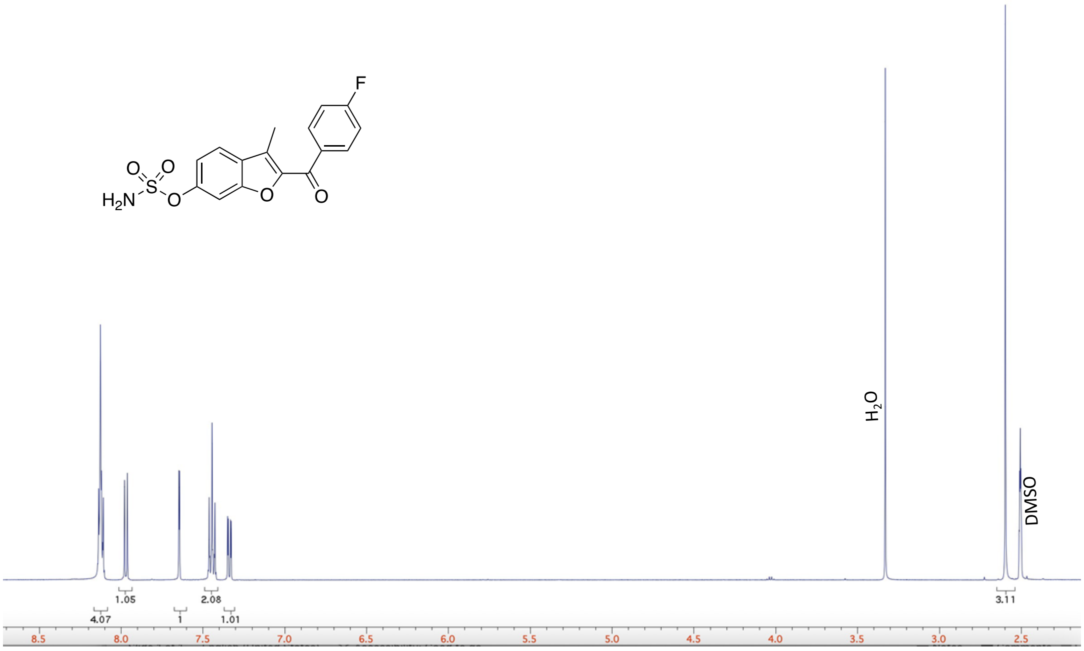

^{13}C NMR (DMSO-d_6)

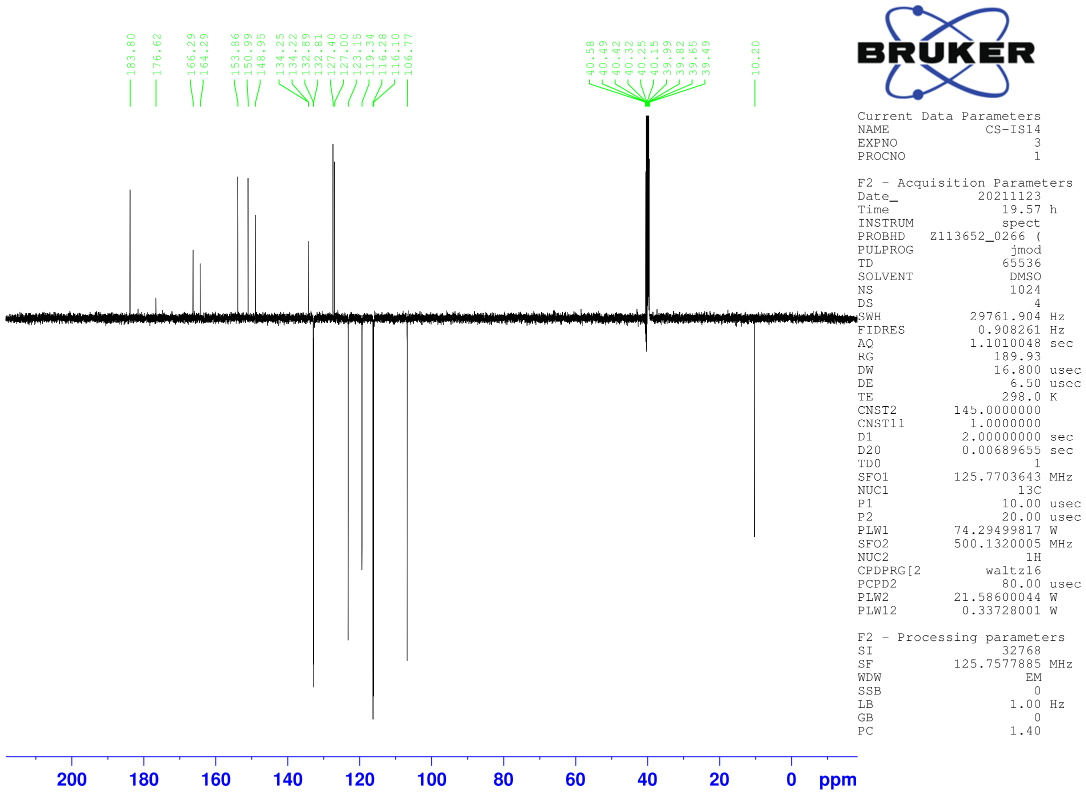

^{19}F NMR (DMSO-d_6)

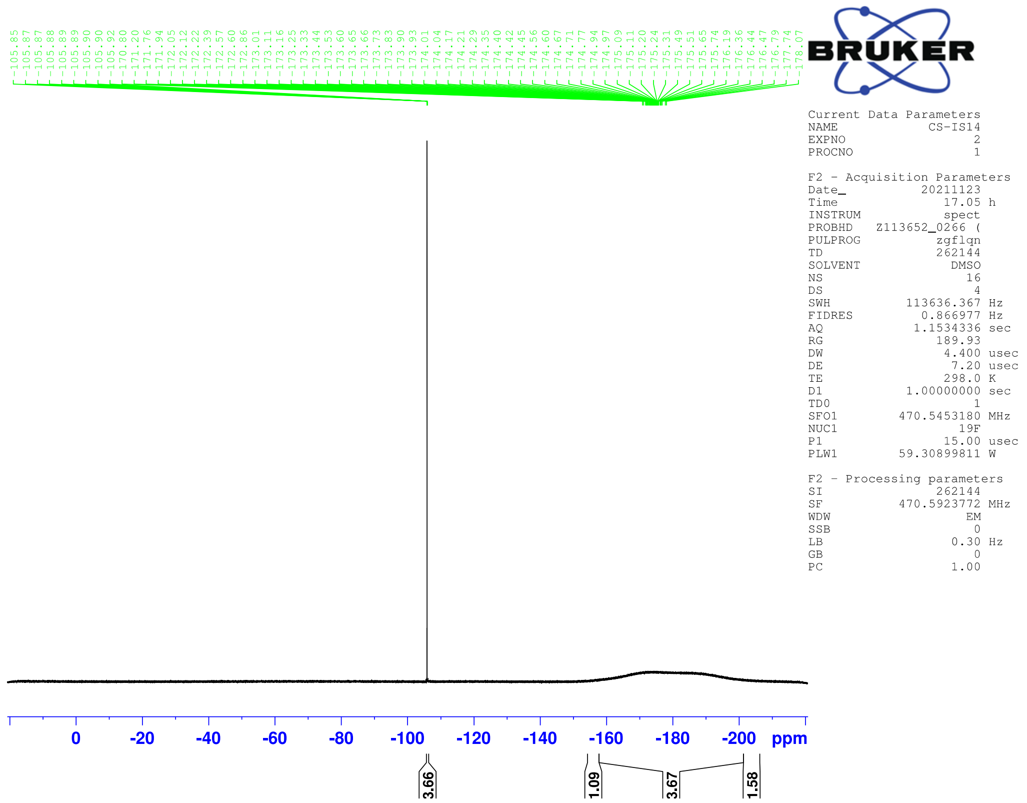

Compound **19b**

^{1}H NMR (DMSO-d_6)

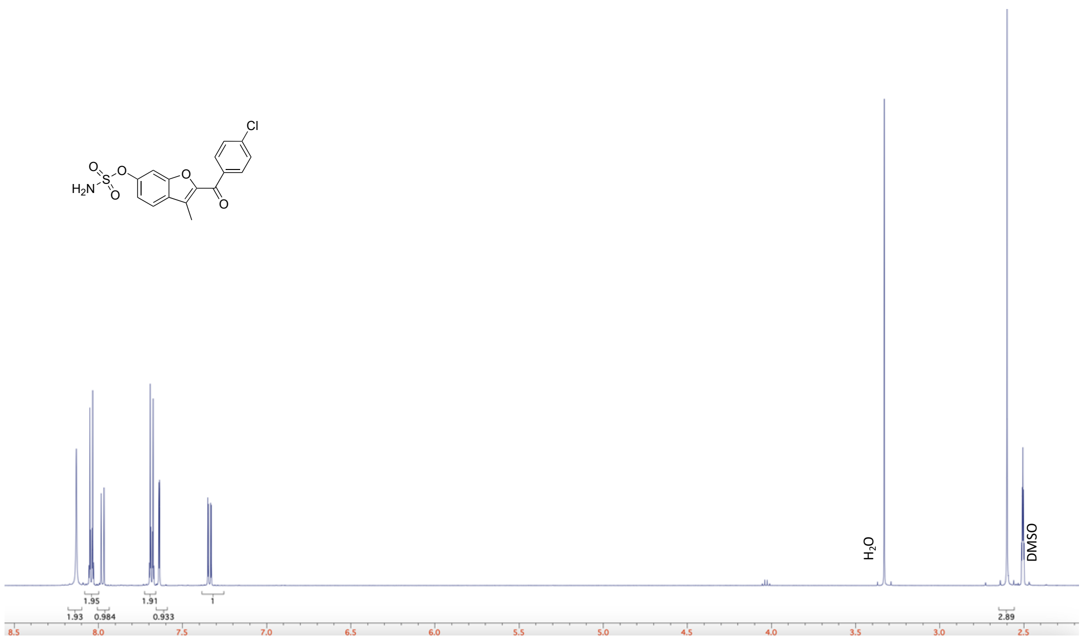

^{13}C NMR (DMSO-d_6)

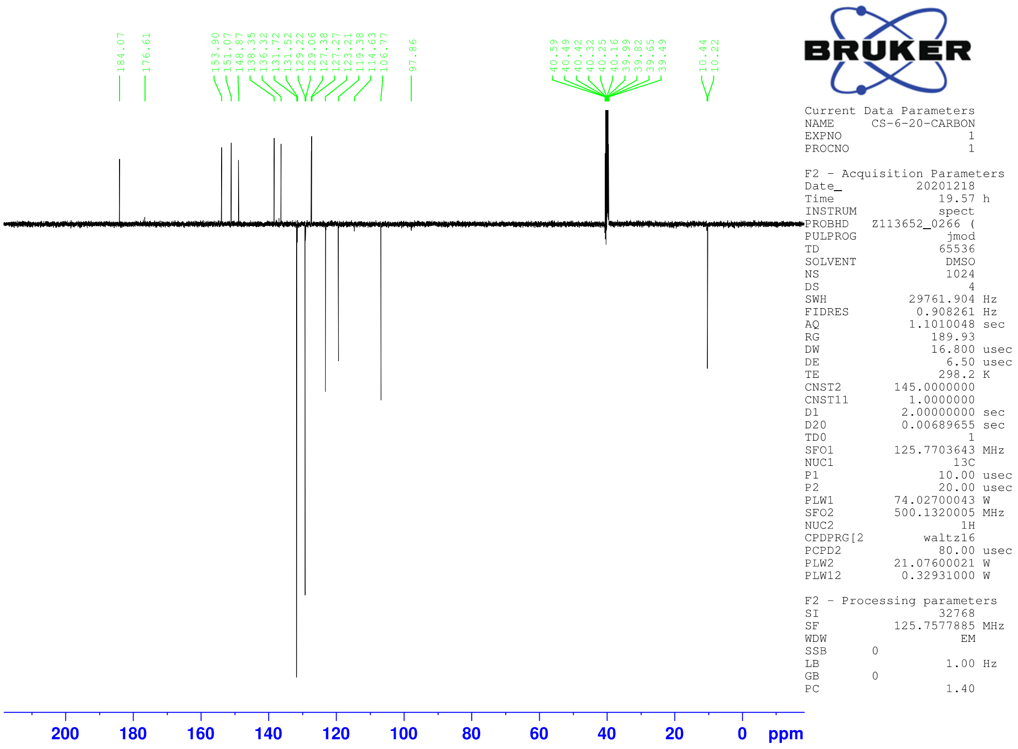

Compound **19c**

^{1}H NMR (DMSO-d_6)

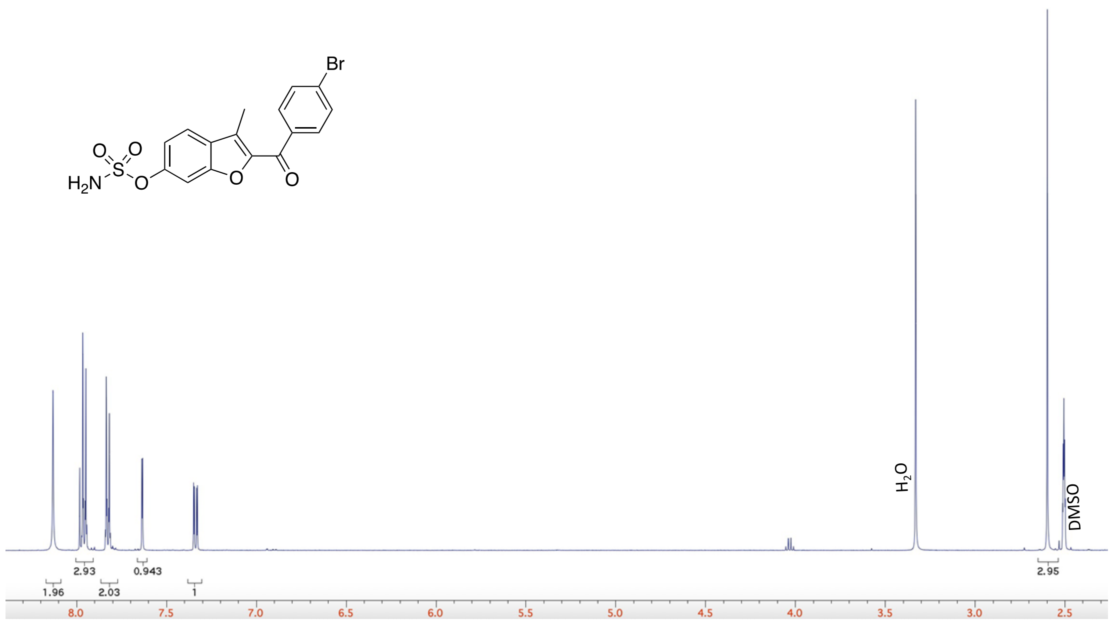

^{13}C NMR (DMSO-d_6)

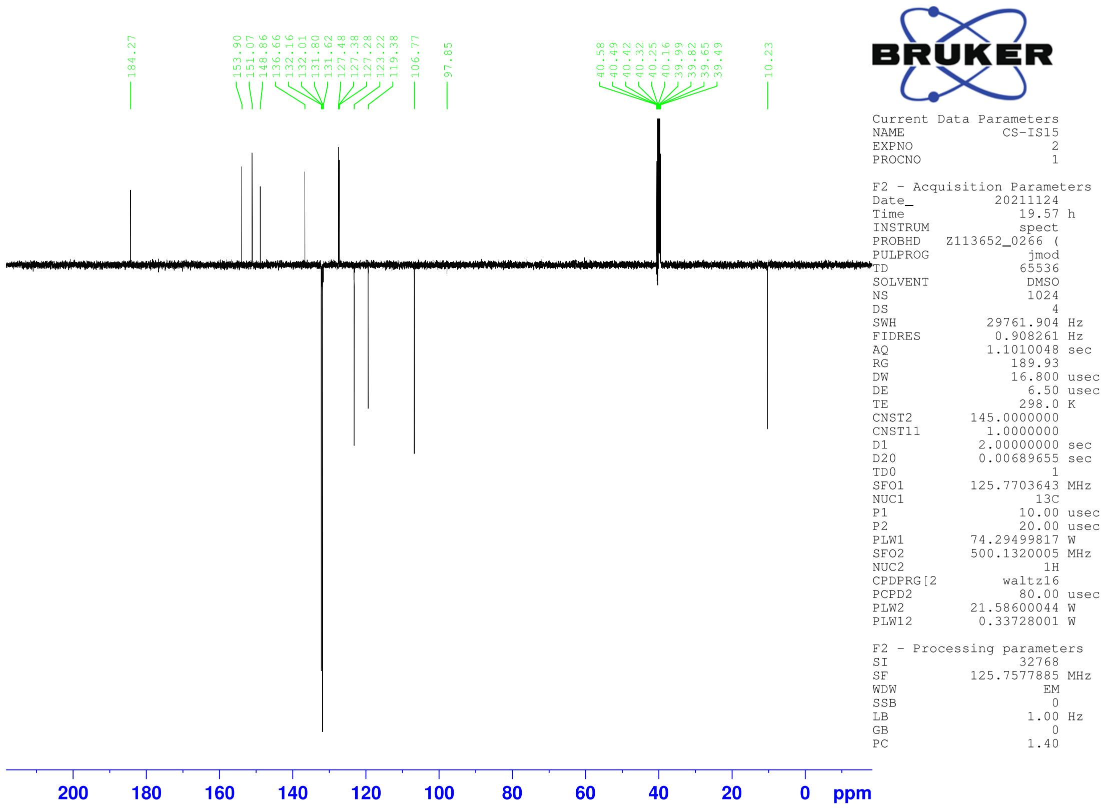

Compound **19d**

^{1}H NMR (DMSO-d_6)

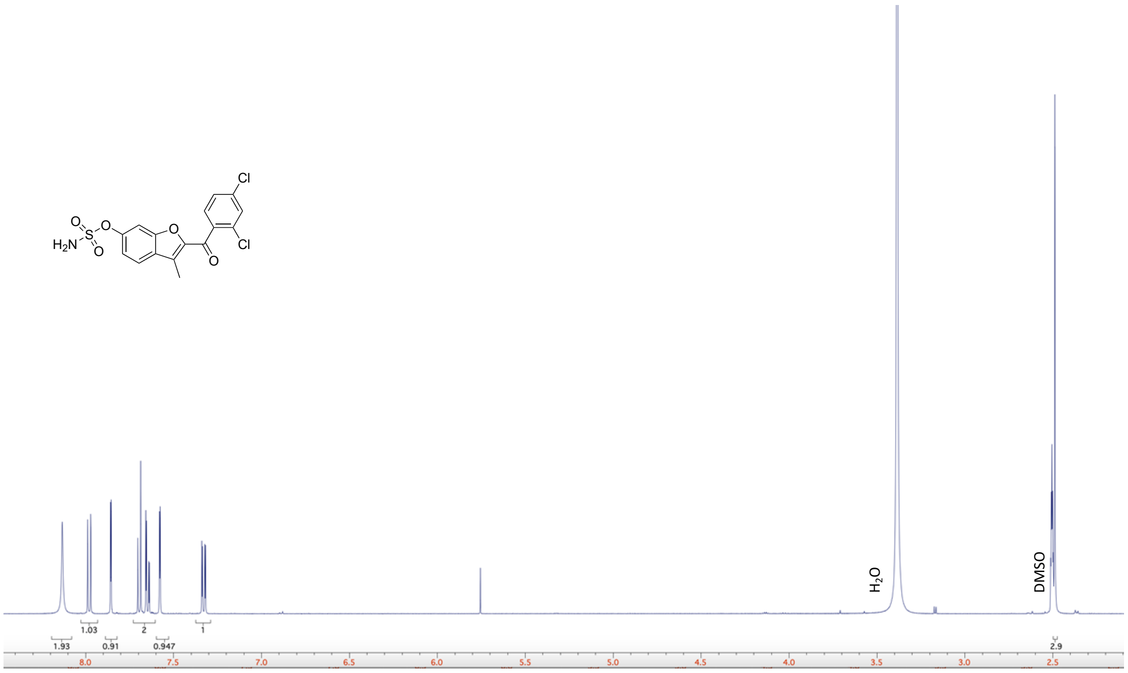

^{13}C NMR (DMSO-d_6)

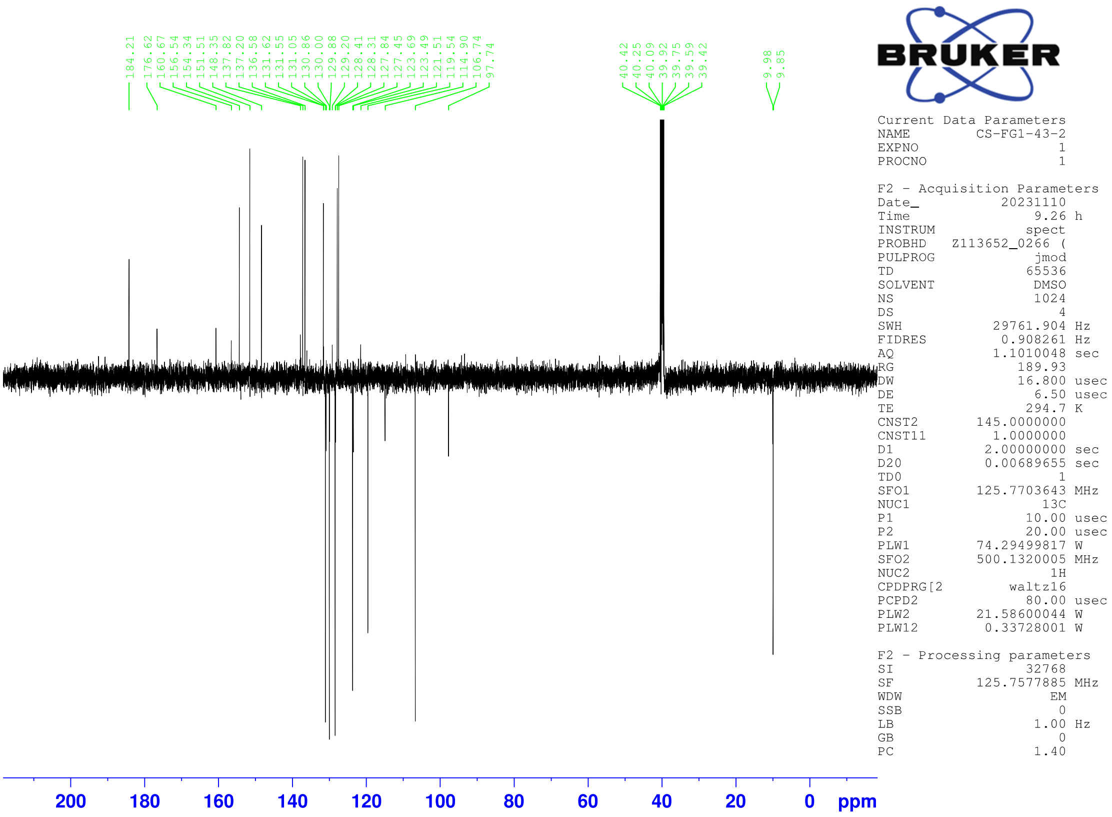

Compound **19e**

^{1}H NMR (DMSO-d_6)

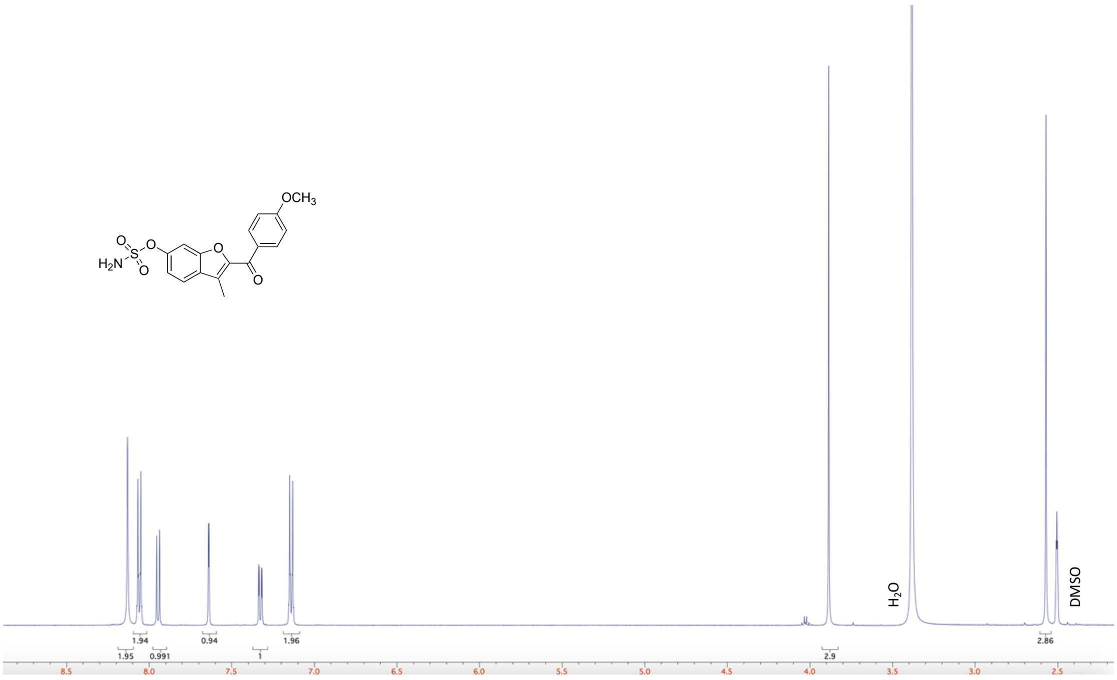

^{13}C NMR (DMSO-d_6)

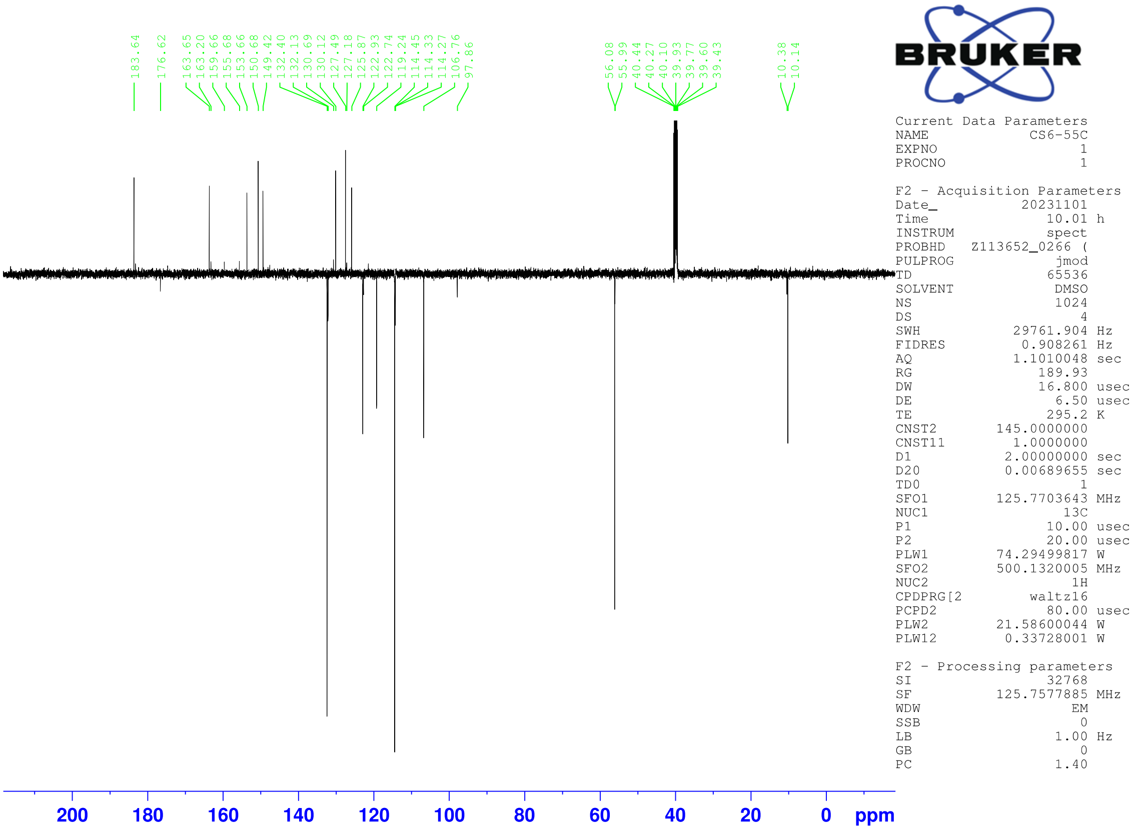

HPLC

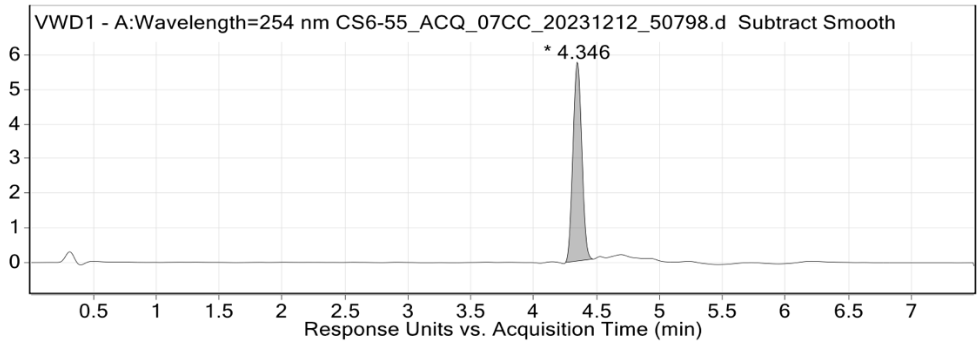

Figure: Base peak or HPLC chromatogram (indicated in left hand corner)

User Chromatogram Peak List

RT (min)	Area	Area %	Area Sum (%)	Symmetry	Width (min)
4.35	28.19	100.00	100.00	1.16	0.217

Compound **20**

^{1}H NMR (DMSO-d_6)

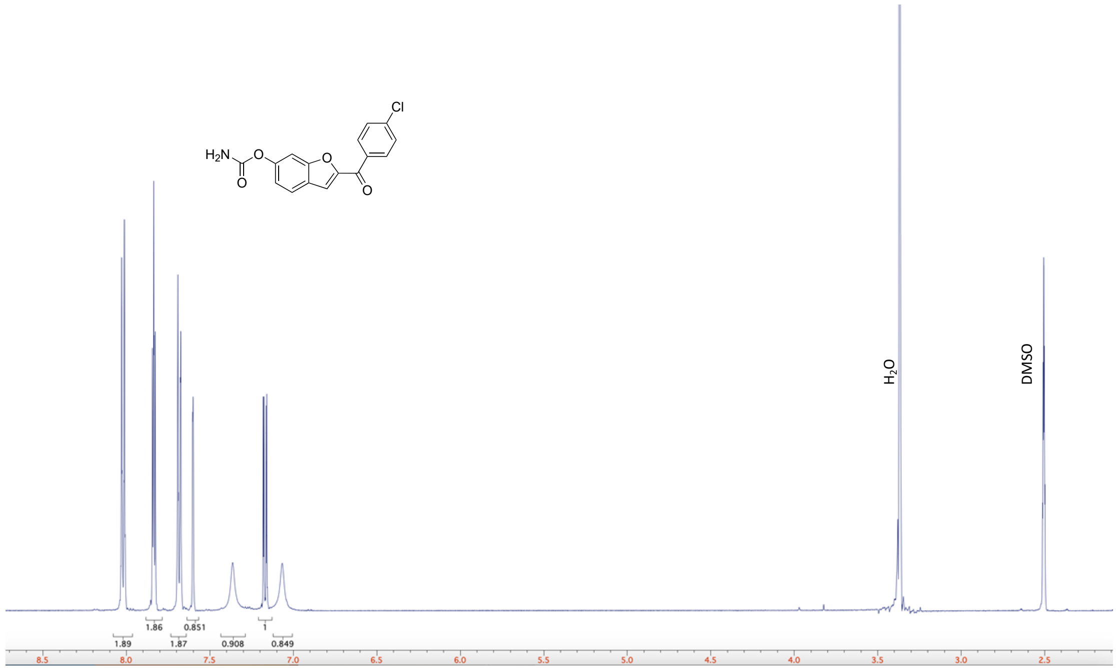

^{13}C NMR (DMSO-d_6)

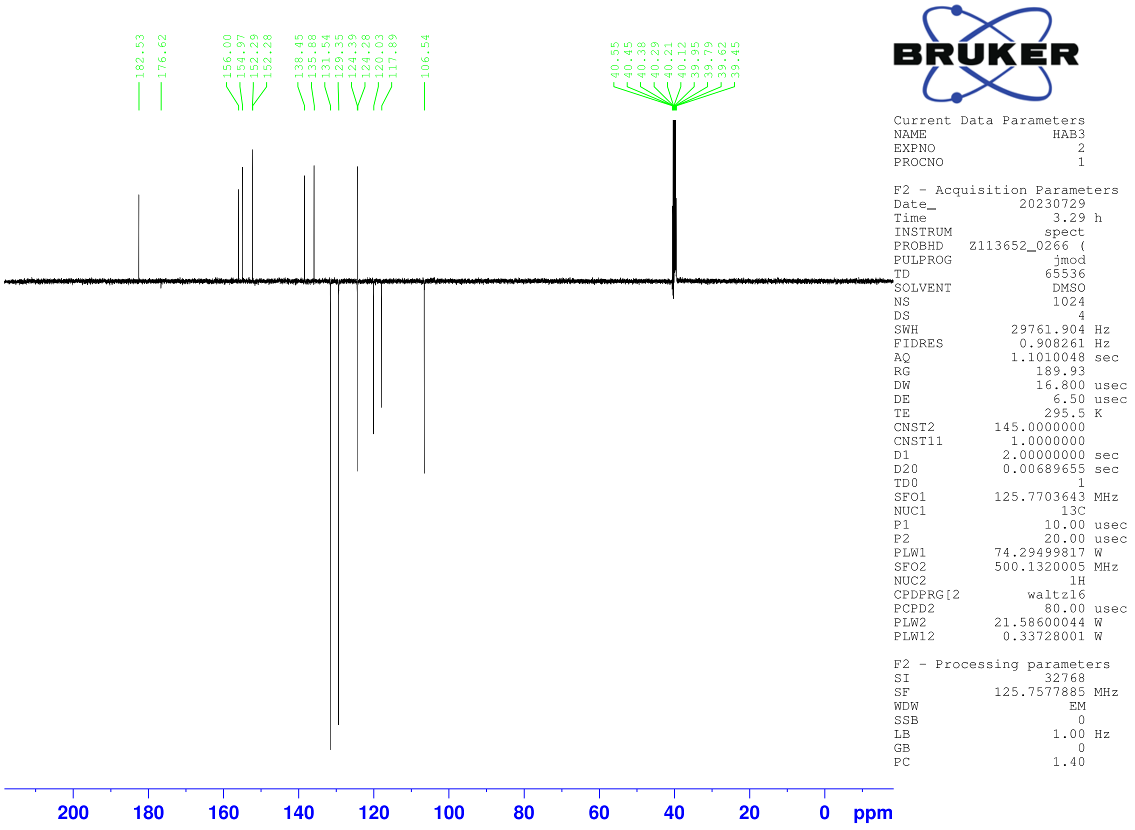

Compound **21**

^{1}H NMR (DMSO-d_6)

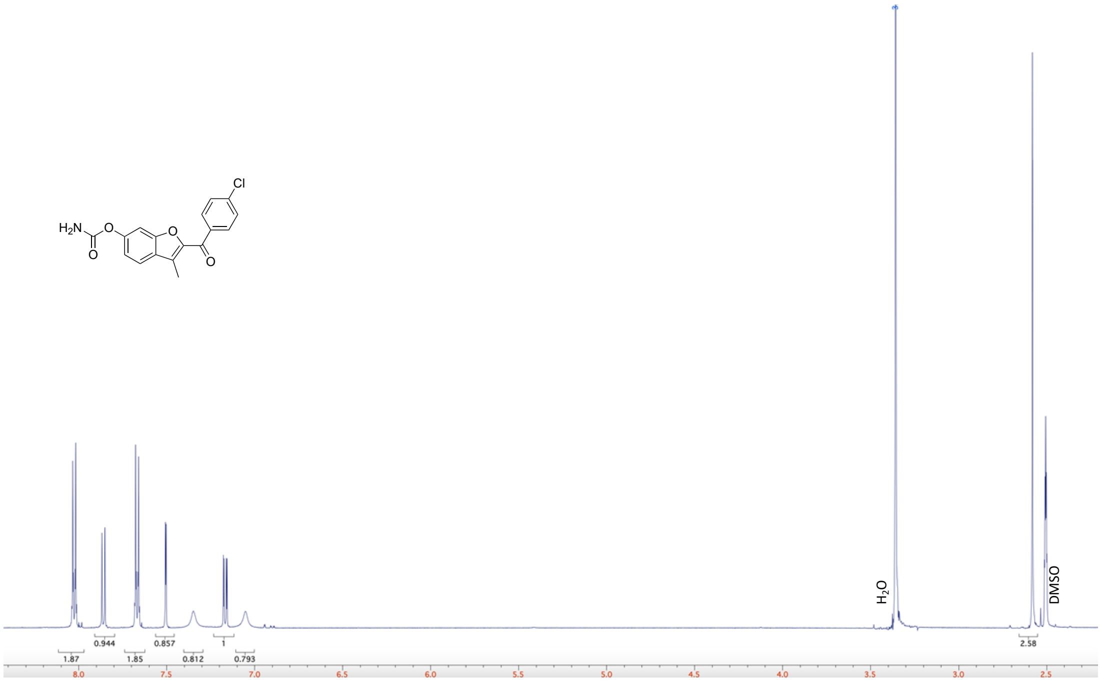

^{13}C NMR (DMSO-d_6)

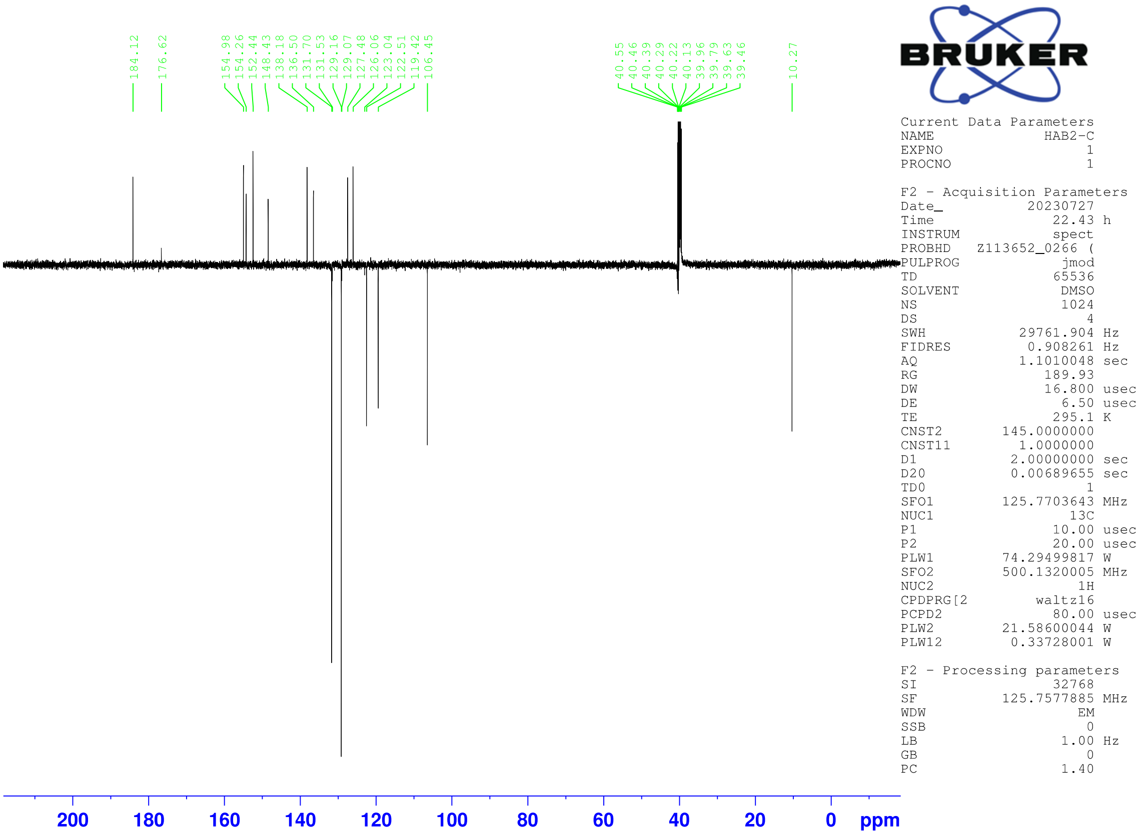

Supplement: MD-016-D4MD00795F-s001 [file MD-016-D4MD00795F-s001.pdf]
